# A convergent synthesis of 1,3,4-oxadiazoles from acyl hydrazides under semiaqueous conditions†
†Electronic supplementary information (ESI) available. See DOI: 10.1039/c7sc00195a



**DOI:** 10.1039/c7sc00195a

**Published:** 2017-02-23

**Authors:** Kazuyuki Tokumaru, Jeffrey N. Johnston

**Affiliations:** a Department of Chemistry , Vanderbilt Institute of Chemical Biology , Vanderbilt University , Nashville , Tennessee 37235 , USA . Email: jeffrey.n.johnston@vanderbilt.edu

## Abstract

The 1,3,4-oxadiazole is an aromatic heterocycle valued for its low-lipophilicity in drug development. Substituents at the 2- and/or 5-positions can modulate the heterocycle's electronic and hydrogen bond-accepting capability, while exploiting its use as a carbonyl bioisostere. A new approach to 1,3,4-oxadiazoles is described wherein α-bromo nitroalkanes are coupled to acyl hydrazides to deliver the 2,5-disubstituted oxadiazole directly, avoiding a 1,2-diacyl hydrazide intermediate. Access to new building blocks of oxadiazole-substituted secondary amines is improved by leveraging chiral α-bromo nitroalkane or amino acid hydrazide substrates. The non-dehydrative conditions for oxadiazole synthesis are particularly notable, in contrast to alternatives reliant on highly oxophilic reagents to effect cyclization of unsymmetrical 1,2-diacyl hydrazides. The mild conditions are punctuated by the straightforward removal of co-products by a standard aqueous wash.

## Abstract 2

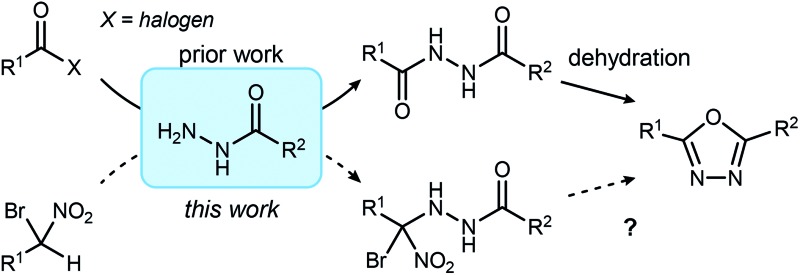
An innovative new synthesis approach to disubstituted 1,3,4-oxadiazoles is described, inspired by Umpolung Amide Synthesis (UmAS).

## Introduction

**Fig. 1:**
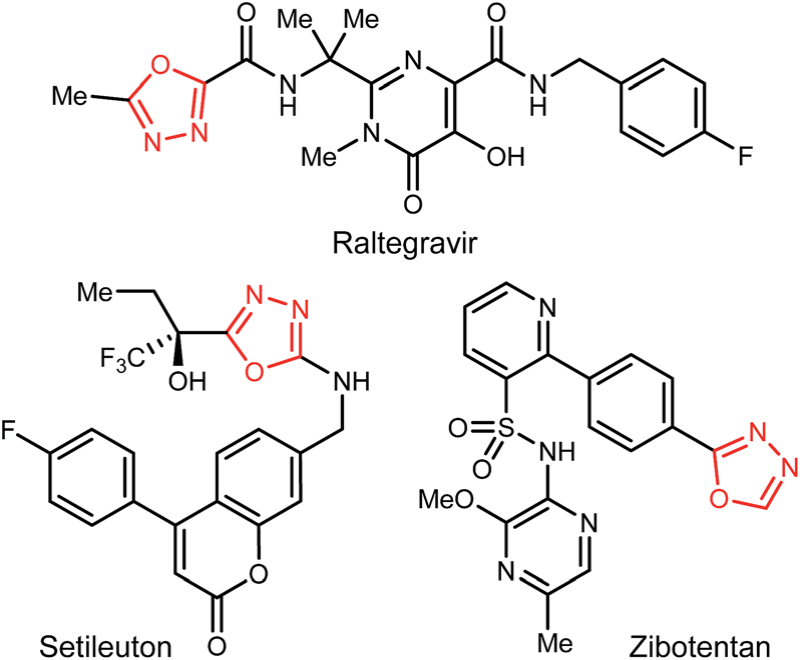
1,3,4-Oxadiazoles among marketed or clinically-studied small molecules.

1,3,4-Oxadiazoles are widely applied in the development of advanced materials, such as electroluminescent and electron-transport materials.^1,2^ In other cases, they have exhibited a variety of biological effects such as antiviral,^3^ antitumor^4^ and anti-inflammatory^5^ activities (Fig. 1). As a design element in medicinal chemistry, 1,3,4-oxadiazoles are deployed for several purposes.^6,7^ For example, this heterocycle can modify small molecule physicochemical and pharmacokinetic profiles due to its use as an aromatic ring spacer with relatively low lipophilicity. It can also act as a bioisosteric hydrogen bond acceptor for carbonyl compounds such as ketones, esters, amides and carbamates while being resistant toward metabolism by hydrolytic esterase and peptidase enzymes.

**Scheme 1:**
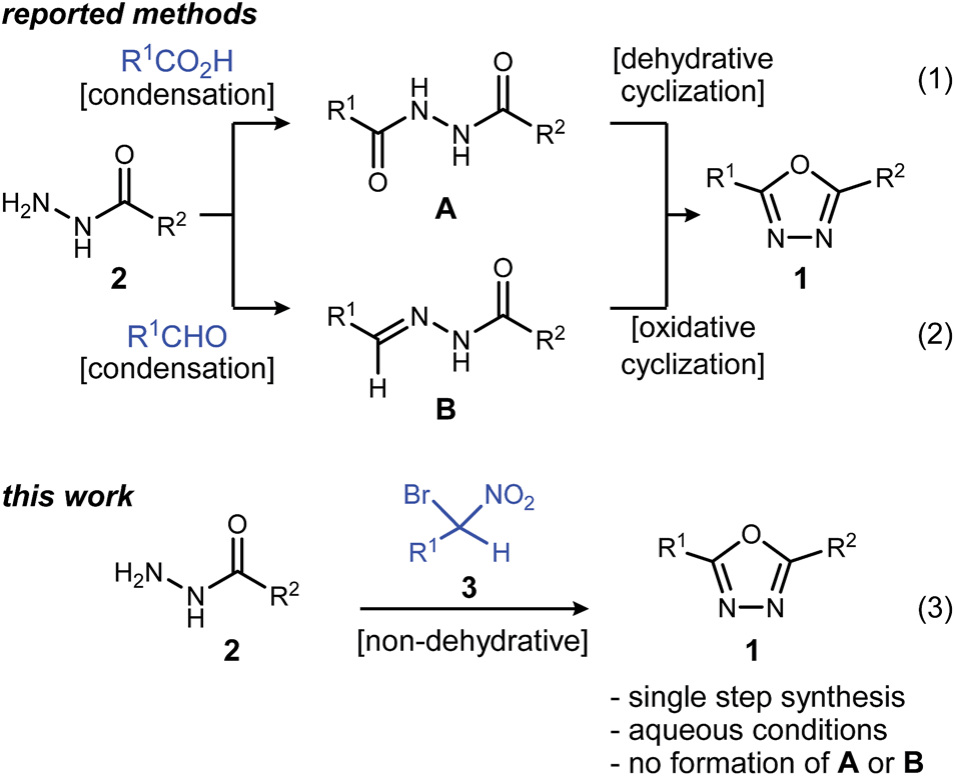
Approaches to oxadiazole synthesis from monoacyl hydrazide.

These attractive characteristics have driven the development of reactions to construct 1,3,4-oxadiazoles.^8,9^ Among these, two approaches starting from monoacyl hydrazide (**2**) have been most thoroughly investigated (Scheme 1). First, the condensation with carboxylic acids followed by dehydrative cyclization of the resulting diacyl hydrazide intermediate (**A**, Scheme 1, eqn (1)) is most extensively explored.^6,8,10^ In addition to the need to form an unsymmetrical diacyl hydrazide, this methodology typically requires harsh reaction conditions (elevated temperature, strong acidic conditions), which can limit the substrate scope. The development of unique electrophilic dehydrating reagents has improved formation of the 1,3,4-oxadiazole core at ambient temperature,^6,10^ though limitations remain among the functional groups tolerated. Oxidative cyclization of acyl hydrazones (**B**, Scheme 1, eqn (2)) prepared from monoacyl hydrazides by condensation with aldehydes complement dehydrative methods, however, the reported examples of this type are currently narrower in scope.^8^

Aside from their inherent value in therapeutic development, 1,3,4-oxadiazoles attracted our attention initially by the hypothesis that Umpolung Amide Synthesis (UmAS)^11,12^ might be used to prepare unsymmetrical diacyl hydrazides (**A**). In this approach, the amide bond would result from an α-bromo nitroalkane (**3**) and monoacyl hydrazide (**2**) using a halonium reagent promoter (Scheme 1, eqn (3)). Unexpectedly, the 1,3,4-oxadiazole, rather than **A**, was formed directly from α-bromo nitroalkane and monoacyl hydrazide. Evidence collected in this study suggests that the initially targeted diacyl hydrazide (**A**) is not an intermediate. Not only is the oxadiazole prepared in fewer steps than existing methodology, but preparative details are endowed with the advantages of UmAS: a mild, non-dehydrative preparation that avoids highly electrophilic reagents and high temperatures that would otherwise limit chemoselectivity. Furthermore, the increasing availability of α-bromo nitroalkanes (**3**) in enantioenriched form^13–16^ further broadens the diversity of oxadiazoles now accessible.

## Results and discussion

We initially examined the reaction between α-bromo nitroalkane (**3a**) and ethyl carbazate (**2a**) (Table 1). First varying the solvent, examination of typical UmAS conditions^11^ (*N*-iodosuccinimide (NIS), base) led to mixed results (Table 1, entries 1–5). In low polarity solvents such as dichloromethane and toluene, no reaction occurred (Table 1, entries 1 and 2), whereas the reaction did proceed in polar solvents (Table 1, entries 3–5). Interestingly, oxadiazole (**4a**) was formed to the exclusion of diacyl hydrazide (**5**) (*cf.* **A**, Scheme 1), the expected product by an UmAS reaction. Among the polar solvents examined, DME gave the highest yield (26%) of the desired 1,3,4-oxadiazole product (**4a**), albeit with incomplete conversion (Table 1, entry 5). The semiaqueous conditions and mild temperature are rather unique among alternative cyclization approaches to oxadiazoles, so the investigation continued with the goal to optimize the base for this reaction (Table 1, entries 6–9). Potassium hydroxide gave a similar result to potassium carbonate (Table 1, entry 6), but weaker bases such as sodium bicarbonate and Hünig's base dramatically decreased the conversion (Table 1, entries 7 and 8). Stronger soluble bases such as DBU caused decomposition of α-bromo nitroalkane (**3a**) (Table 1, entry 9).
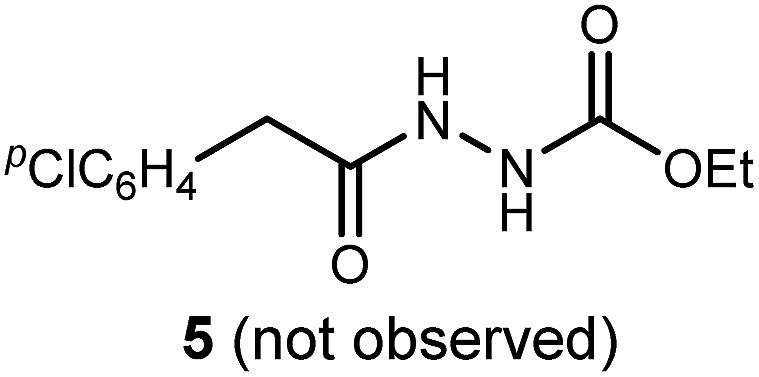


**Table 1:** α-Bromo nitroalkane couplings with an acyl hydrazide: reaction discovery and development

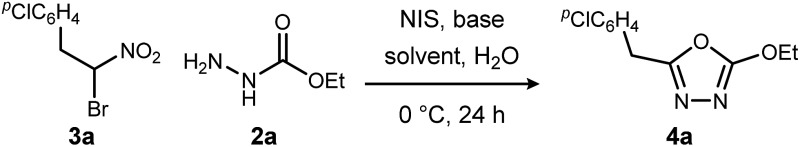				
Entry^*a*^	Solvent	Base	Conversion^*b*^ of **3a** (%)	Yield^*b*^ (%)
1	CH_2_Cl_2_	K_2_CO_3_	Trace	—
2	Toluene	K_2_CO_3_	Trace	—
3	EtOAc	K_2_CO_3_	30	14
4	EtOH	K_2_CO_3_	55	14
5	DME	K_2_CO_3_	72	29
6	DME	KOH	59	24
7	DME	NaHCO_3_	12	—
8	DME	^i^Pr_2_NEt	25	11
9	DME	DBU	100	—

^*a*^All reactions were conducted using **3a** (0.10 mmol, 0.10 M), **2a** (1.2 equiv.), NIS (1.0 equiv.), base (2.0 equiv.) and H_2_O (5.0 equiv.) at 0 °C.

^*b*^Determined by ^1^H NMR of crude reaction mixture using dibromomethane as a standard.

**Scheme 2:**
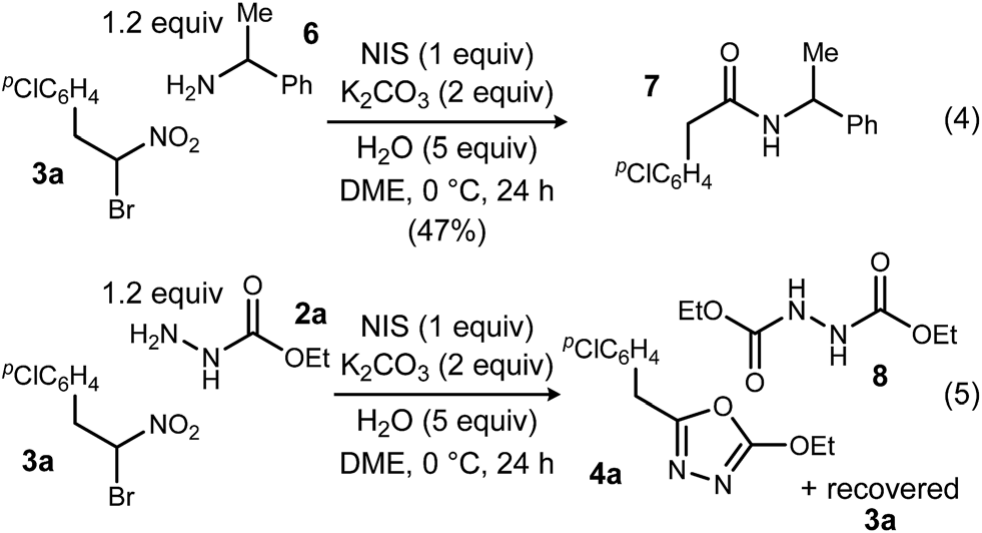
Comparative analysis of UmAS conditions using monoacyl hydrazide and amine acceptors.

To probe possible reasons for low conversions noted in Table 1, α-methylbenzylamine (**6**) and ethyl carbazate (**2a**) were directly compared in their reaction with α-bromo nitroalkane (Scheme 2). When α-methylbenzylamine (**6**) was used, starting material was fully converted to the amide (**7**), as expected (Scheme 2, eqn (4)). The reaction with ethyl carbazate (**2a**) led to two key observations. First, diethyl hydrazinedicarboxylate (**8**) was isolated as a byproduct, and second, conversion of **3a** was incomplete (Scheme 2, eqn (5)). This byproduct formation suggested that either an iodohydrazine or diazene species,^17^ both plausible key intermediates for oxadiazole formation, was over-oxidized to an acyl diazonium species that could react with unreacted ethyl carbazate to give diethyl hydrazinedicarboxylate.^18^ This led to the hypothesis that the lifetime of the activated hydrazine species might be modulated by the halonium source to improve the yield of **4a** (Table 2).

**Table 2:** Evaluation of halonium source for direct oxadiazole synthesis

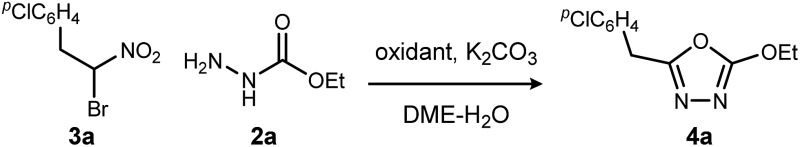			
Entry^*a*^	Oxidant (equiv.)	Conversion^*c*^ of **3a** (%)	Yield^*c*^ (%)
1	NBS (1)	66	8
2	NCS (1)	100	19
3	I_2_ (1)	58	34
4	FeCl_3_ (0.3)/O_2_ (balloon)	54	Trace
5	Cu(OAc)_2_ (2)	69	5
6	KI (2)/^*t*^BuOOH (1)	100	42
7	KI (2)/MnO_2_ (5)	100	39
8	KI (2)/UHP (1)	92	63
**9** ^*b*^	**KI (2)/UHP (1)**	**100**	**70** (70)^*d*^
10	UHP (1)	100	8
11	KI (2), no oxidant	96	13

^*a*^All reactions except for entry 9 were conducted using **3a** (0.10 mmol, 0.10 M), **2a** (0.12 mmol, 1.2 equiv.), oxidant, K_2_CO_3_ (0.20 mmol, 2.0 equiv.), H_2_O (0.50 mmol, 5.0 equiv.) at 0 °C.

^*b*^Reaction was conducted by adding UHP solution (0.20 mL, 0.50 M solution in 4 : 1 DME–H_2_O) over 2 h to the mixture of **3a** (0.10 mmol), **2a** (1.2 equiv.), K_2_CO_3_ (2.0 equiv.) and KI (2.0 equiv.) in DME (1 mL) at rt.

^*c*^Determined by ^1^H NMR of the crude reaction mixture using dibromomethane as a standard.

^*d*^Isolated yield.

Replacing NIS with NBS (*N*-bromosuccinimide) or NCS (*N*-chlorosuccinimide) considerably decreased the yield of 1,3,4-oxadiazole (Table 2, entries 1 and 2). On the other hand, iodine gave a result similar to NIS (Table 2, entry 3). From these observations, an iodonium reagent is superior to other haloniums in this reaction. Transition metal oxidants were also examined, however, only decomposition products were noted along with poor conversion (Table 2, entries 4 and 5). The *in situ* generation of iodonium from iodide and oxidant (Table 2, entries 6–8) was then examined. This approach was considerably more effective, and the best result was obtained when urea-hydrogen peroxide (UHP)^18^ was used (Table 2, entry 8). Slow addition of urea-hydrogen peroxide further improved the desired oxadiazole yield to 70% (Table 2, entry 9). This KI-UHP system is advantageous because the stoichiometric co-products are easily removed by a simple extraction protocol that streamlines purification of the desired product. Appropriate control experiments confirmed the synergistic action of KI and UHP (Table 2, entries 10 and 11).^19^ Formation of diethyl hydrazinedicarboxylate **8** was minimized when using the optimized conditions, suggesting that acyl hydrazide **2a** was chaperoned efficiently through the oxadiazole-forming pathway.

Exploration of the substrate scope was pursued with these optimized conditions, and a broad range of acyl hydrazides performed well (Table 3): alkoxycarbonyl (Table 3, entry 1), aminocarbonyl (Table 3, entries 2 and 3), substituted phenyl carbonyl (Table 3, entries 4–6), heteroaromatic carbonyl (Table 3, entries 7–9) and alkyl carbonyl hydrazides (Table 3, entries 10–12). These choices provided a range of carbonyl electronic character. In the study of aryl carbonyl hydrazides, we established that electron-rich aromatics are tolerated despite their potential to react with the oxidant (Table 3, entries 5, 7 and 8). An unprotected hydroxyl that might not be tolerated under dehydrative oxadiazole formation conditions, was not problematic here (Table 3, entry 12).

**Table 3:** Substrate scope for oxadiazole synthesis from α-bromo nitroalkanes and acyl hydrazides

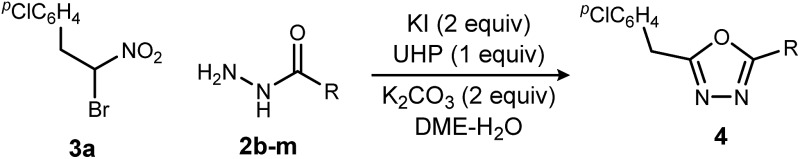			
Entry^*a*^	R	Product	Yield^*b*^ (%)
1	O-*t*-Bu	**4b**	80
2	NMe_2_	**4c**	76
3	NH-*c*-Hex	**4d**	81
4	Ph	**4e**	62
5	^*p*^(MeO)C_6_H_4_	**4f**	64
6	^*p*^MeC_6_H_4_	**4g**	70
7	2-Thienyl	**4h**	60
8	2-Furyl	**4i**	61
9	2-Pyridyl	**4j**	54
10	*cyclo*-Pr	**4k**	81
11	iso-Pr	**4l**	74
12	C(OH)Me_2_	**4m**	60

^*a*^Reaction was conducted by adding UHP solution (1.0 equiv., 0.50 M solution in 4 : 1 DME–H_2_O) over 2 h to the mixture of **3a** (0.15–0.50 mmol), **2** (1.2 equiv.), K_2_CO_3_ (2.0 equiv.) and KI (2.0 equiv.) in DME (1 mL) at rt.

^*b*^Isolated yield.

This reaction is also applicable to various α-bromo nitroalkanes regardless of the electron density of α-position of nitro group (Table 4). Introduction of functionalities such as an electron rich aromatic group (**9**), base-sensitive nitrile (**10**), and ester (**13**) further broadens the scope of oxadiazoles prepared by this method.

**Table 4:** Oxadiazole synthesis: additional scope^*a*^ ^*b*^

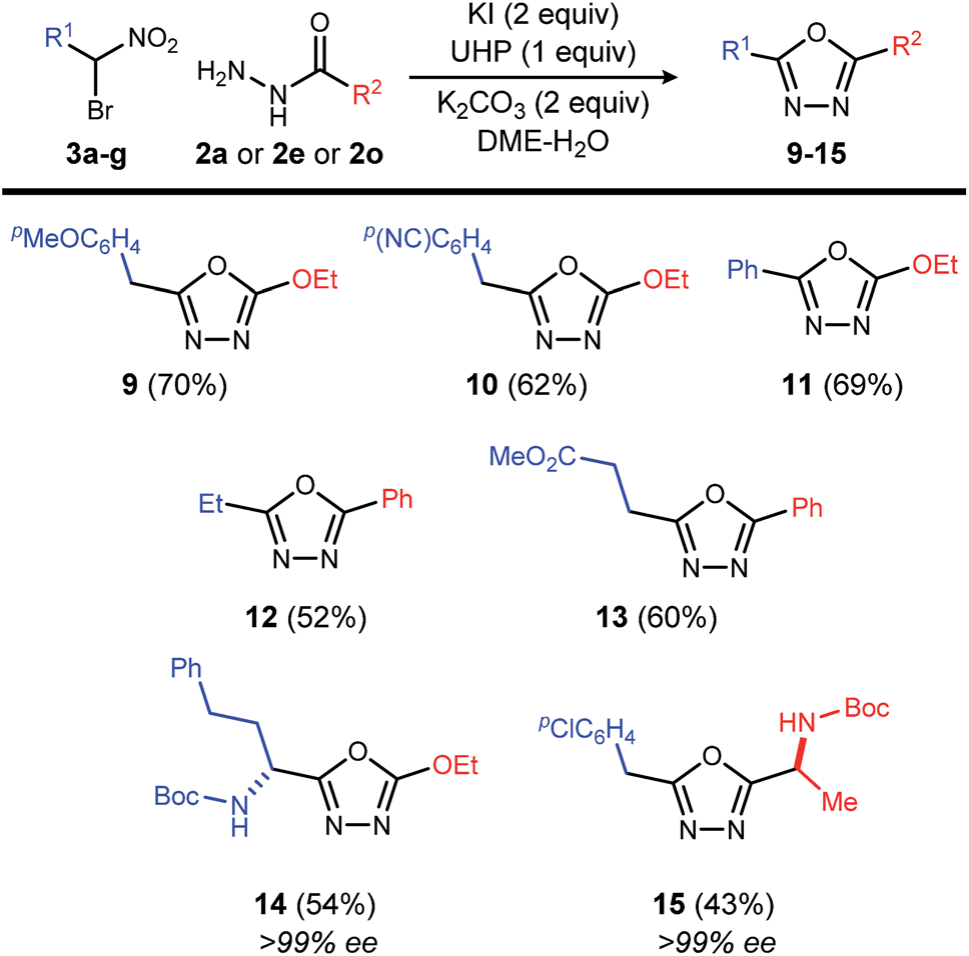

^*a*^Reaction was conducted by adding UHP solution (1.0 equiv., 0.50 M solution in 4 : 1 DME–H_2_O) over 2 h to the mixture of **3** (0.20–1.0 mmol), **2** (1.2 equiv.), K_2_CO_3_ (2.0 equiv.) and KI (2.0 equiv.) in DME (1 mL) at rt.

^*b*^Isolated yield in parenthesis.

Chiral, nonracemic α-bromo nitroalkanes are increasingly available in as little as a single step using enantioselective catalysis and bromonitromethane reagent.^13–16^ The reaction of β-amino-α-bromo nitroalkane (**3g**), and α-amino acid-derived acyl hydrazide (**2o**), provide the enantiopure oxadiazole products (**14** and **15**), demonstrating that no racemization of either chiral α-bromo nitroalkane or amino acid hyrazide occurred during the oxadiazole formation.

**Scheme 3:**
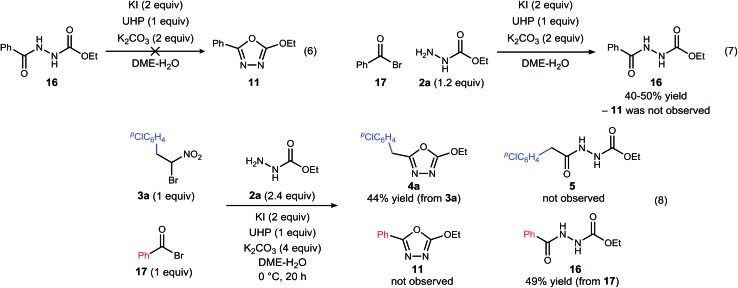
Experiments designed to probe the intermediacy of a diacyl hydrazide (**5**, **16**), and the behavior of an active ester (**17**).

We returned to the question of mechanism since our initial findings noted the conspicuous absence of diacyl hydrazide **5**, the entity originally targeted by UmAS with an acyl hydrazide. In our prior work with amines and a broad range of α-bromo nitroalkanes bearing β-stereogenic centers, we have advanced the hypothesis that the key carbon–nitrogen bond forming step involves a negatively polarized carbon/positively polarized nitrogen pair. Our work, in its entirety, is consistent with this picture, but the absence of the expected diacyl hydrazide provided a new opportunity to investigate the mechanism (Scheme 3).

The possibility that diacyl hydrazide is an intermediate in a stepwise oxadiazole synthesis was probed by its preparation and exposure to the reaction conditions. The diacyl hydrazide (**16**) did not convert to oxadiazole (**11**) (Scheme 3, eqn (6)). The feasibility of acyl bromide formation from α-bromo nitroalkane and its ability to function as an active ester under the reaction conditions was also evaluated (Scheme 3, eqn (7)). Only diacyl hydrazide (**16**) was produced and no evidence for oxadiazole formation was obtained. In a final attempt to illustrate orthogonal reactivity of the α-bromo nitroalkane and active ester, a combination reaction of acyl bromide (**17**) and α-bromo nitroalkane (**3a**) was performed with monoacyl hydrazide (**2a**) under the typical reaction conditions (Scheme 3, eqn (8)). No evidence for cross-over product formation was observed: only diacyl hydrazide (**16**) was formed from acyl bromide (**17**), and only oxadiazole (**4a**) was obtained from α-bromo nitroalkane (**3a**).

**Scheme 4:**
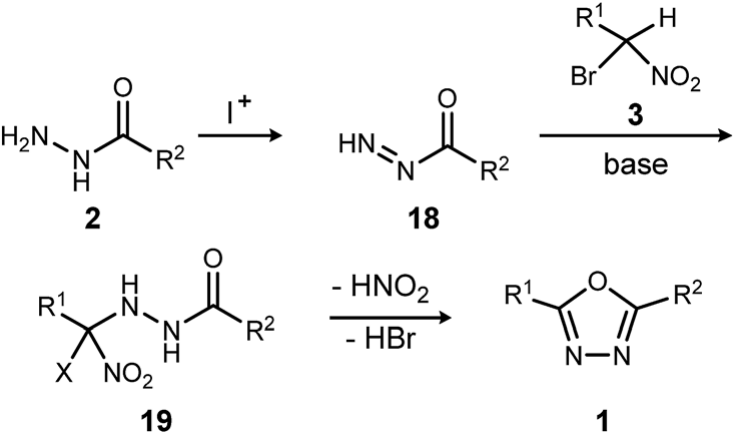
Outline of key bond-forming steps for oxadiazole synthesis from acyl hydrazides and α-bromo nitroalkanes.

The reaction mechanism consistent with these observations is shown in Scheme 4. It is clear that neither a diacyl hydrazide nor an active ester species is an intermediate in the conversion of α-bromo nitroalkane to oxadiazole. Instead, the reaction of a nucleophilic nitronate and electrophilic hydrazine nitrogen species is supported, and a mechanism that avoids a diacyl hydrazide is hypothesized. The acyl diazene (**18**)^17^ forms by the oxidative action of electrophilic iodine to monoacyl hydrazide (**2**), perhaps through a terminal *N*-iodo acyl hydrazide and subsequent base-mediated elimination. Following nitronate addition to this electrophilic nitrogen species, the putative α-hydrazino-α-bromo nitroalkane intermediate (**19**) so-formed undergoes cyclization to an oxadiazoline intermediate, and then oxadiazole. The elementary steps from **19** to **1** are not detailed, but this intramolecular cyclization may be triggered by nitro-nitrite isomerization as previously proposed,^12^ followed by cyclization and an elimination of the elements of hydrogen bromide to produce the oxadiazole. It is significant to note that the mechanistically-directed experiments described here further contraindicate an active ester intermediate.^20^

## Conclusion

In summary, a new protocol to synthesize 1,3,4-oxadiazole from α-bromo nitroalkanes and monoacyl hydrazides has been discovered. The mildly basic reaction conditions, tolerance toward water, and broad substrate scope are complementary to existing oxadiazole synthesis methods. *In situ* formation of an electrophilic nitrogen species provides entry to the UmAS amidation pathway, but the intermediate is rapidly converted to oxadiazole rather than diacyl hydrazide. This mechanistic dichotomy from a typical active ester intermediate results in mild reaction conditions, rather than more forcing dehydrative conditions typical of diacyl hydrazide-based methods, and expands the number of readily available oxadiazoles.
