# Supplementary material for: A convergent synthesis of 1,3,4-oxadiazoles from acyl hydrazides under semiaqueous conditions
Source: Chem Sci. 2017 Feb 23;8(4):3187–91. doi: 10.1039/c7sc00195a (PMC5414388; doi:10.1039/c7sc00195a)
Supplement: Supplementary file 1 [file SC-008-C7SC00195A-s001.pdf]

Electronic Supplementary Material (ESI) for Chemical Science.
This journal is © The Royal Society of Chemistry 2017

A Convergent Synthesis of 1,3,4-Oxadiazoles from Acyl Hydrazides under Semiaqueous Conditions

Kazuyuki Tokumaru* and Jeffrey N. Johnston*

Department of Chemistry and Vanderbilt Institute of Chemical Biology,
Vanderbilt University, Nashville, Tennessee 37235

SI-I-X

Experimental section....2
Preparation of α-bromo nitroalkanes 2
General procedure for the synthesis of α-bromo nitroalkane from α-bromo nitroalkene....2
1-(2-Bromo-2-nitroethyl)-4-chlorobenzene (3a) 2
1-(2-Bromo-2-nitroethyl)-4-methoxybenzene (3b) 3
4-(2-Bromo-2-nitroethyl)benzonitrile (3c). 3
General procedure for the synthesis of 2,5-disubstituted 1,3,4-oxadiazole....3
2-(4-Chlorobenzyl)-5-ethoxy-1,3,4-oxadiazole (4a) 3
2-(*tert*-Butoxy)-5-(4-chlorobenzyl)-1,3,4-oxadiazole (4b)....4
5-(4-Chlorobenzyl)-*N*,*N*-dimethyl-1,3,4-oxadiazol-2-amine (4c). 4
5-(4-Chlorobenzyl)-*N*-cyclohexyl-1,3,4-oxadiazol-2-amine (4d) 4
2-(4-Chlorobenzyl)-5-phenyl-1,3,4-oxadiazole (4e) 4
2-(4-Chlorobenzyl)-5-(4-methoxyphenyl)-1,3,4-oxadiazole (4f)....5
2-(4-Chlorobenzyl)-5-(*o*-tolyl)-1,3,4-oxadiazole (4g)....5
2-(4-Chlorobenzyl)-5-(thiophen-2-yl)-1,3,4-oxadiazole (4h)....5
2-(4-Chlorobenzyl)-5-(furan-2-yl)-1,3,4-oxadiazole (4i). 6
2-(4-Chlorobenzyl)-5-(pyridin-2-yl)-1,3,4-oxadiazole (4j). 6
2-(4-Chlorobenzyl)-5-cyclopropyl-1,3,4-oxadiazole (4k)....6
2-(4-Chlorobenzyl)-5-isopropyl-1,3,4-oxadiazole (4l)....6
2-(5-(4-Chlorobenzyl)-1,3,4-oxadiazol-2-yl)propan-2-ol (4m)....7
2-Ethoxy-5-(4-methoxybenzyl)-1,3,4-oxadiazole (9)....7
4-((5-Ethoxy-1,3,4-oxadiazol-2-yl)methyl)benzonitrile (10). 7
2-Ethoxy-5-phenyl-1,3,4-oxadiazole (11). 8
2-Ethyl-5-phenyl-1,3,4-oxadiazole (12) 8
Methyl 3-(5-phenyl-1,3,4-oxadiazol-2-yl)propanoate (13) 8
tert-Butyl (*R*)-(1-(5-ethoxy-1,3,4-oxadiazol-2-yl)-3-phenylpropyl)carbamate (14). 9
tert-Butyl (*S*)-(1-(5-(4-chlorobenzyl)-1,3,4-oxadiazol-2-yl)ethyl)carbamate (15)....9
Experiment in Scheme 2 9
Synthesis of 2-(4-chlorophenyl)-*N*-(1-phenylethyl)acetamide (7) (eq 4). 9
Experiments in Scheme 3....10
Exposure of diacyl hydrazide 16 to the reaction conditions (eq 6)....10
Reaction of acyl bromide 17 and acyl hydrazide 2a under the reaction conditions (eq 7) 10
Crossover experiment using α-bromo nitroalkane 3a and acyl bromide 17 (eq 8)....10

Experimental section

All reagents and solvents were commercial grade and purified prior to use when necessary. *N*-Iodosuccinimide was recrystallized from dioxane/CCl_4 and *N*-bromosuccinimide was recrystallized from water prior to use. Flash column chromatography was performed using Sorbent Technologies 40-63 mm, pore size 60 Å silica gel with solvent systems indicated. Analytical thin layer column chromatography was performed using Sorbent Technologies 250 mm glass-backed UV254 silica gel plates, and were visualized by fluorescence upon 250 nm radiation and/or the by use of ceric ammonium molybdate, ninhydrin, or potassium permanganate. Solvent removal was effected by rotary evaporation under vacuum (~ 25-40 mm Hg). All extracts were dried with sodium sulfate unless otherwise noted.

Nuclear magnetic resonance spectra (NMR) were acquired on a Bruker AV-400 (400 MHz), Bruker DRX-500 (500 MHz), or Bruker AV II-600 (600 MHz) instrument. Chemical shifts are measured relative to residual solvent peaks as an internal standard ($CHCl_3$ at 7.26 ppm in ^{1}H NMR, 77.16 ppm in ^{13}C NMR), unless otherwise specified. Mass spectra were recorded on a Thermo Electron Corporation MAT 95XP-Trap mass spectrometer by use of the ionization method noted by the Indiana University Mass Spectrometry Facility. IR spectra were recorded on a Nicolet Avatar 360 spectrophotometer and are reported in wavenumbers (cm^{-1}) as neat films on a NaCl plate (transmission). Melting points were obtained using an OptiMelt automated melting point system available (Stanford Research Systems). Optical rotations were measured on a Perkin Elmer-341 polarimeter.

Preparation of α-bromo nitroalkanes

Known α-bromo nitroalkanes were prepared according to the reported procedures[5,6,9,10]. Other α-bromo nitroalkanes (**3a**, **3b** and **3c**) were synthesized from the corresponding α-bromo nitroalkenes[2,3,4] by reduction using sodium borohydride.[1]

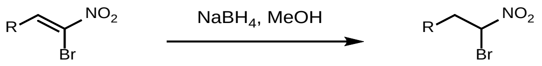

General procedure for the synthesis of α-bromo nitroalkane from α-bromo nitroalkene: To a solution of α-bromo nitroalkene (11 mmol) in methanol (50 mL) was added sodium borohydride (5.7 mmol, 0.50 equiv) in portions at 0 °C. After it was stirred for 10 min, the reaction mixture was carefully poured into 1 M hydrochloric acid (100 mL) and extracted with ethyl acetate. The combined organic layers were washed with brine, dried over sodium sulfate and concentrated *in vacuo*. The residue was purified by flash column chromatography to give pure α-bromo nitroalkane.

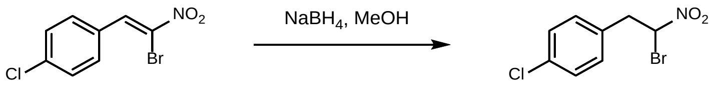

1-(2-Bromo-2-nitroethyl)-4-chlorobenzene (3a). Following the general procedure, the α-bromo nitroalkene[2] (3.0 g, 11 mmol) provided the α-bromo nitroalkane **3a** after flash column chromatography (silica gel, 5% ethyl acetate in hexanes) as a pale yellow oil (2.1 g, 70% yield). R_f = 0.40 (10% ethyl acetate/hexanes); IR (film) 3025, 2909, 1564, 1352, 1096, 811 cm^{-1}; ^{1}H NMR (400 MHz, $CDCl_3$) δ 7.34-7.31 (m, 2H), 7.16-7.13 (m, 2H), 6.01 (dd, *J* = 8.1, 6.1 Hz, 1H), 3.72 (dd, *J* = 14.6, 8.1 Hz, 1H), 3.49 (dd, *J* = 14.6, 6.1 Hz, 1H); ^{13}C NMR (100 MHz, $CDCl_3$) ppm 134.4, 131.7, 130.6, 129.3, 78.8, 42.7; HRMS (CI): Exact mass calcd for $C_8H_7BrClNO_2$ $[M]^+$ 262.9343, found 262.9345.

[1] Dauzonne, D.; Royer, R. *Synthesis* **1988**, 339.

[2] Pleshchev, M. I.; Das Gupta, N. V.; Kuznetsov, V. V.; Fedyanin, I. V.; Kachala, V. V.; Makhova, N. N. *Tetrahedron* **2015**, *71*, 9012.

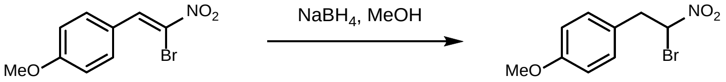

1-(2-Bromo-2-nitroethyl)-4-methoxybenzene (3b). Following the general procedure, the α-bromo nitroalkene[3] (4.0 g, 16 mmol) provided the α-bromo nitroalkane **3b** after flash column chromatography (silica gel, 10% ethyl acetate in hexanes) as a pale yellow oil (1.9 g, 47% yield). R_f = 0.30 (10% ethyl acetate/hexanes); IR (film) 3012, 2958, 2839, 1566, 1516, 1354, 1300, 1253, 1034, 823 cm^{-1}; ^1H NMR (400 MHz, $CDCl_3$) δ 7.12 (d, *J* = 8.6 Hz, 2H), 6.86 (d, *J* = 8.6 Hz, 2H), 5.99 (dd, *J* = 8.3, 5.9 Hz, 1H), 3.79 (s, 3H), 3.68 (dd, *J* = 14.7, 8.3 Hz, 1H), 3.44 (dd, *J* = 14.7, 5.9 Hz, 1H); ^{13}C NMR (100 MHz, $CDCl_3$) ppm 159.6, 130.4, 125.3, 114.6, 79.5, 55.3, 42.9; HRMS (CI): Exact mass calcd for $C_9H_{10}BrNO_3$ $[M]^+$ 258.9839, found 258.9829.

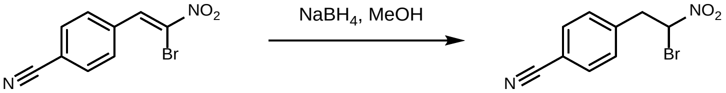

4-(2-Bromo-2-nitroethyl)benzonitrile (3c). Following the general procedure, the α-bromo nitroalkene[4] (1.5 g, 5.9 mmol) provided the α-bromo nitroalkane **3c** after flash column chromatography (silica gel, 15% ethyl acetate in hexanes) as a colorless yellow oil (460 mg, 30% yield). R_f = 0.13 (10% ethyl acetate/hexanes); IR (film) 3018, 2230, 1563, 1354, 858 cm^{-1}; ^1H NMR (400 MHz, $CDCl_3$) δ 7.67-7.65 (m, 2H), 7.36-7.34 (m, 2H), 6.07 (dd, *J* = 7.8, 6.4 Hz, 1H), 3.82 (dd, *J* = 14.6, 7.8 Hz, 1H), 3.59 (dd, *J* = 14.6, 6.4 Hz, 1H); ^{13}C NMR (100 MHz, $CDCl_3$) ppm 138.4, 132.9, 130.2, 118.2, 112.7, 78.2, 43.2; HRMS (CI): Exact mass calcd for $C_9H_7BrN_2O_2$ $[M]^+$ 254.9764, found 254.9762.

General procedure for the synthesis of 2,5-disubstituted 1,3,4-oxadiazole: To a vigorously stirred mixture of α-bromo nitroalkane (0.50 mmol), monoacyl hydrazide (0.60 mmol, 1.2 equiv), potassium iodide (1.0 mmol, 2.0 equiv) and potassium carbonate (1.0 mmol, 2.0 equiv) in 1,2-dimethoxyethane (5.0 mL) was added a solution of urea-hydrogen peroxide in 4:1 1,2-dimethoxyethane-water (0.50 M solution, 1.0 mL, 0.50 mmol, 1.0 equiv) over 2 h by syringe pump at room temperature. After the addition was complete, the mixture was stirred for additional 4 h. Aqueous sodium thiosulfate was then added and the mixture was extracted with ethyl acetate. The combined organic layers were washed with brine, dried over sodium sulfate and concentrated *in vacuo*. The residue was purified by flash column chromatography to give pure 2,5-disubstituted 1,3,4-oxadiazole.

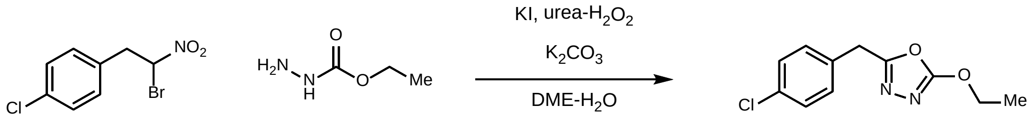

2-(4-Chlorobenzyl)-5-ethoxy-1,3,4-oxadiazole (4a). Following the general procedure, the α-bromo nitroalkane **3a** (26 mg, 0.10 mmol) and the monoacyl hydrazide **2a** (13 mg, 0.12 mmol) provided the 2,5-disubstituted 1,3,4-oxadiazole **4a** after flash column chromatography (silica gel, 25-50% ethyl acetate in hexanes) as a colorless solid (17 mg, 70% yield). Mp 53-54 °C (recrystallized from ethyl acetate/hexanes); R_f = 0.30 (33% ethyl acetate/hexanes); IR (film) 2989, 1633, 1568, 1320, 1024 cm^{-1}; ^1H NMR (400 MHz, $CDCl_3$) δ 7.33-7.29 (m, 2H), 7.25-7.22 (m, 2H), 4.50 (q, *J* = 7.1 Hz, 2H), 4.02 (s, 2H), 1.45 (t, *J* = 7.1 Hz, 3H); ^{13}C NMR (100 MHz, $CDCl_3$) ppm 166.1, 160.6, 133.6, 132.3, 130.3, 129.1, 69.1, 31.7, 14.3; HRMS (ESI): Exact mass calcd for $C_{11}H_{12}ClN_2O_2$ $[M+H]^+$ 239.0587, found 239.0580.

---

[3] Greger, J. G.; Yoon-Miller, S. J. P.; Bechtold, N. R.; Flewelling, S. A.; MacDonald, J. P.; Downey, C. R.; Cohen, E. A.; Pelkey, E. T. *J. Org. Chem.* **2011**, *76*, 8203.
[4] Vecchi, A.; Melone, G. *J. Org. Chem.* **1957**, *22*, 1636.

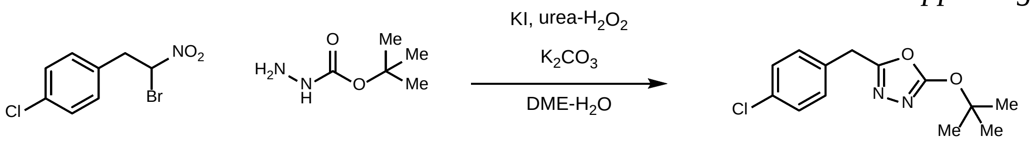

2-(*tert*-Butoxy)-5-(4-chlorobenzyl)-1,3,4-oxadiazole (4b). Following the general procedure, the α-bromo nitroalkane **3a** (130 mg, 0.50 mmol) and the monoacyl hydrazide **2b** (79 mg, 0.60 mmol) provided the 2,5-disubstituted 1,3,4-oxadiazole **4b** after flash column chromatography (silica gel, 25-50% ethyl acetate in hexanes) as a colorless oil (106 mg, 80% yield). R_f = 0.50 (33% ethyl acetate/hexanes); IR (film) 2982, 1616, 1566, 1491, 1378, 1243, 1159, 1093, 870 cm^{-1}; ^{1}H NMR (400 MHz, $CDCl_3$) δ 7.32-7.29 (m, 2H), 7.25-7.22 (m, 2H), 4.01 (s, 2H), 1.59 (s, 9H); ^{13}C NMR (100 MHz, $CDCl_3$) ppm 164.3, 160.1, 133.5, 132.5, 130.3, 129.1, 87.3, 31.7, 27.6; HRMS (CI): Exact mass calcd for $C_{13}H_{15}ClN_2O_2$ $[M]^+$ 266.0817, found 266.0804.

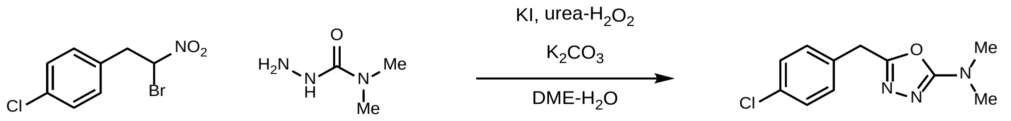

5-(4-Chlorobenzyl)-*N*,*N*-dimethyl-1,3,4-oxadiazol-2-amine (4c). Following the general procedure, the α-bromo nitroalkane **3a** (130 mg, 0.50 mmol) and the monoacyl hydrazide **2c** (62 mg, 0.60 mmol) provided the 2,5-disubstituted 1,3,4-oxadiazole **4c** after flash column chromatography (silica gel, 50-100% ethyl acetate in hexanes) as a colorless oil (90 mg, 76% yield). R_f = 0.50 (33% ethyl acetate/hexanes); IR (film) 2930, 1642, 1583, 1489, 1431, 1246, 1091, 914 cm^{-1}; ^{1}H NMR (400 MHz, $CDCl_3$) δ 7.31-7.28 (m, 2H), 7.24- 7.20 (m, 2H), 4.01 (s, 2H), 3.01 (s, 6H); ^{13}C NMR (100 MHz, $CDCl_3$) ppm 165.3, 158.7, 133.2, 133.1, 130.1, 128.9, 38.1, 31.4; HRMS (ESI): Exact mass calcd for $C_{11}H_{13}ClN_3O$ $[M+H]^+$ 238.0747, found 238.0738.

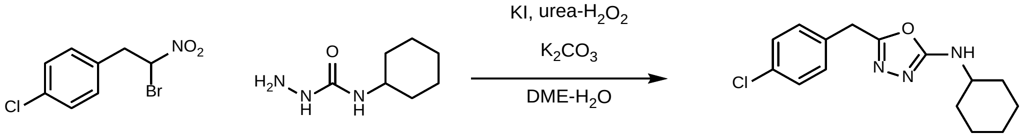

5-(4-Chlorobenzyl)-*N*-cyclohexyl-1,3,4-oxadiazol-2-amine (4d). Following the general procedure, the α-bromo nitroalkane **3a** (130 mg, 0.50 mmol) and the monoacyl hydrazide **2d** (94 mg, 0.60 mmol) provided the 2,5-disubstituted 1,3,4-oxadiazole **4d** after flash column chromatography (silica gel, 20-100% ethyl acetate in hexanes) as a colorless solid (118 mg, 81% yield). Mp 148-150 °C (recrystallized from ethyl acetate/hexanes) R_f = 0.17 (50% ethyl acetate/hexanes); IR (film) 3196, 3023, 2931, 2856, 1636, 1580, 913 cm^{-1}; ^{1}H NMR (400 MHz, $CDCl_3$) δ 7.32-7.28 (m, 2H), 7.24-7.20 (m, 2H), 4.37 (d, J = 7.8 Hz, 1H), 4.00 (s, 2H), 3.51 (tdt, J = 10.3, 7.8, 3.9 Hz, 1H), 2.08-2.02 (m, 2H), 1.76-1.69 (m, 2H), 1.65-1.58 (m, 1H), 1.43-1.32 (m, 2H), 1.26-1.14 (m, 3H); ^{13}C NMR (100 MHz, $CDCl_3$) ppm 163.3, 158.4, 133.4, 133.1, 130.2, 129.0, 52.6, 33.3, 31.4, 25.5, 24.7; HRMS (ESI): Exact mass calcd for $C_{15}H_{19}ClN_3O$ $[M+H]^+$ 292.1217, found 292.1218.

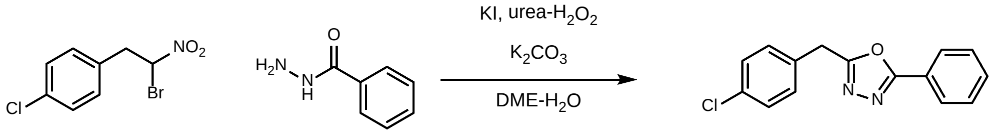

2-(4-Chlorobenzyl)-5-phenyl-1,3,4-oxadiazole (4e). Following the general procedure, the α-bromo nitroalkane **3a** (40 mg, 0.15 mmol) and the monoacyl hydrazide **2e** (24 mg, 0.18 mmol) provided the 2,5-disubstituted 1,3,4-oxadiazole **4e** after flash column chromatography (silica gel, 25% ethyl acetate in hexanes) as a colorless solid (25 mg, 62% yield). Mp 115-117 °C (recrystallized from ethyl acetate/hexanes); R_f = 0.67 (33%

ethyl acetate/hexanes); IR (film) 3062, 1558, 1489, 1087, 1016 cm^{-1}; ^{1}H NMR (400 MHz, $CDCl_3$) δ 8.02-7.99 (m, 2H), 7.55-7.46 (m, 3H), 7.35-7.28 (m, 4H), 4.25 (s, 2H); ^{13}C NMR (100 MHz, $CDCl_3$) ppm 165.4, 164.9, 133.7, 132.4, 131.9, 130.3, 129.2, 129.1, 127.0, 123.9, 31.4; HRMS (CI): Exact mass calcd for $C_{15}H_{12}ClN_2O$ $[M+H]^+$ 271.0633, found 271.0643.

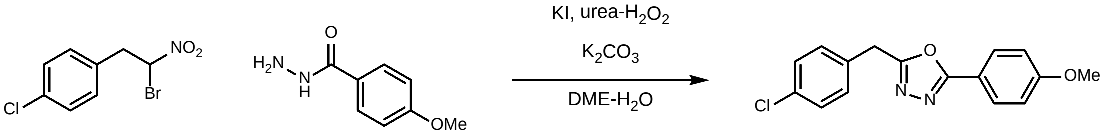

2-(4-Chlorobenzyl)-5-(4-methoxyphenyl)-1,3,4-oxadiazole (4f). Following the general procedure, the α-bromo nitroalkane **3a** (40 mg, 0.15 mmol) and the monoacyl hydrazide **2f** (30 mg, 0.18 mmol) provided the 2,5-disubstituted 1,3,4-oxadiazole **4f** after flash column chromatography (silica gel, 10-50% ethyl acetate in hexanes) as a colorless solid (29 mg, 64% yield). Mp 125-127 °C (recrystallized from ethyl acetate/hexanes); R_f = 0.14 (20% ethyl acetate/hexanes); IR (film) 2947, 1614, 1498, 1423, 1305, 1260, 1175, 1088, 1016, 838 cm^{-1}; ^{1}H NMR (400 MHz, $CDCl_3$) δ 7.95-7.91 (m, 2H), 7.35-7.32 (m, 2H), 7.31-7.28 (m, 2H), 7.00-6.96 (m, 2H), 4.23 (s, 2H), 3.87 (s, 3H); ^{13}C NMR (100 MHz, $CDCl_3$) ppm 165.2, 164.3, 162.4, 133.6, 132.5, 130.2, 129.1, 128.6, 116.3, 114.5, 55.5, 31.3; HRMS (ESI): Exact mass calcd for $C_{16}H_{14}ClN_2O_2$ $[M+H]^+$ 301.0744, found 301.0738.

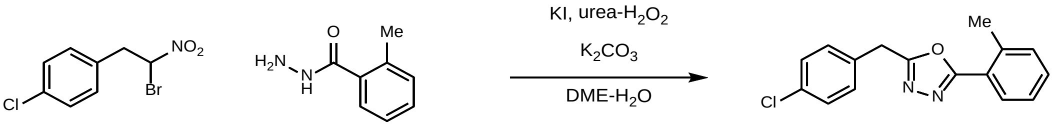

2-(4-Chlorobenzyl)-5-(*o*-tolyl)-1,3,4-oxadiazole (4g). Following the general procedure, the α-bromo nitroalkane **3a** (53 mg, 0.20 mmol) and the monoacyl hydrazide **2g** (36 mg, 0.24 mmol) provided the 2,5-disubstituted 1,3,4-oxadiazole **4g** after flash column chromatography (silica gel, 10-50% ethyl acetate in hexanes) as a colorless solid (40 mg, 70% yield). Mp 98-100 °C (recrystallized from ethyl acetate/hexanes); R_f = 0.27 (20% ethyl acetate/hexanes); IR (film) 3054, 2925, 1551, 1491, 1241, 1090, 1013, 913 cm^{-1}; ^{1}H NMR (400 MHz, $CDCl_3$) δ 7.85 (dd, *J* = 7.6, 1.5 Hz, 1H), 7.40 (td, *J* = 7.6, 1.5 Hz, 1H), 7.35-7.27 (m, 6H), 4.26 (s, 2H), 2.66 (s, 3H); ^{13}C NMR (100 MHz, $CDCl_3$) ppm 165.6, 164.4, 138.5, 133.7, 132.5, 131.8, 131.3, 130.3, 129.2, 129.0, 126.2, 123.0, 31.4, 22.1; HRMS (ESI): Exact mass calcd for $C_{16}H_{14}ClN_2O$ $[M+H]^+$ 285.0795, found 285.0808.

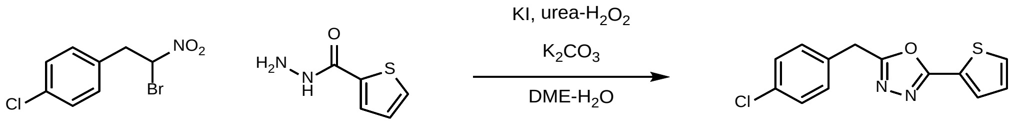

2-(4-Chlorobenzyl)-5-(thiophen-2-yl)-1,3,4-oxadiazole (4h). Following the general procedure, the α-bromo nitroalkane **3a** (53 mg, 0.20 mmol) and the monoacyl hydrazide **2h** (34 mg, 0.24 mmol) provided the 2,5-disubstituted 1,3,4-oxadiazole **4h** after flash column chromatography (silica gel, 25% ethyl acetate in hexanes) as a colorless solid (33 mg, 60% yield). Mp 100-102 °C (recrystallized from ethyl acetate/hexanes); R_f = 0.24 (20% ethyl acetate/hexanes); IR (film) 3097, 1568, 1492, 1422, 1239, 1087, 1005, 847 cm^{-1}; ^{1}H NMR (400 MHz, $CDCl_3$) δ 7.69 (dd, *J* = 3.7, 1.2 Hz, 1H), 7.53 (dd, *J* = 5.0, 1.2 Hz, 1H), 7.35-7.32 (m, 2H), 7.31-7.27 (m, 2H), 7.14 (dd, *J* = 5.0, 3.7 Hz, 1H), 4.23 (s, 2H); ^{13}C NMR (100 MHz, $CDCl_3$) ppm 164.3, 161.6, 133.7, 132.3, 130.3, 130.2, 129.9, 129.2, 128.2, 125.1, 31.3; HRMS (CI): Exact mass calcd for $C_{13}H_{10}ClN_2OS$ $[M+H]^+$ 277.0197, found 277.0200.

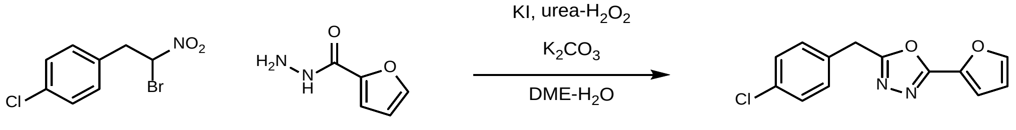

2-(4-Chlorobenzyl)-5-(furan-2-yl)-1,3,4-oxadiazole (4i). Following the general procedure, the α-bromo nitroalkane **3a** (40 mg, 0.15 mmol) and the monoacyl hydrazide **2i** (23 mg, 0.18 mmol) provided the 2,5-disubstituted 1,3,4-oxadiazole **4i** after flash column chromatography (silica gel, 10-50% ethyl acetate in hexanes) as a colorless solid (24 mg, 61% yield). Mp 122-124 °C (recrystallized from ethyl acetate/hexanes); R_f = 0.53 (50% ethyl acetate/hexanes); IR (film) 3130, 1630, 1568, 1528, 1491, 1417, 1163, 1013, 971, 899, 816 cm^{-1}; ^1H NMR (400 MHz, $CDCl_3$) δ 7.61 (dd, *J* = 1.8, 0.8 Hz, 1H), 7.35-7.31 (m, 2H), 7.30-7.27 (m, 2H), 7.12 (dd, *J* = 3.5, 0.8 Hz, 1H), 6.57 (dd, *J* = 3.5, 1.8 Hz, 1H), 4.23 (s, 2H).; ^{13}C NMR (100 MHz, $CDCl_3$) ppm 164.3, 158.2, 145.8, 139.4, 133.8, 132.2, 130.3, 129.2, 114.2, 112.2, 31.2; HRMS (CI): Exact mass calcd for $C_{13}H_{10}ClN_2O_2$ $[M+H]^+$ 261.0431, found 261.0430.

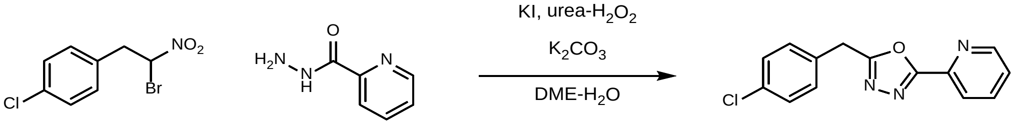

2-(4-Chlorobenzyl)-5-(pyridin-2-yl)-1,3,4-oxadiazole (4j). Following the general procedure, the α-bromo nitroalkane **3a** (40 mg, 0.15 mmol) and the monoacyl hydrazide **2j** (25 mg, 0.18 mmol) provided the 2,5-disubstituted 1,3,4-oxadiazole **4j** after flash column chromatography (silica gel, 20-100% ethyl acetate in hexanes) as a colorless solid (22 mg, 54% yield). Mp 93-95 °C (recrystallized from ethyl acetate/hexanes); R_f = 0.23 (50% ethyl acetate/hexanes); IR (film) 3054, 1560, 1491, 1454, 1094, 1016, 913 cm^{-1}; ^1H NMR (400 MHz, $CDCl_3$) δ 8.76 (ddd, *J* = 4.9, 1.8, 1.1 Hz, 1H), 8.23 (dt, *J* = 7.8, 1.1 Hz, 1H), 7.87 (td, *J* = 7.8, 1.8 Hz, 1H), 7.45 (ddd, *J* = 7.8, 4.9, 1.1 Hz, 1H), 7.32 (s, 4H), 4.30 (s, 2H); ^{13}C NMR (100 MHz, $CDCl_3$) ppm 166.0, 164.5, 150.3, 143.5, 137.3, 133.7, 132.1, 130.4, 129.2, 126.0, 123.2, 31.3; HRMS (ESI): Exact mass calcd for $C_{14}H_{11}ClN_3O$ $[M+H]^+$ 272.0591, found 272.0587.

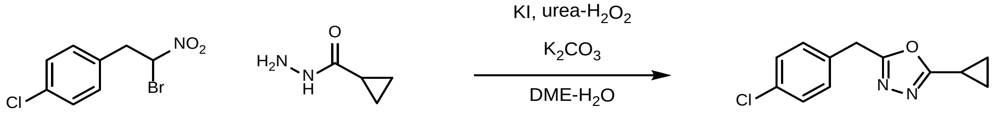

2-(4-Chlorobenzyl)-5-cyclopropyl-1,3,4-oxadiazole (4k). Following the general procedure, the α-bromo nitroalkane **3a** (130 mg, 0.50 mmol) and the monoacyl hydrazide **2k** (60 mg, 0.60 mmol) provided the 2,5-disubstituted 1,3,4-oxadiazole **4k** after flash column chromatography (silica gel, 10-50% ethyl acetate in hexanes) as a colorless solid (95 mg, 81% yield). Mp 80-82 °C (recrystallized from ethyl acetate/hexanes) R_f = 0.37 (50% ethyl acetate/hexanes); IR (film) 3016, 1567, 1491, 1416, 1172, 1092, 1018, 913 cm^{-1}; ^1H NMR (400 MHz, $CDCl_3$) δ 7.33-7.29 (m, 2H), 7.24-7.20 (m, 2H), 4.10 (s, 2H), 2.08 (tt, *J* = 8.4, 5.3 Hz, 1H), 1.13-1.05 (m, 4H); ^{13}C NMR (100 MHz, $CDCl_3$) ppm 169.2, 164.1, 133.6, 132.5, 130.3, 129.2, 31.3, 8.5, 6.4; HRMS (ESI): Exact mass calcd for $C_{12}H_{12}ClN_2O$ $[M+H]^+$ 235.0638, found 235.0630.

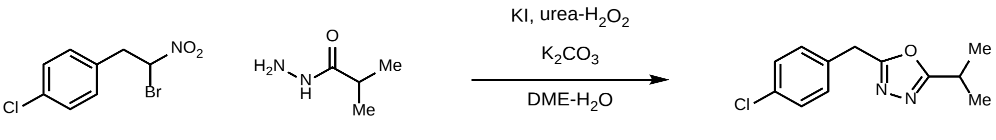

2-(4-Chlorobenzyl)-5-isopropyl-1,3,4-oxadiazole (4l). Following the general procedure, the α-bromo nitroalkane **3a** (53 mg, 0.20 mmol) and the monoacyl hydrazide **2l** (25 mg, 0.24 mmol) provided the 2,5-disubstituted 1,3,4-oxadiazole **4l** after flash column chromatography (silica gel, 10-50% ethyl acetate in hexanes)

as a colorless oil (35 mg, 74% yield). R$_f$ = 0.47 (50% ethyl acetate/hexanes); IR (film) 2977, 1582, 1492, 1418, 1202, 1149, 1093, 1018, 972 cm^{-1}; ^{1}H NMR (400 MHz, $CDCl_3$) δ 7.33-7.29 (m, 2H), 7.25-7.22 (m, 2H), 4.13 (s, 2H), 3.12 (hept, *J* = 7.0 Hz, 1H), 1.35 (d, *J* = 7.0 Hz, 6H); ^{13}C NMR (100 MHz, $CDCl_3$) ppm 171.6, 164.8, 133.6, 132.6, 130.2, 129.1, 31.3, 26.4, 20.0; HRMS (ESI): Exact mass calcd for $C_{12}H_{14}ClN_2O$ $[M+H]^+$ 237.0795, found 237.0789.

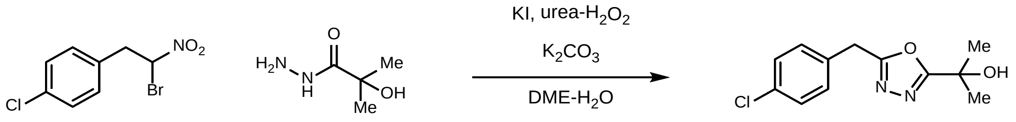

2-(5-(4-Chlorobenzyl)-1,3,4-oxadiazol-2-yl)propan-2-ol (4m). Following the general procedure, the α-bromo nitroalkane **3a** (130 mg, 0.50 mmol) and the monoacyl hydrazide **2m** (71 mg, 0.60 mmol) provided the 2,5-disubstituted 1,3,4-oxadiazole **4m** after flash column chromatography (silica gel, 20-100% ethyl acetate in hexanes) as a colorless solid (76 mg, 60% yield). Mp 74-76 °C (recrystallized from ethyl acetate/hexanes); R$_f$ = 0.30 (50% ethyl acetate/hexanes); IR (film) 3348, 2985, 1579, 1492, 1372, 1137, 1018, 969, 852 cm^{-1}; ^{1}H NMR (400 MHz, $CDCl_3$) δ 7.33-7.30 (m, 2H), 7.25-7.22 (m, 2H), 4.16 (s, 2H), 2.61 (s, 1H), 1.66 (s, 6H); ^{13}C NMR (100 MHz, $CDCl_3$) ppm 171.1, 165.0, 133.4, 131.8, 129.9, 128.9, 68.0, 30.9, 28.0; HRMS (ESI): Exact mass calcd for $C_{12}H_{14}ClN_2O_2$ $[M+H]^+$ 253.0744, found 253.0734.

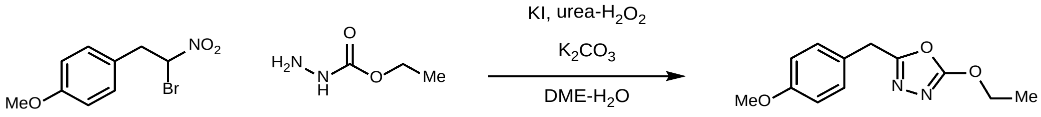

2-Ethoxy-5-(4-methoxybenzyl)-1,3,4-oxadiazole (9). Following the general procedure, the α-bromo nitroalkane **3b** (130 mg, 0.50 mmol) and the monoacyl hydrazide **2a** (62 mg, 0.60 mmol) provided the 2,5-disubstituted 1,3,4-oxadiazole **9** after flash column chromatography (silica gel, 25-50% ethyl acetate in hexanes) as a colorless oil (82 mg, 70% yield). R$_f$ = 0.30 (33% ethyl acetate/hexanes); IR (film) 2988, 1623, 1573, 1246, 1177, 1031 972, 890, 832 cm^{-1}; ^{1}H NMR (400 MHz, $CDCl_3$) δ 7.23-7.20 (m, 2H), 6.88-6.84 (m, 2H), 4.49 (q, *J* = 7.1 Hz, 2H), 3.99 (s, 2H), 3.79 (s, 3H), 1.44 (t, *J* = 7.1 Hz, 3H); ^{13}C NMR (100 MHz, $CDCl_3$) ppm 166.1, 161.5, 159.1, 130.0, 125.9, 114.4, 69.0, 55.4, 31.5, 14.4; HRMS (ESI): Exact mass calcd for $C_{12}H_{15}N_2O_3$ $[M+H]^+$ 235.1083, found 235.1072.

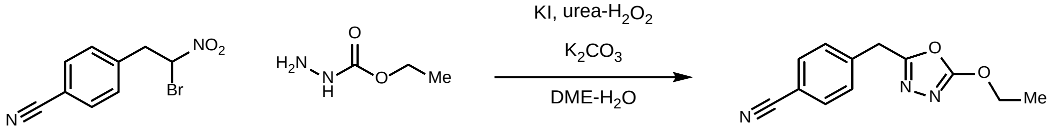

4-((5-Ethoxy-1,3,4-oxadiazol-2-yl)methyl)benzonitrile (10). Following the general procedure, the α-bromo nitroalkane **3c** (130 mg, 0.50 mmol) and the monoacyl hydrazide **2a** (62 mg, 0.60 mmol) provided the 2,5-disubstituted 1,3,4-oxadiazole **10** after flash column chromatography (silica gel, 25-50% ethyl acetate in hexanes) as a colorless solid (71 mg, 62% yield). Mp 99-101 °C (recrystallized from ethyl acetate/hexanes); R$_f$ = 0.17 (33% ethyl acetate/hexanes); IR (film) 2991, 2228, 1631, 1569, 1324, 1188 cm^{-1}; ^{1}H NMR (400 MHz, $CDCl_3$) δ 7.66-7.63 (m, 2H), 7.44-7.41 (m, 2H), 4.52 (q, *J* = 7.1 Hz, 2H), 4.12 (s, 2H), 1.46 (t, *J* = 7.1 Hz, 3H); ^{13}C NMR (100 MHz, $CDCl_3$) ppm 166.3, 159.8, 139.2, 132.8, 129.8, 118.6, 111.9, 69.4, 32.4, 14.4; HRMS (CI): Exact mass calcd for $C_{12}H_{12}N_3O_2$ $[M+H]^+$ 230.0924, found 230.0913.

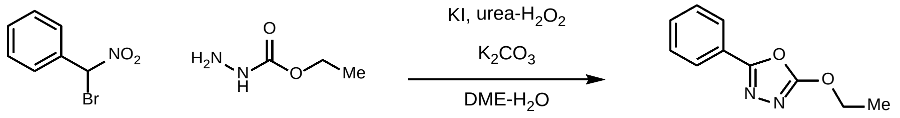

2-Ethoxy-5-phenyl-1,3,4-oxadiazole (11). Following the general procedure, the α-bromo nitroalkane **3d**[5] (43 mg, 0.20 mmol) and the monoacyl hydrazide **2a** (25 mg, 0.24 mmol) provided the 2,5-disubstituted 1,3,4-oxadiazole **11** after flash column chromatography (silica gel, 25% ethyl acetate in hexanes) as a colorless solid (26 mg, 69% yield). mp 36-39 °C (crystallized on standing); R_f = 0.67 (33% ethyl acetate/hexanes); IR (film) 2987, 1611, 1484, 1280, 1018, 891, 689 cm^{-1}; ^{1}H NMR (400 MHz, $CDCl_3$) δ 7.95-7.93 (m, 2H), 7.50-7.44 (m, 3H), 4.62 (q, *J* = 7.1 Hz, 2H), 1.53 (t, *J* = 7.1 Hz, 3H); ^{13}C NMR (100 MHz, $CDCl_3$) ppm 165.8, 160.6, 131.3, 129.0, 126.2, 124.3, 69.4, 14.5; HRMS (CI): Exact mass calcd for $C_{10}H_{11}N_2O_2$ $[M+H]^+$ 191.0815, found 191.0817.

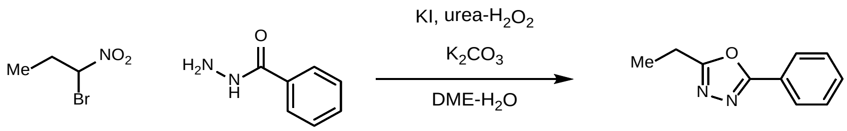

2-Ethyl-5-phenyl-1,3,4-oxadiazole (12). Following the general procedure, the α-bromo nitroalkane **3e**[6] (87 mg, 0.50 mmol) and the monoacyl hydrazide **2e** (82 mg, 0.60 mmol) provided the 2,5-disubstituted 1,3,4-oxadiazole **12** after flash column chromatography (silica gel, 15% acetone in hexanes) as a colorless oil (45 mg, 52% yield). ^{1}H NMR (400 MHz, $CDCl_3$) δ 8.05-8.02 (m, 2H), 7.55-7.47 (m, 3H), 2.96 (q, *J* = 7.6 Hz, 2H), 1.44 (t, *J* = 7.6 Hz, 3H); ^{13}C NMR (100 MHz, $CDCl_3$) ppm 167.9, 164.8, 131.6, 129.1, 126.8, 124.2, 19.3, 11.0. Spectral data matched literature[7,8].

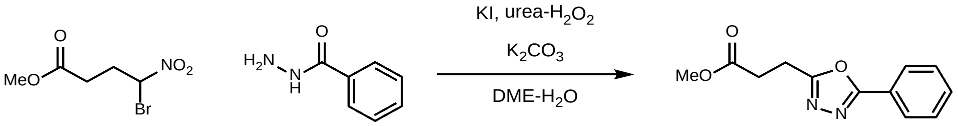

Methyl 3-(5-phenyl-1,3,4-oxadiazol-2-yl)propanoate (13). Following the general procedure, the α-bromo nitroalkane **3f**[9] (90 mg, 0.40 mmol) and the monoacyl hydrazide **2e** (65 mg, 0.48 mmol) provided the 2,5-disubstituted 1,3,4-oxadiazole after flash column chromatography (silica gel, 20-100% ethyl acetate in hexanes) as a colorless solid (55 mg, 60% yield). Mp 64-66 °C (recrystallized from ethyl acetate/hexanes) R_f = 0.43 (50% ethyl acetate/hexanes); IR (film) 2951, 1737, 1566, 1441, 1366, 1173, 1017, 913 cm^{-1}; ^{1}H NMR (400 MHz, $CDCl_3$) δ 8.04-8.01 (m, 2H), 7.55-7.47 (m, 3H), 3.74 (s, 3H), 3.26 (t, *J* = 7.4 Hz, 2H), 2.94 (t, *J* = 7.4 Hz, 2H); ^{13}C NMR (100 MHz, $CDCl_3$) ppm 172.0, 165.5, 165.0, 131.7, 129.1, 126.9, 124.0, 52.2, 30.4, 21.1; HRMS (ESI): Exact mass calcd for $C_{12}H_{12}N_2NaO_3$ $[M+Na]^+$ 255.0746, found 255.0746.

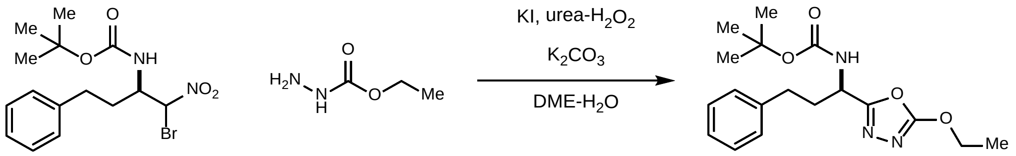

[5] Kunetsky, R. A.; Dilman, A. D.; Ioffe, S. L.; Struchkova, M. I.; Strelenko, Y. A.; Tartakovsky, V. A. *Org. Lett.* **2003**, *5*, 4907.
[6] Erickson, A. S.; Kornblum, N. *J. Org. Chem.* **1977**, *42*, 3764.
[7] Park, Y.-D.; Kim, J.-J.; Chung, H.-A.; Kweon, D.-H.; Cho, S.-D.; Lee, S.-G.; Yoon, Y.-J. *Synthesis* **2003**, 0560.
[8] Spectroscopic data is nearly identical to that previously reported. Quartet at 2.96 ppm and triplet at 1.44 ppm measured *J* = 7.6 (us) vs. 6.0 Hz (ref 7).
[9] Shen, B.; Makley, D. M.; Johnston, J. N. *Nature* **2010**, *465*, 1027.

***tert*-Butyl (*R*)-(1-(5-ethoxy-1,3,4-oxadiazol-2-yl)-3-phenylpropyl)carbamate (14).** Following the general procedure, the α-bromo nitroalkane **3f**[10] (1:1 dr, 99/99 % ee for each diastereomers, 370 mg, 1.0 mmol) and the monoacyl hydrazide **2a** (130 mg, 1.2 mmol) provided the 2,5-disubstituted 1,3,4-oxadiazole **14** after flash column chromatography (silica gel, 20-50% ethyl acetate in hexanes) as a colorless solid (187 mg, 54% yield) that was determined to be >99% ee by chiral HPLC analysis (Chiralpak IC: 10% 2-propanol/hexanes, 1.0 mL/min, t_r (major) = 20.3 min, t_r (minor) = 24.9 min). $[\alpha]_D^{20}$ +23.1 (*c* 0.95, $CHCl_3$); mp 69-71 °C (recrystallized from ethyl acetate/hexanes); R_f = 0.50 (50% ethyl acetate/hexanes); IR (film) 3316, 2977, 1707, 1621, 1566, 1517, 1367, 1249, 1168, 1027 cm^{-1}; ^{1}H NMR (400 MHz, $CDCl_3$) δ 7.30-7.26 (m, 2H), 7.21-7.16 (m, 3H), 5.05-4.87 (m, 2H), 4.52 (q, *J* = 7.1 Hz, 2H), 2.78-2.66 (m, 2H), 2.30-2.21 (m, 1H), 2.14-2.04 (m, 1H), 1.47 (t, *J* = 7.1 Hz, 3H), 1.45 (s, 9H); ^{13}C NMR (100 MHz, $CDCl_3$) ppm 165.9, 162.1, 155.0, 140.5, 128.6, 128.5, 126.3, 80.4, 69.3, 47.1, 35.2, 31.6, 28.4, 14.3; HRMS (ESI): Exact mass calcd for $C_{18}H_{25}N_3NaO_4$ $[M+Na]^+$ 370.1743, found 370.1733.

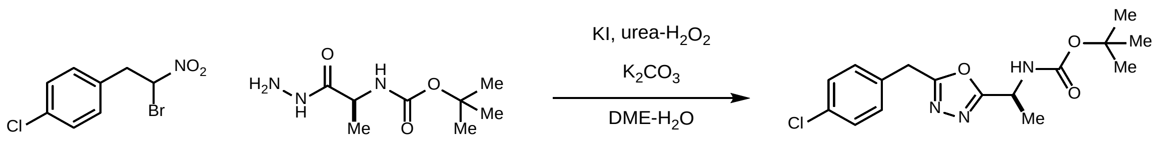

***tert*-Butyl (*S*)-(1-(5-(4-chlorobenzyl)-1,3,4-oxadiazol-2-yl)ethyl)carbamate (15).** Following the general procedure, the α-bromo nitroalkane **3a** (53 mg, 0.20 mmol) and the monoacyl hydrazide **2o** (purchased from Enamine Ltd.) (49 mg, 0.24 mmol) provided the 2,5-disubstituted 1,3,4-oxadiazole **15** after flash column chromatography (silica gel, 20-100% ethyl acetate in hexanes) as a colorless solid (29 mg, 43% yield) that was determined to be >99% ee by chiral HPLC analysis (Chiralpak AD-H: 10% 2-propanol/hexanes, 1.0 mL/min, t_r (minor) = 15.6 min, t_r (major) = 17.5 min). $[\alpha]_D^{20}$ -26.3 (*c* 0.95, chloroform); mp 107-109 °C (recrystallized from ethyl acetate/hexanes) R_f = 0.37 (50% ethyl acetate/hexanes); IR (film) 3311, 2979, 1704, 1503, 1369, 1250, 1166, 1088, 1018, 913 cm^{-1}; ^{1}H NMR (400 MHz, $CDCl_3$) δ 7.33-7.29 (m, 2H), 7.25-7.22 (m, 2H), 5.09-4.89 (m, 2H), 4.15 (s, 2H), 1.55 (d, *J* = 6.8 Hz, 3H), 1.43 (s, 9H); ^{13}C NMR (100 MHz, $CDCl_3$) ppm 168.1, 165.3, 154.8, 133.7, 132.2, 130.3, 129.2, 80.5, 43.2, 31.3, 28.4, 19.7; HRMS (ESI): Exact mass calcd for $C_{16}H_{20}ClN_3NaO_3$ $[M+Na]^+$ 360.1091, found 360.1093.

Experiment in Scheme 2

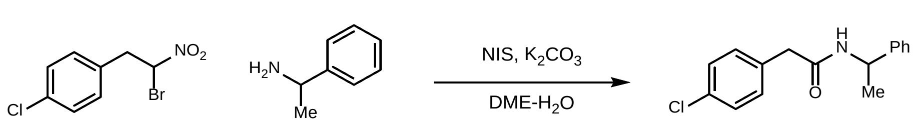

Synthesis of 2-(4-chlorophenyl)-*N*-(1-phenylethyl)acetamide (7) (eq 4). To a mixture of α-bromo nitroalkane **3a** (26 mg, 0.10 mmol), α-methylbenzylamine (15 mg, 0.12 mmol, 1.2 equiv), potassium carbonate (28 mg, 0.20 mmol, 2.0 equiv) and water (9.0 mg, 0.50 mmol, 5.0 equiv) in 1,2-dimethoxyethane (1.0 mL) was added *N*-iodosuccinimide (23 mg, 0.10 mmol, 1.0 equiv) at 0 °C. After stirring for 24 h at 0 °C, aqueous sodium thiosulfate was added and the mixture was extracted with ethyl acetate. The combined organic layers were washed with brine, dried over sodium sulfate and concentrated *in vacuo*. The residue was purified by flash column chromatography to give 2-(4-chlorophenyl)-*N*-(1-phenylethyl)acetamide **8** as a colorless solid (13 mg, 47% yield). Characterization data matched with the literature[11].

[10] Schwieter, K. E.; Johnston, J. N. *ACS Catal.* **2015**, *5*, 6559.
[11] Katkar, K. V.; Chaudhari, P. S.; Akamanchi, K. G. *Green Chem.* **2011**, *13*, 835.

Experiments in Scheme 3

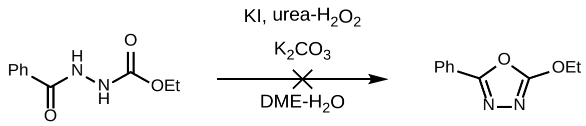

Exposure of diacyl hydrazide 16 to the reaction conditions (eq 6). To a mixture of diacyl hydrazide **16**[12] (21 mg, 0.10 mmol, 1.0 equiv), potassium iodide (33 mg, 0.20 mmol, 2.0 equiv), potassium carbonate (28 mg, 0.20 mmol, 2.0 equiv) and water (9.0 mg, 0.50 mmol, 5.0 equiv) in 1,2-dimethoxyethane (1 mL) was added urea-hydrogen peroxide (9.4 mg, 0.10 mmol, 1.0 equiv) at 0 °C and stirred for 24 h. Aqueous sodium thiosulfate was added and the mixture was extracted with ethyl acetate. The combined organic layers were washed with brine, dried over sodium sulfate and concentrated *in vacuo*. The oxadiazole **11** was not observed (^{1}H NMR) in the crude reaction mixture.

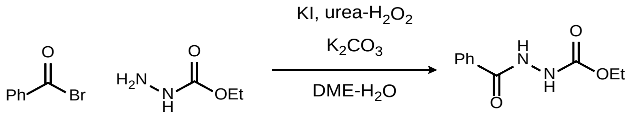

Reaction of acyl bromide 17 and acyl hydrazide 2a under the reaction conditions (eq 7). To a mixture of benzoyl bromide **17** (19 mg, 0.10 mmol), potassium iodide (33 mg, 0.20 mmol, 2.0 equiv), potassium carbonate (28 mg, 0.20 mmol, 2.0 equiv), water (9.0 mg, 0.50 mmol, 5.0 equiv) and urea-hydrogen peroxide (9.4 mg, 0.10 mmol, 1.0 equiv) in 1,2-dimethoxyethane (1 mL) was added monoacyl hydrazide **2a** (13 mg, 0.12 mmol, 1.2 equiv) at 0 °C. After stirring for 20 h at 0 °C, aqueous sodium thiosulfate was added and the mixture was extracted with ethyl acetate. The combined organic layers were washed with brine, dried over sodium sulfate and concentrated *in vacuo*. By ^{1}H NMR analysis of the crude product using dibromomethane as quantitative standard, the yield of the diacyl hydrazide **16** was calculated to be 40-50% and the oxadiazole **11** was not observed.

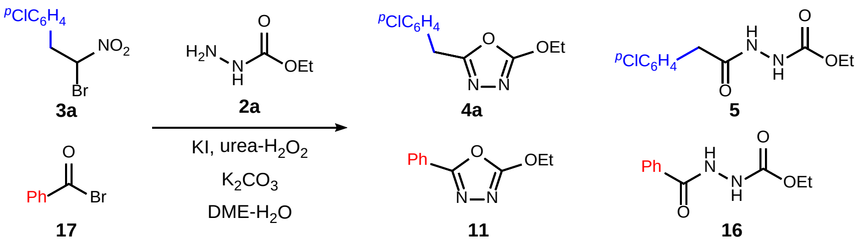

Crossover experiment using α-bromo nitroalkane 3a and acyl bromide 17 (eq 8). To a mixture of α-bromo nitroalkane **3a** (26 mg, 0.10 mmol, 1.0 equiv), benzoyl bromide **17** (19 mg, 0.10 mmol), potassium iodide (33 mg, 0.20 mmol, 2.0 equiv), potassium carbonate (55 mg, 0.40 mmol, 4.0 equiv), water (9.0 mg, 0.50 mmol, 5.0 equiv) and urea-hydrogen peroxide (9.4 mg, 0.10 mmol, 1.0 equiv) in 1,2-dimethoxyethane (2 mL) was added monoacyl hydrazide **2a** (25 mg, 0.24 mmol, 2.4 equiv) at 0 °C. After stirring for 20 h at 0 °C, aqueous sodium thiosulfate was added and the mixture was extracted with ethyl acetate. The combined organic layers were washed with brine, dried over sodium sulfate and concentrated *in vacuo*. By ^{1}H NMR analysis of the crude product using dibromomethane as quantitative standard, the yield of the oxadiazole **4a** and the diacyl hydrazide **16** were calculated to be 44% (from **3a**) and 49% (from **17**) respectively. The diacyl hydrazide **5** and the oxadiazole **11** were not observed.

[12] Hua, G.; Li, Y.; Fuller, A. L.; Slawin, A. M. Z.; Woollins, J. D. *Eur. J. Org. Chem.* **2009**, 1612.

A Convergent Synthesis of 1,3,4-Oxadiazoles from Acyl Hydrazides under Semiaqueous Conditions

Kazuyuki Tokumaru* and Jeffrey N. Johnston*

Department of Chemistry and Vanderbilt Institute of Chemical Biology,
Vanderbilt University, Nashville, Tennessee 37235

SI2-X

Figure 1. ^{1}H NMR (400 MHz, $CDCl_3$) of 3a 3
Figure 2. ^{13}C NMR (100 MHz, $CDCl_3$) of 3a 4
Figure 3. ^{1}H NMR (400 MHz, $CDCl_3$) of 3b 5
Figure 4. ^{13}C NMR (100 MHz, $CDCl_3$) of 3b 6
Figure 5. ^{1}H NMR (400 MHz, $CDCl_3$) of 3c 7
Figure 6. ^{13}C NMR (100 MHz, $CDCl_3$) of 3c 8
Figure 7. ^{1}H NMR (400 MHz, $CDCl_3$) of 4a 9
Figure 8. ^{13}C NMR (100 MHz, $CDCl_3$) of 4a 10
Figure 9. ^{1}H NMR (400 MHz, $CDCl_3$) of 4b 11
Figure 10. ^{13}C NMR (100 MHz, $CDCl_3$) of 4b 12
Figure 11. ^{1}H NMR (400 MHz, $CDCl_3$) of 4c 13
Figure 12. ^{13}C NMR (100 MHz, $CDCl_3$) of 4c 14
Figure 13. ^{1}H NMR (400 MHz, $CDCl_3$) of 4d 15
Figure 14. ^{13}C NMR (100 MHz, $CDCl_3$) of 4d 16
Figure 15. ^{1}H NMR (400 MHz, $CDCl_3$) of 4e 17
Figure 16. ^{13}C NMR (100 MHz, $CDCl_3$) of 4e 18
Figure 17. ^{1}H NMR (400 MHz, $CDCl_3$) of 4f 19
Figure 18. ^{13}C NMR (100 MHz, $CDCl_3$) of 4f 20
Figure 19. ^{1}H NMR (400 MHz, $CDCl_3$) of 4g 21
Figure 20. ^{13}C NMR (100 MHz, $CDCl_3$) of 4g 22
Figure 21. ^{1}H NMR (400 MHz, $CDCl_3$) of 4h 23
Figure 22. ^{13}C NMR (100 MHz, $CDCl_3$) of 4h 24
Figure 23. ^{1}H NMR (400 MHz, $CDCl_3$) of 4i 25
Figure 24. ^{13}C NMR (100 MHz, $CDCl_3$) of 4i 26
Figure 25. ^{1}H NMR (400 MHz, $CDCl_3$) of 4j 27
Figure 26. ^{13}C NMR (100 MHz, $CDCl_3$) of 4j 28
Figure 27. ^{1}H NMR (400 MHz, $CDCl_3$) of 4k 29
Figure 28. ^{13}C NMR (100 MHz, $CDCl_3$) of 4k 30
Figure 29. ^{1}H NMR (400 MHz, $CDCl_3$) of 4l 31
Figure 30. ^{13}C NMR (100 MHz, $CDCl_3$) of 4l 32

Figure 31. ^{1}H NMR (400 MHz, $CDCl_3$) of 4m....33
Figure 32. ^{13}C NMR (100 MHz, $CDCl_3$) of 4m....34
Figure 33. ^{1}H NMR (400 MHz, $CDCl_3$) of 9....35
Figure 34. ^{13}C NMR (100 MHz, $CDCl_3$) of 9....36
Figure 35. ^{1}H NMR (400 MHz, $CDCl_3$) of 10....37
Figure 36. ^{13}C NMR (100 MHz, $CDCl_3$) of 10....38
Figure 37. ^{1}H NMR (400 MHz, $CDCl_3$) of 11....39
Figure 38. ^{13}C NMR (100 MHz, $CDCl_3$) of 11....40
Figure 39. ^{1}H NMR (400 MHz, $CDCl_3$) of 12....41
Figure 40. ^{13}C NMR (100 MHz, $CDCl_3$) of 12....42
Figure 41. ^{1}H NMR (400 MHz, $CDCl_3$) of 13....43
Figure 42. ^{13}C NMR (100 MHz, $CDCl_3$) of 13....44
Figure 43. ^{1}H NMR (400 MHz, $CDCl_3$) of 14....45
Figure 44. ^{13}C NMR (100 MHz, $CDCl_3$) of 14....46
Figure 45. ^{1}H NMR (400 MHz, $CDCl_3$) of 15....47
Figure 46. ^{13}C NMR (100 MHz, $CDCl_3$) of 15....48
Figure 47. HPLC trace of 14....49
Figure 48. HPLC trace of 15....50

Figure 1. ^{1}H NMR (400 MHz, $CDCl_3$) of **3a**

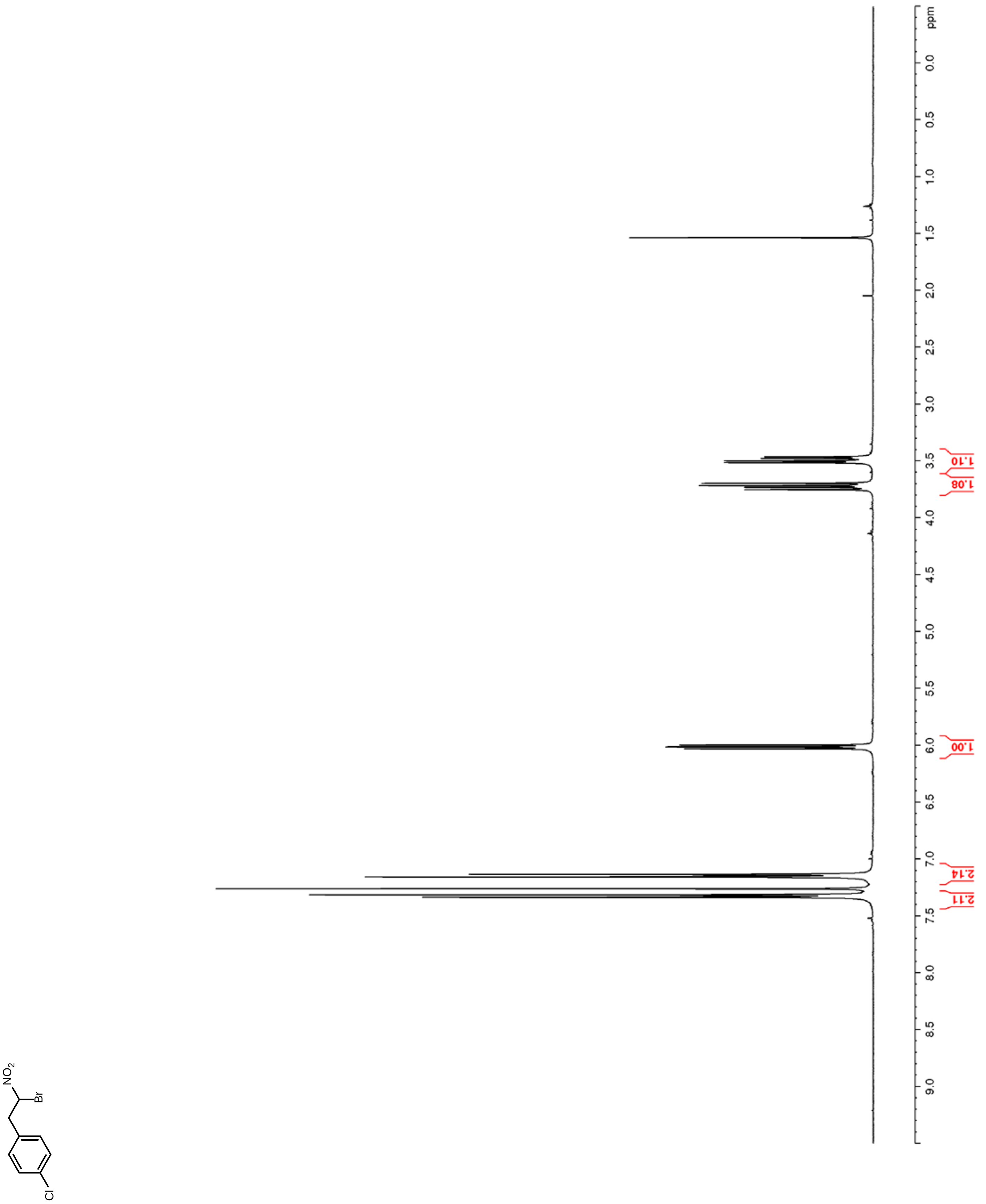

Figure 2. ^{13}C NMR (100 MHz, $CDCl_3$) of **3a**

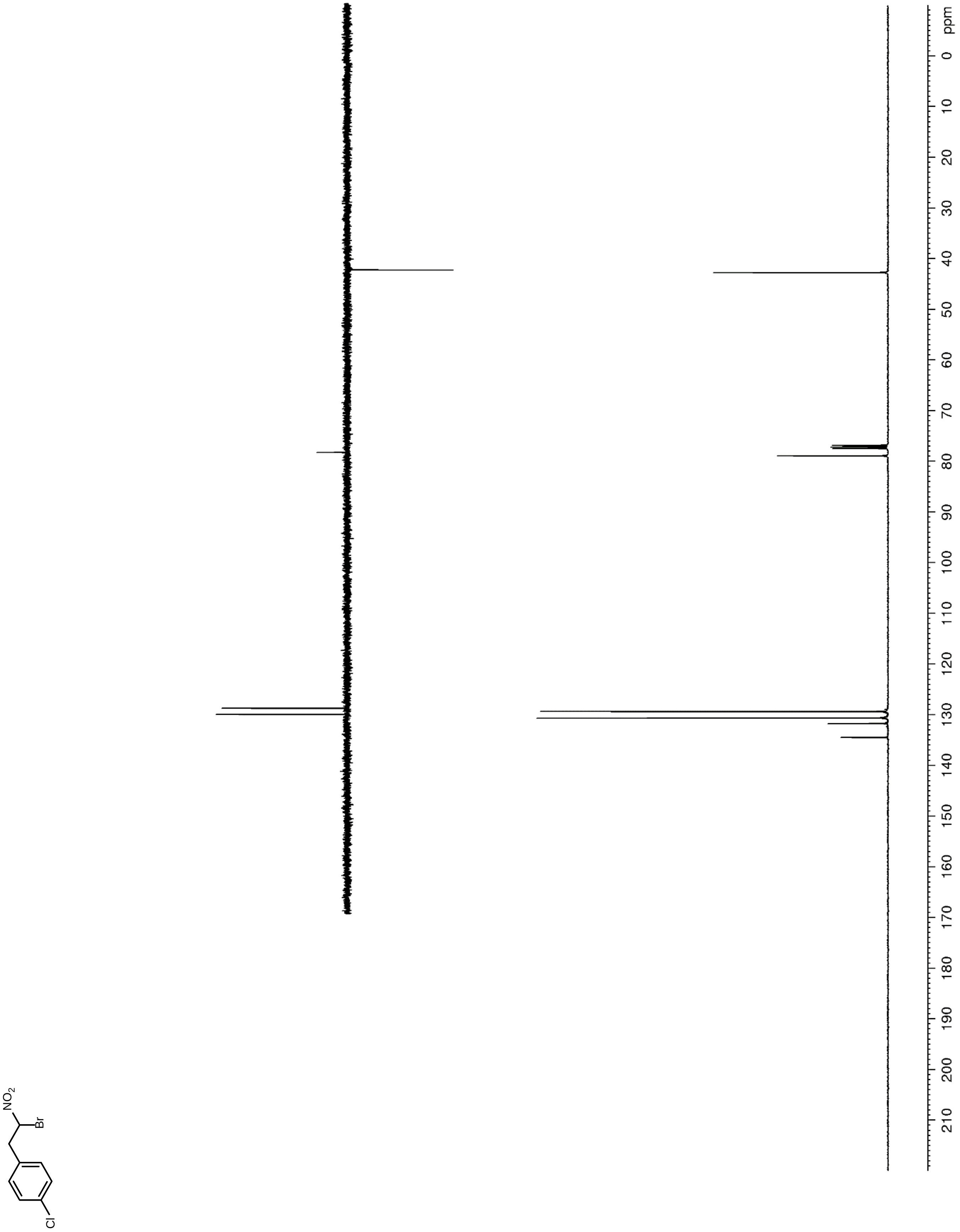

Figure 3. ^{1}H NMR (400 MHz, $CDCl_3$) of **3b**

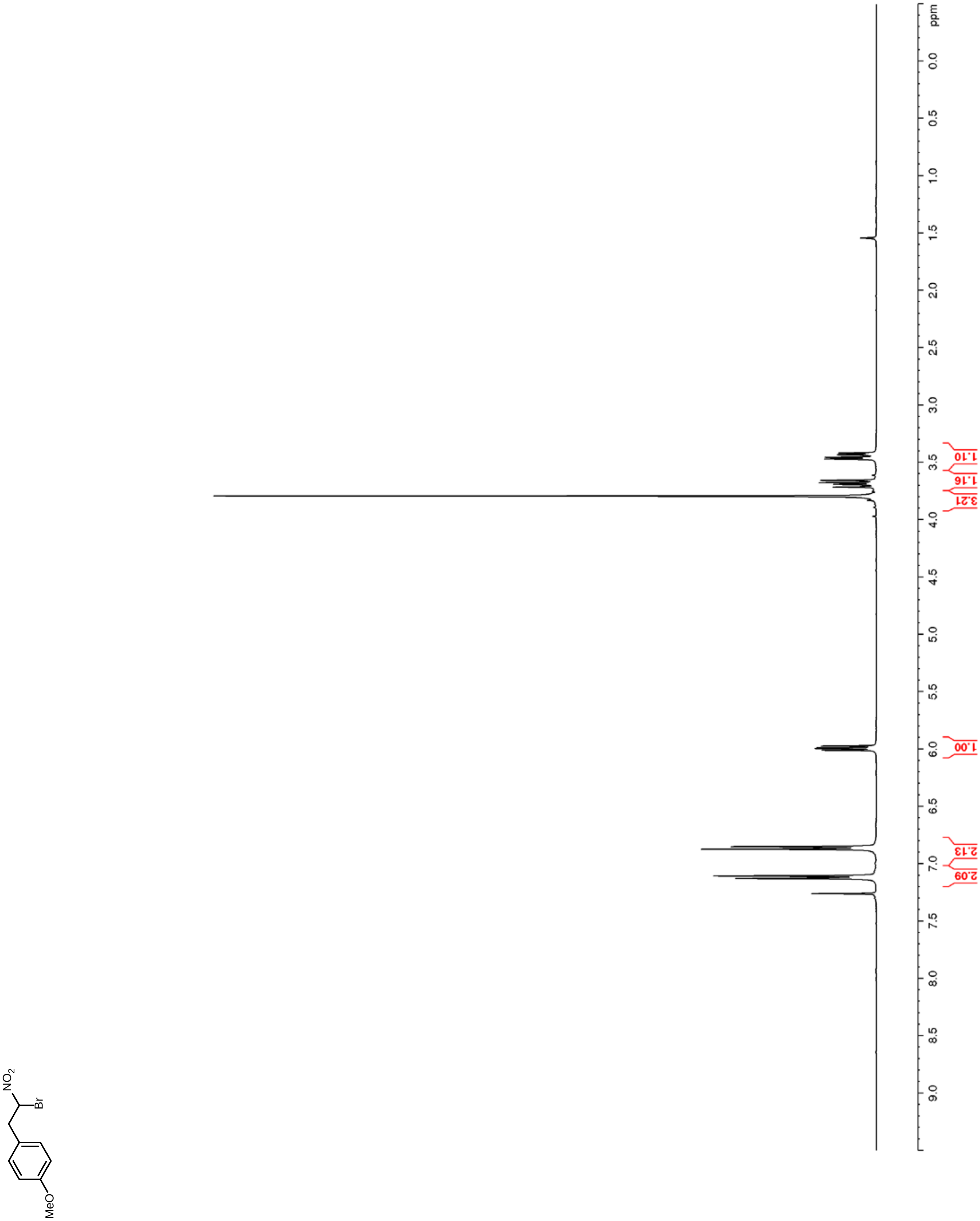

Figure 4. ^{13}C NMR (100 MHz, $CDCl_3$) of **3b**

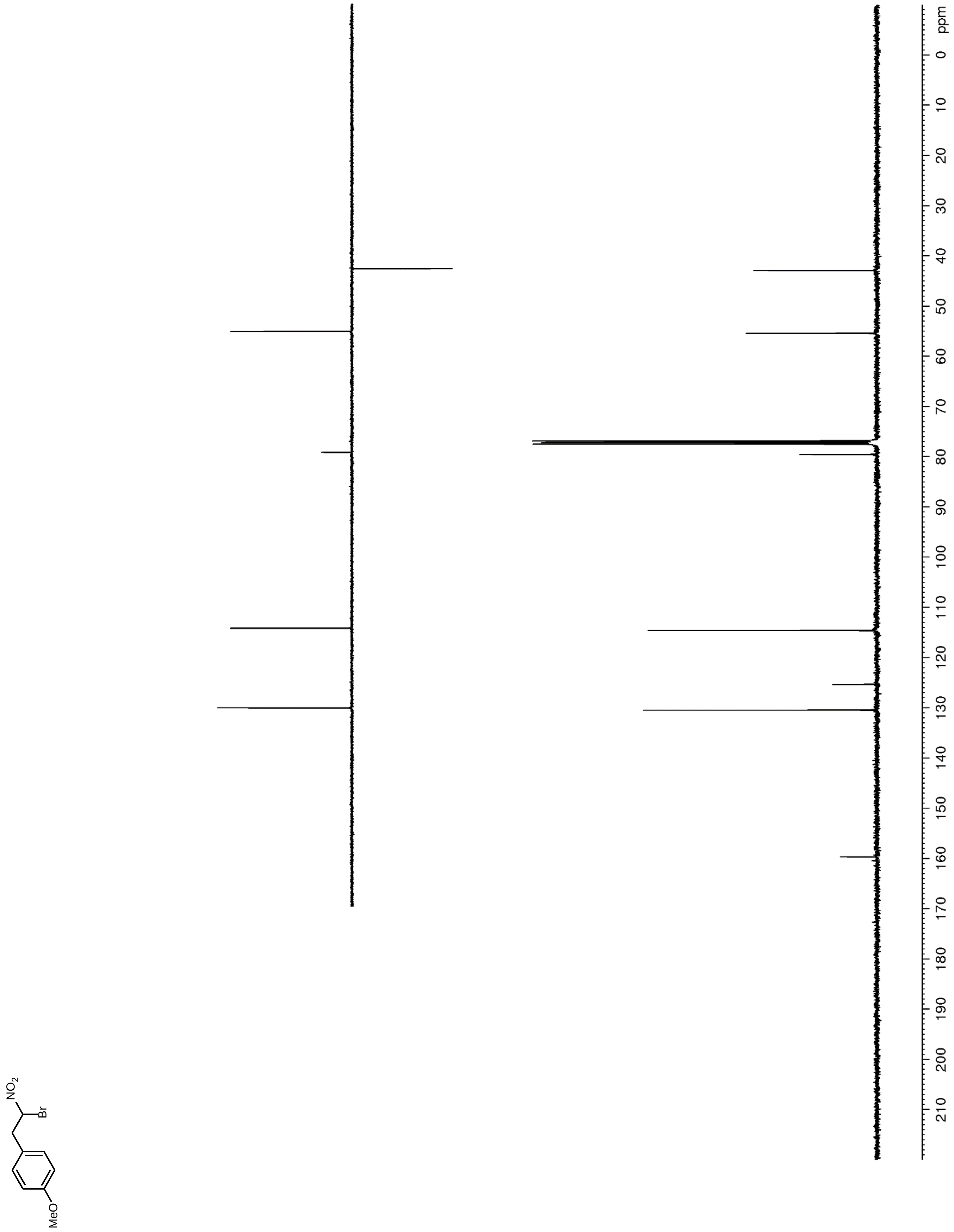

Figure 5. ^{1}H NMR (400 MHz, $CDCl_3$) of **3c**

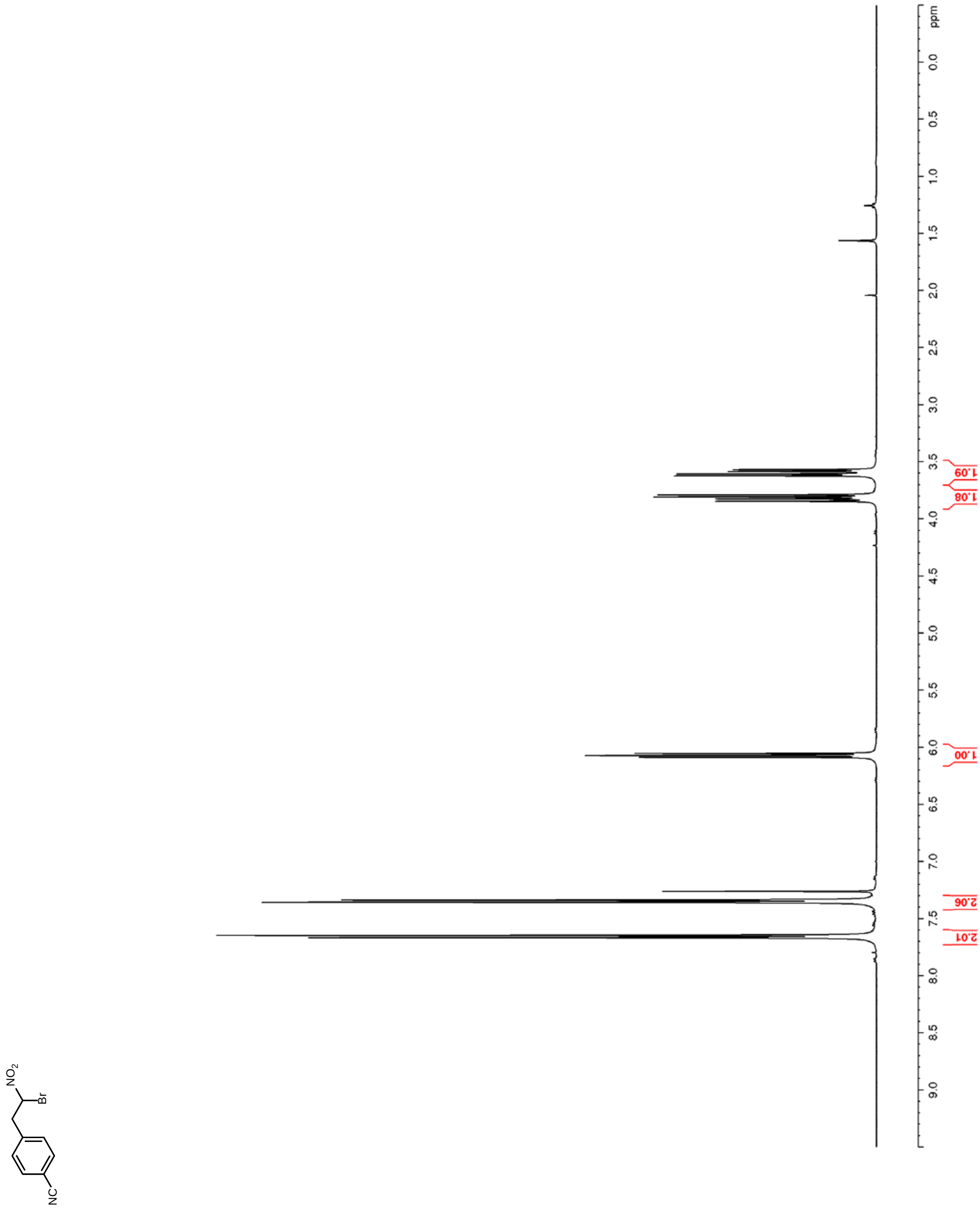

Figure 6. ^{13}C NMR (100 MHz, $CDCl_3$) of **3c**

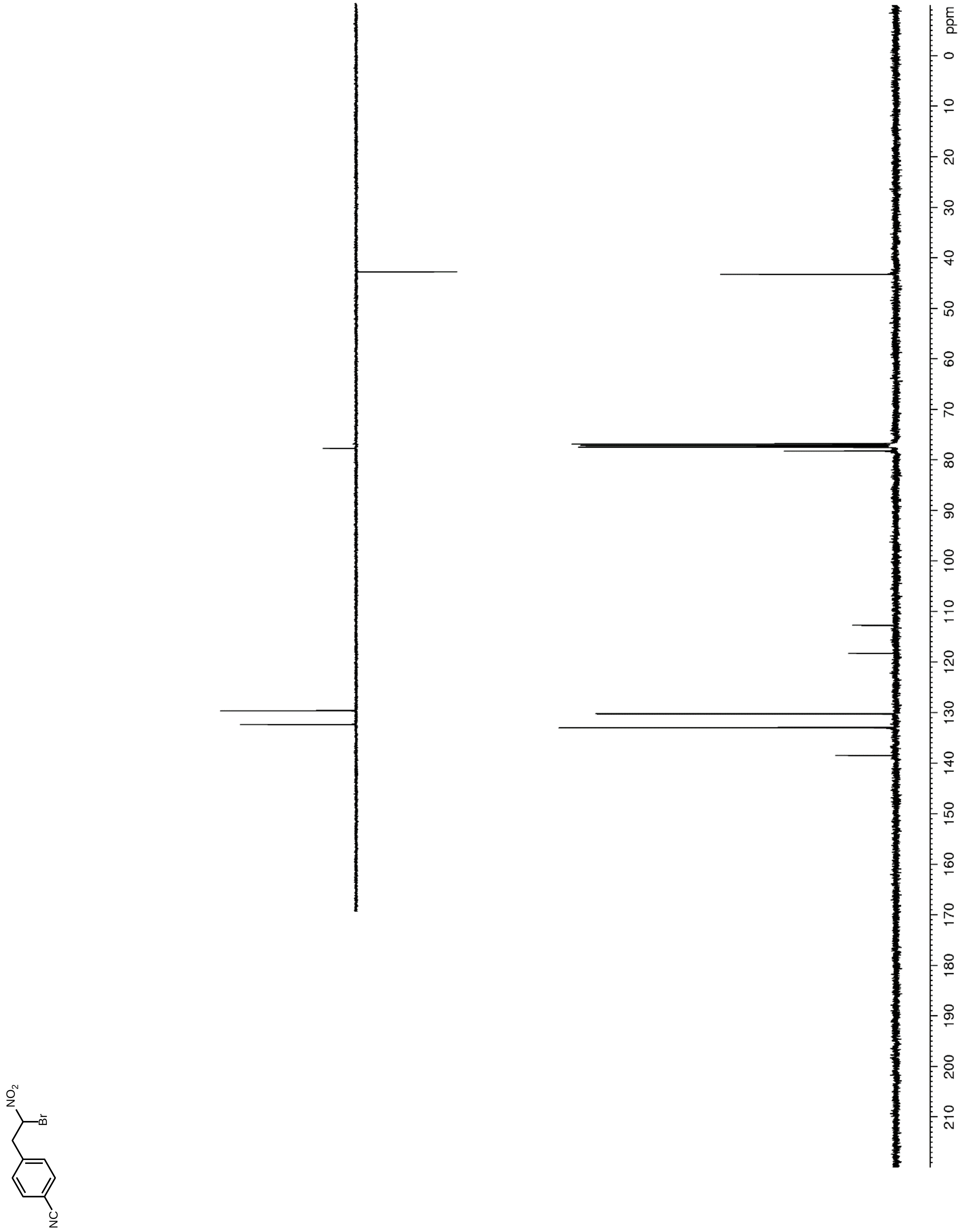

Figure 7. ^{1}H NMR (400 MHz, $CDCl_3$) of **4a**

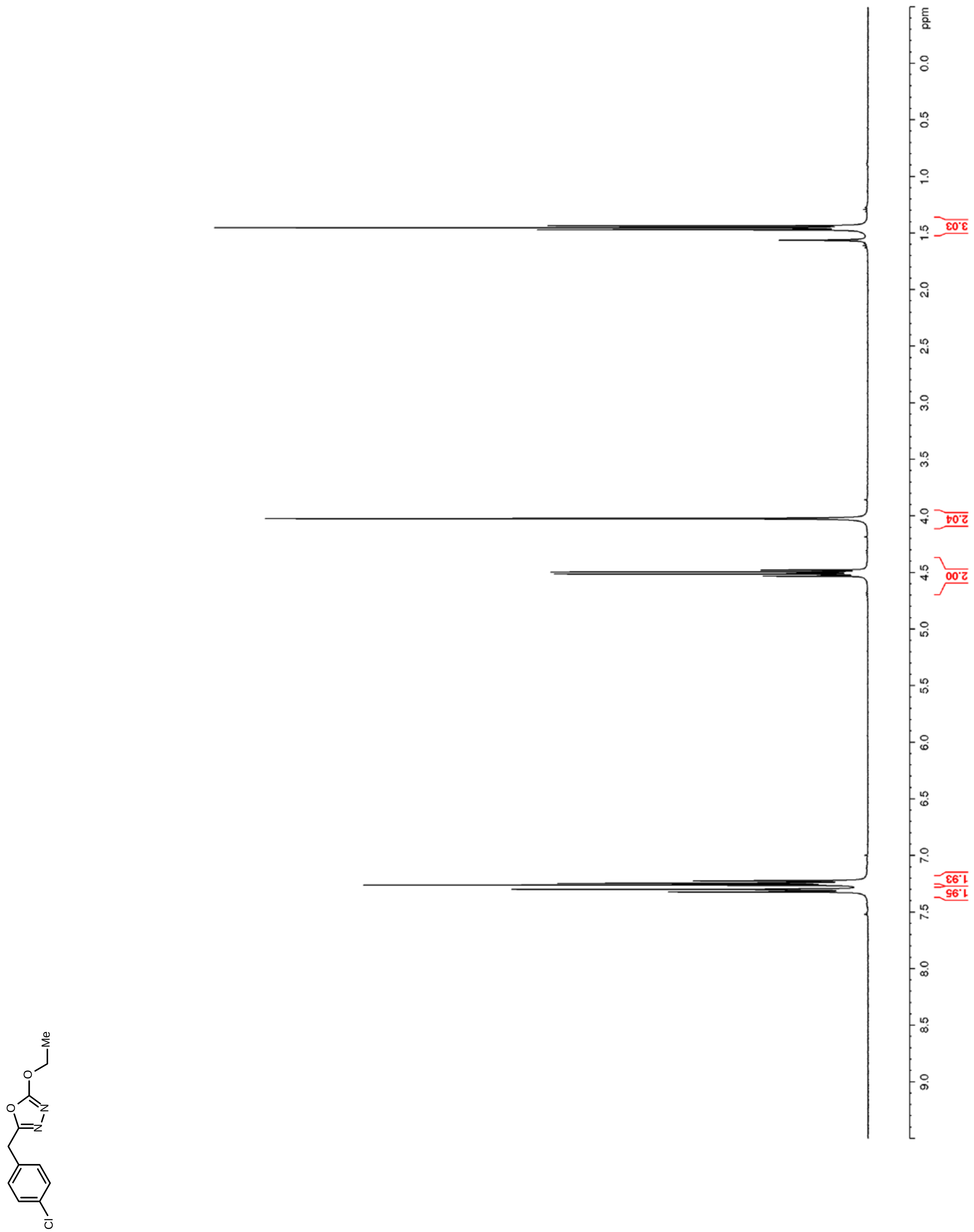

Figure 8. ^{13}C NMR (100 MHz, $CDCl_3$) of **4a**

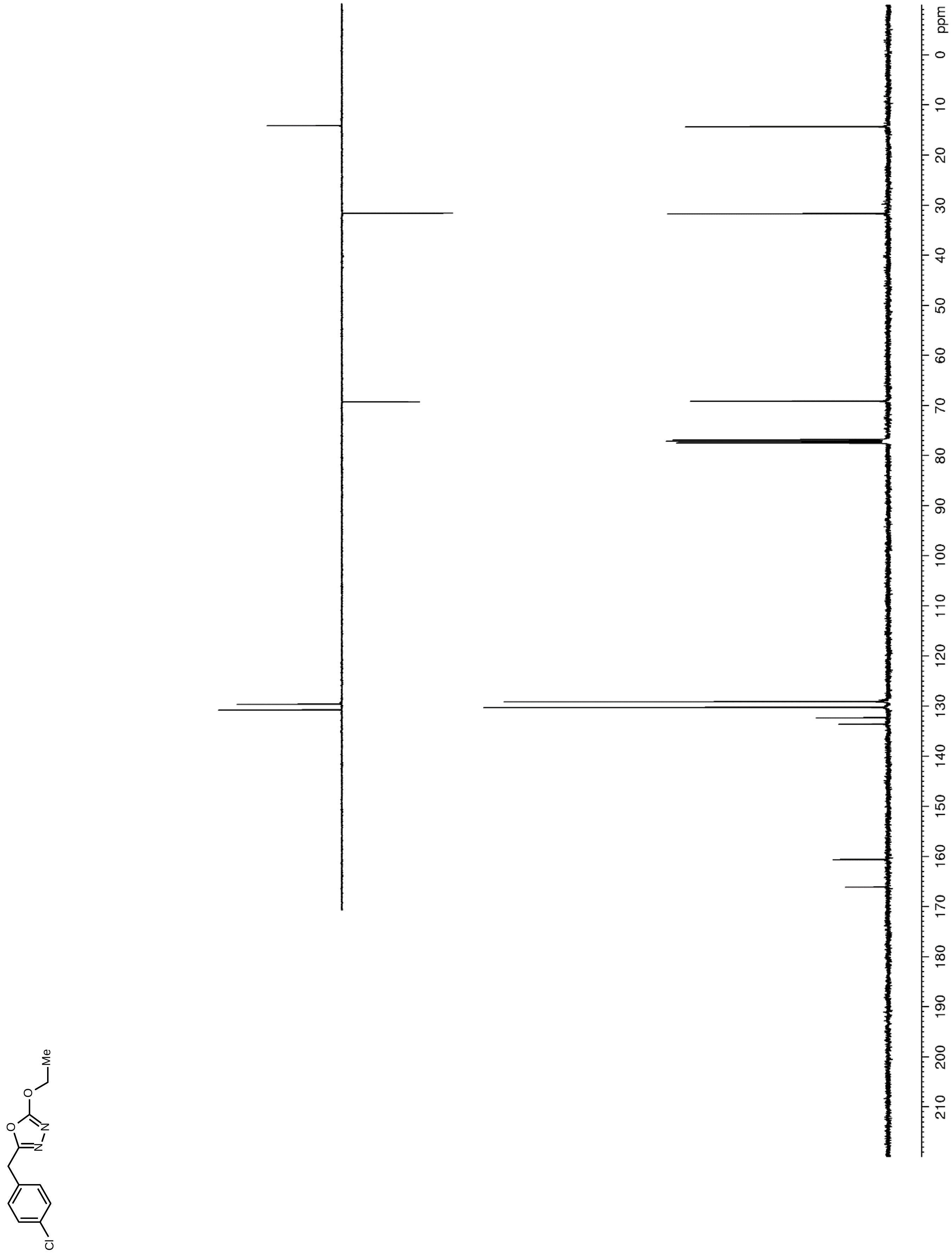

Figure 9. ^{1}H NMR (400 MHz, $CDCl_3$) of **4b**

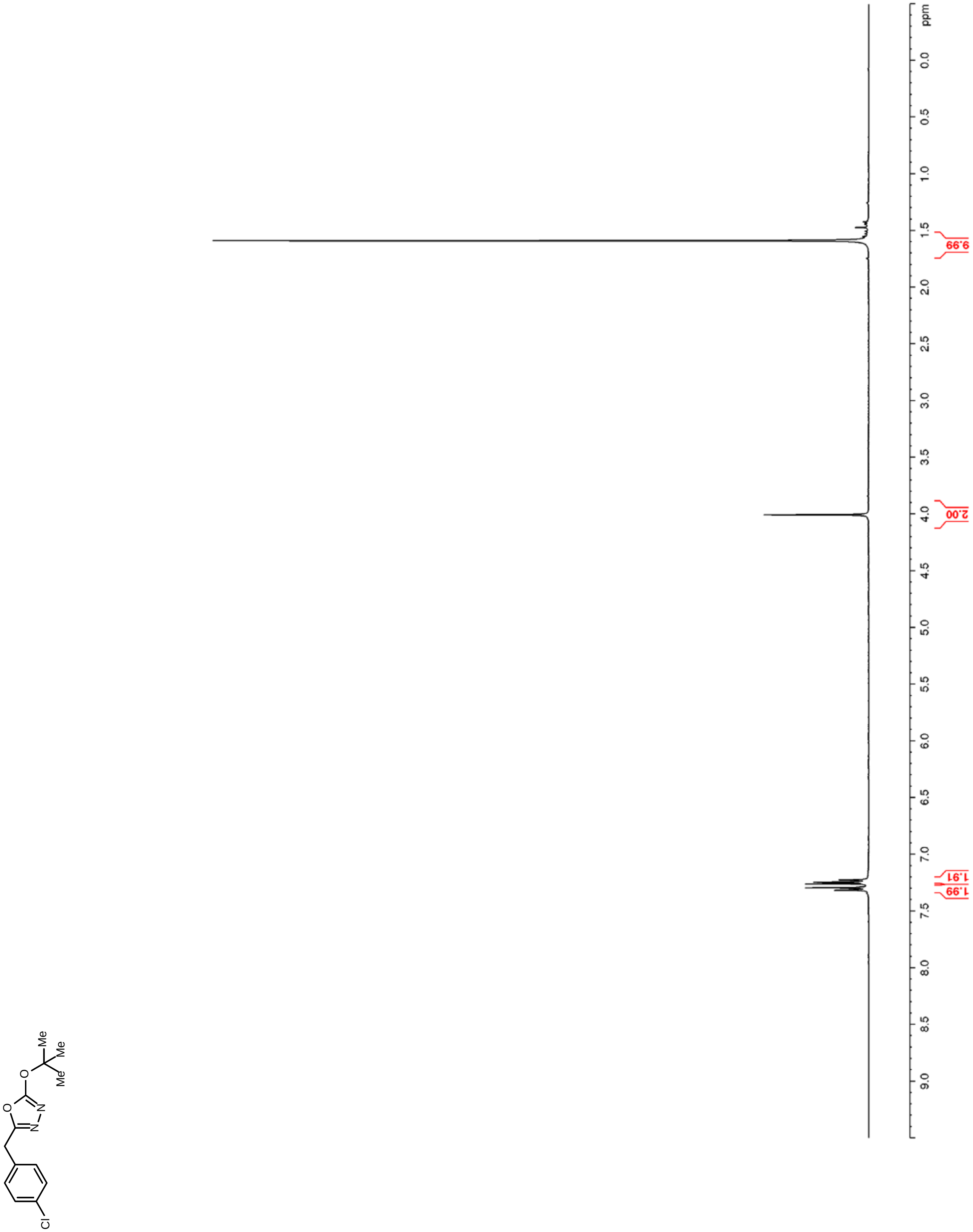

Figure 10. ^{13}C NMR (100 MHz, $CDCl_3$) of **4b**

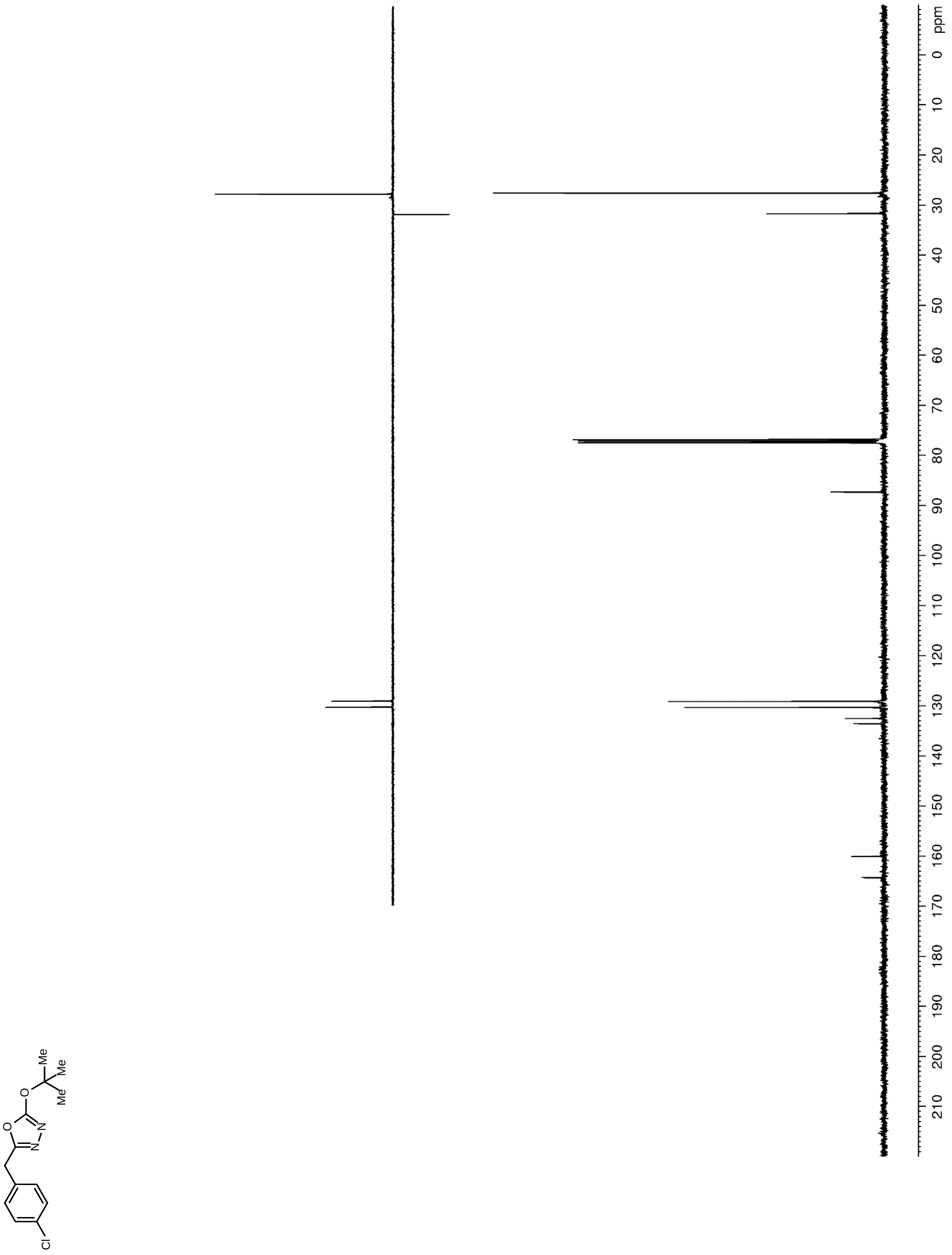

Figure 11. ^{1}H NMR (400 MHz, $CDCl_3$) of **4c**

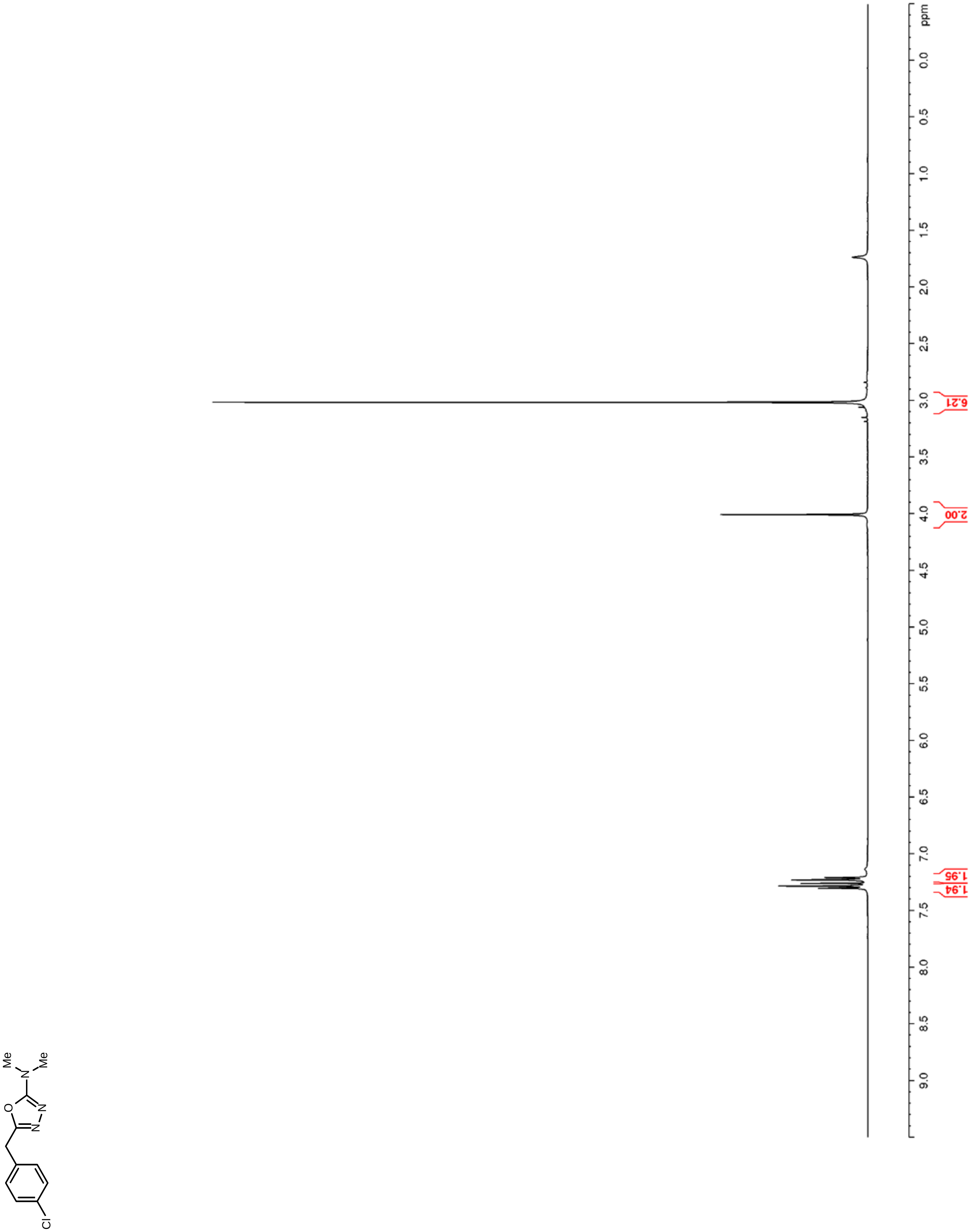

Figure 12. ^{13}C NMR (100 MHz, $CDCl_3$) of **4c**

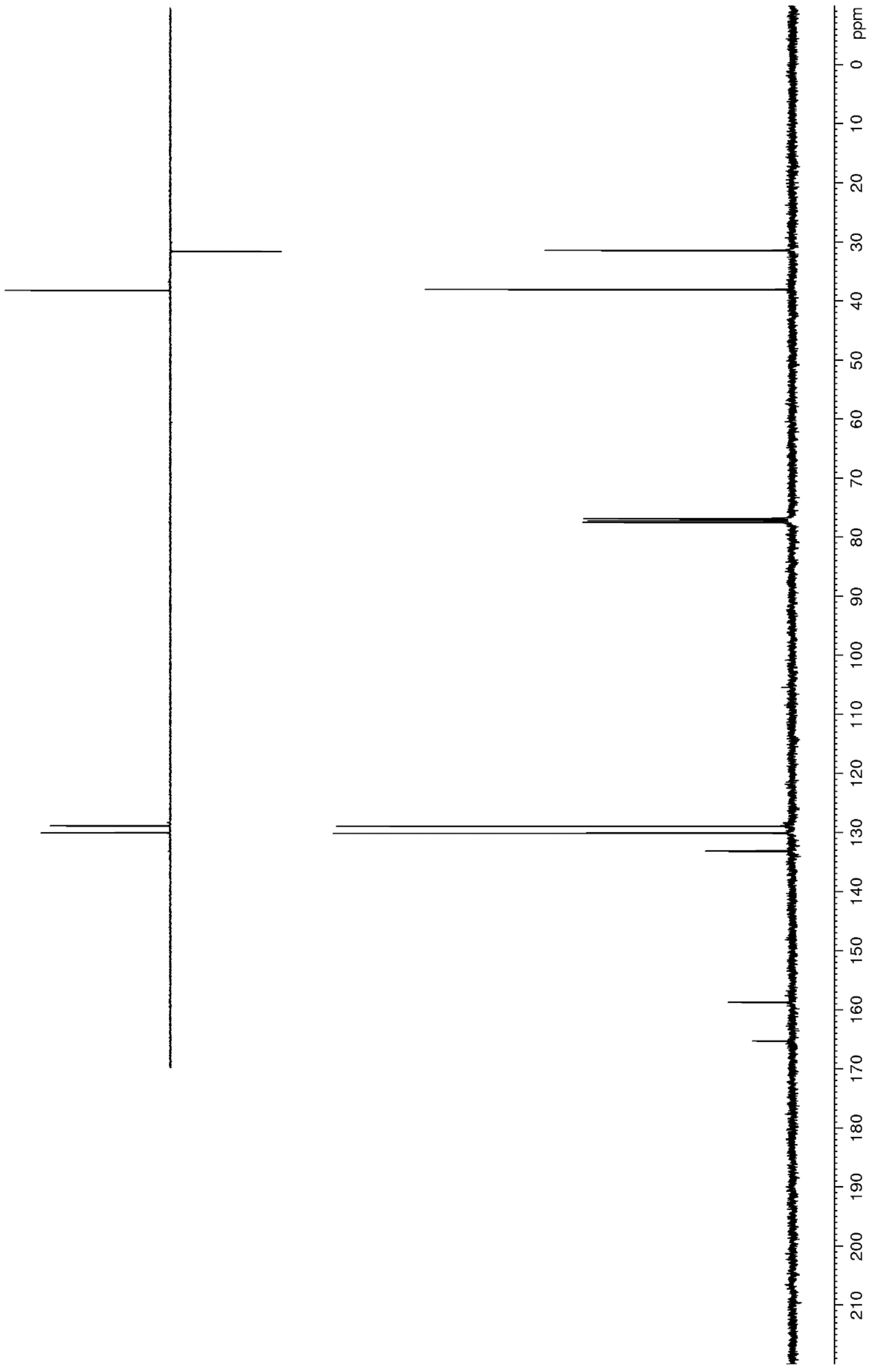

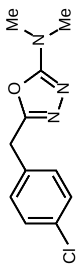

Figure 13. ^{1}H NMR (400 MHz, $CDCl_3$) of **4d**

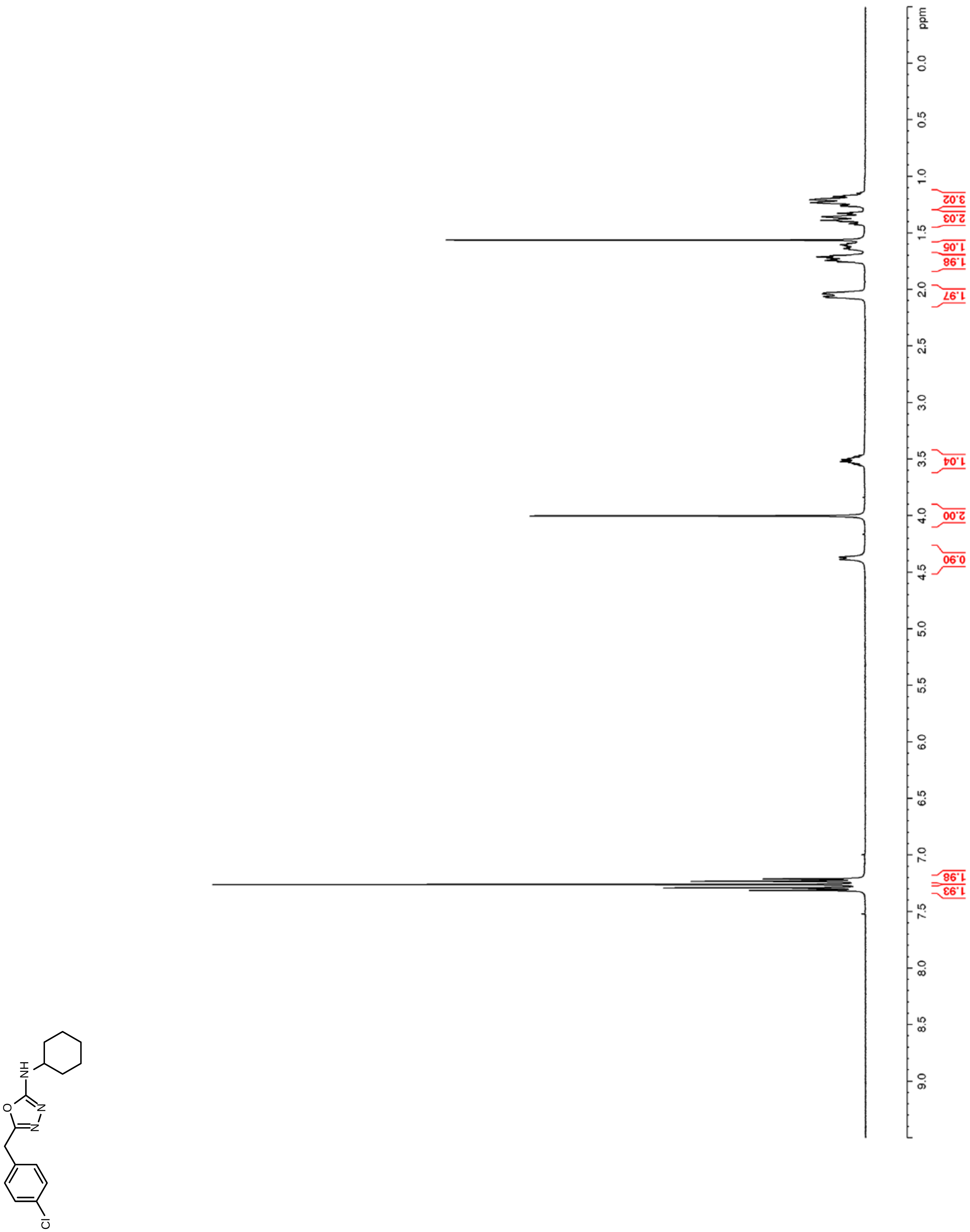

Figure 14. ^{13}C NMR (100 MHz, $CDCl_3$) of **4d**

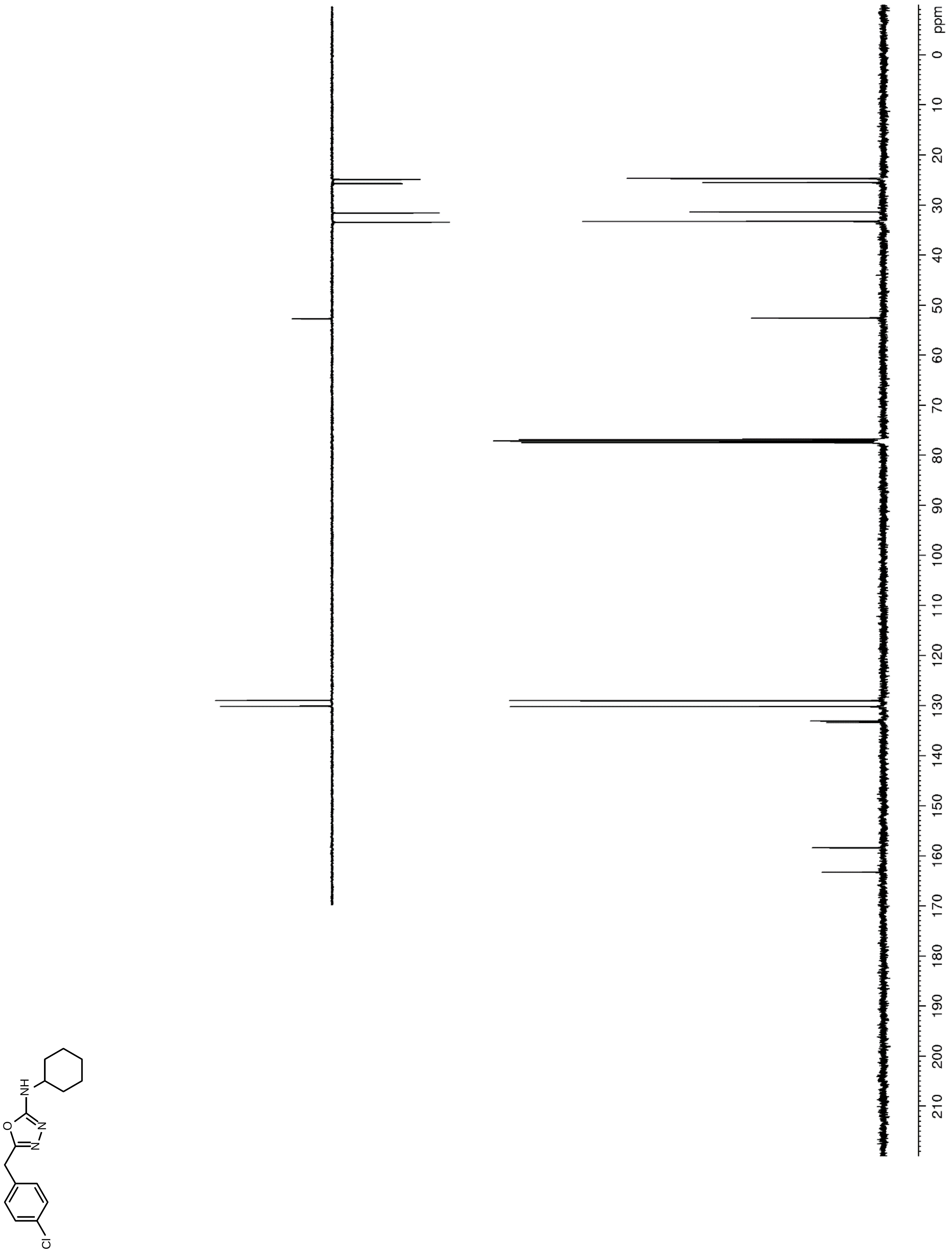

Figure 15. ^{1}H NMR (400 MHz, $CDCl_3$) of **4e**

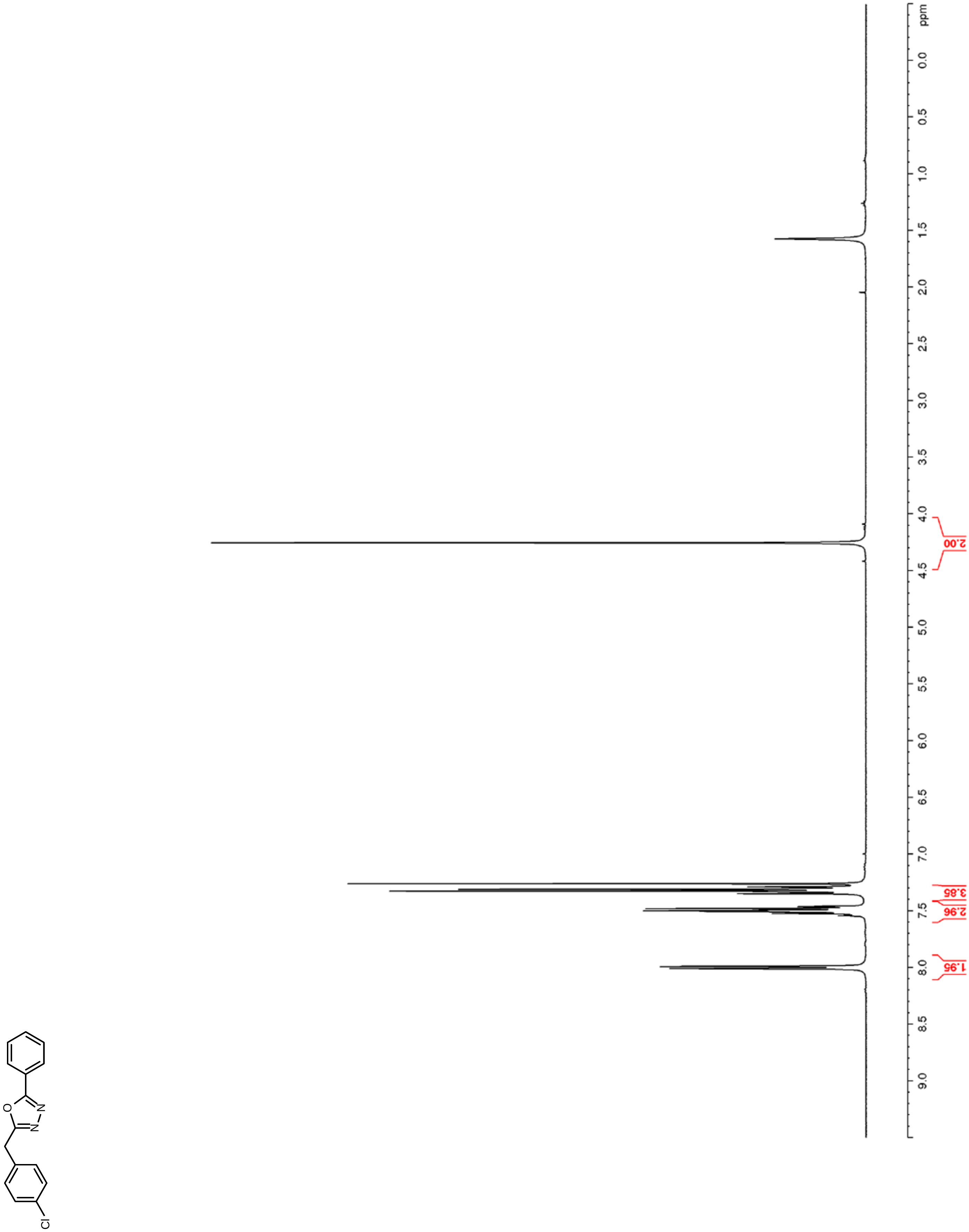

Figure 16. ^{13}C NMR (100 MHz, $CDCl_3$) of **4e**

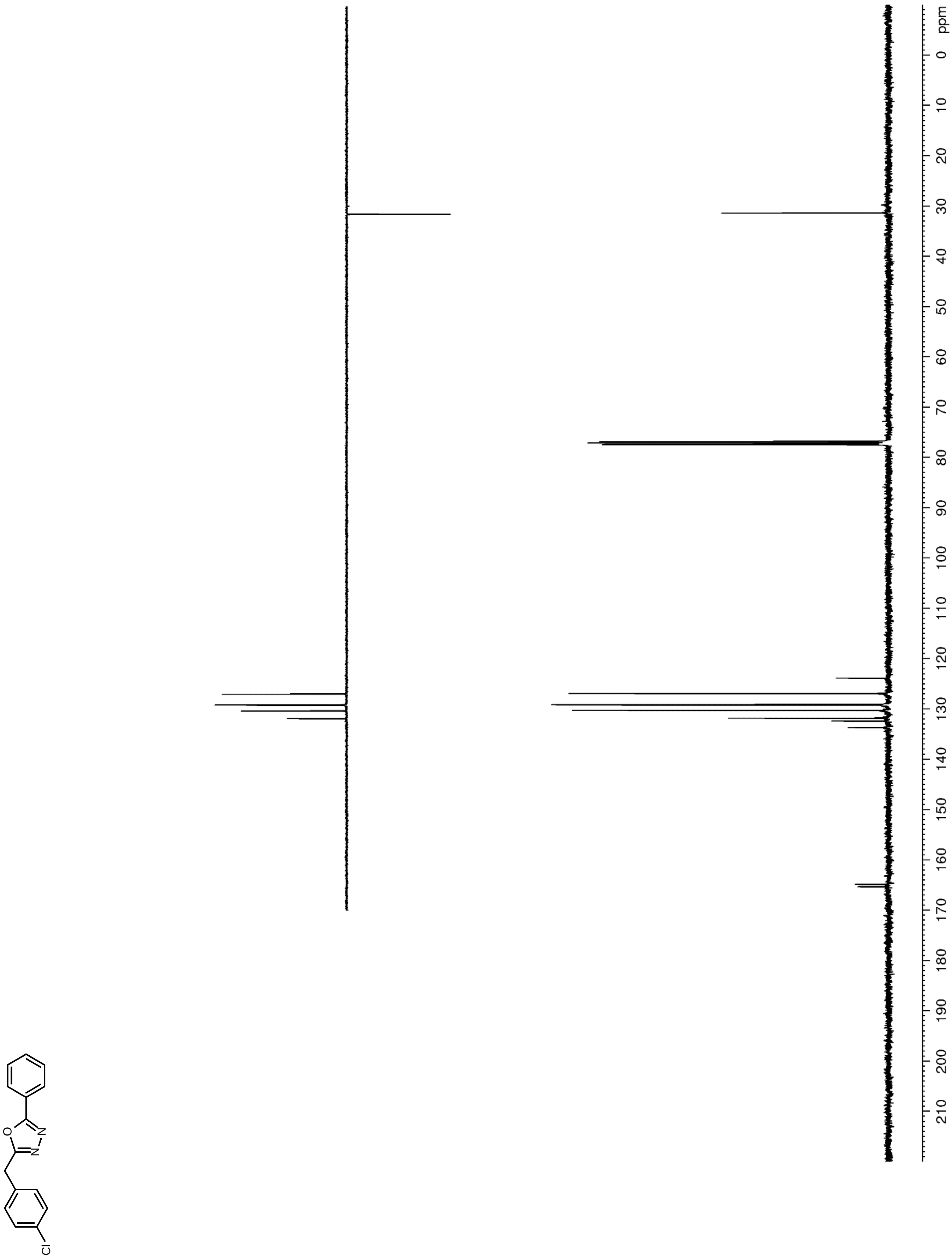

Figure 17. ^{1}H NMR (400 MHz, $CDCl_3$) of **4f**

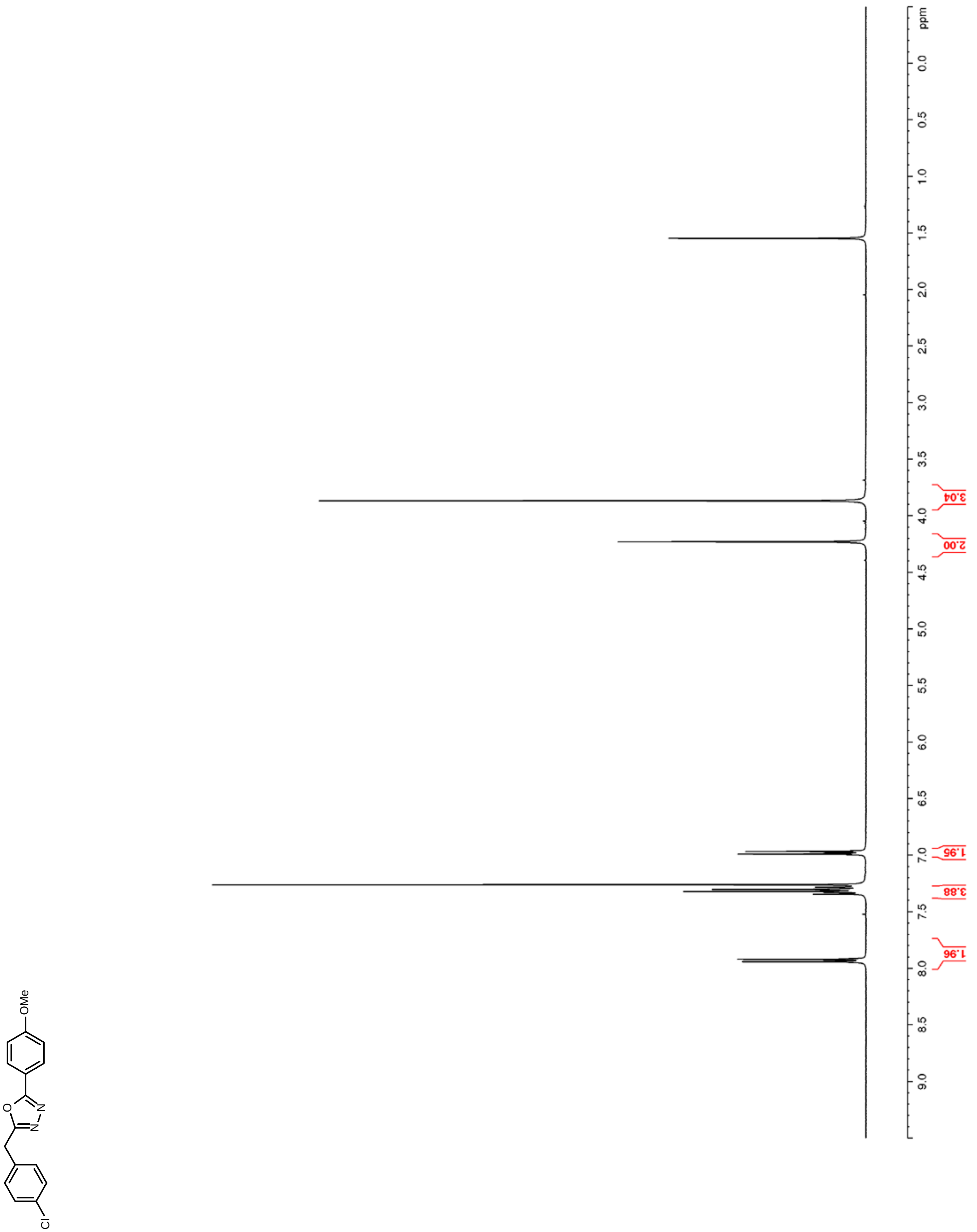

Figure 18. ^{13}C NMR (100 MHz, $CDCl_3$) of **4f**

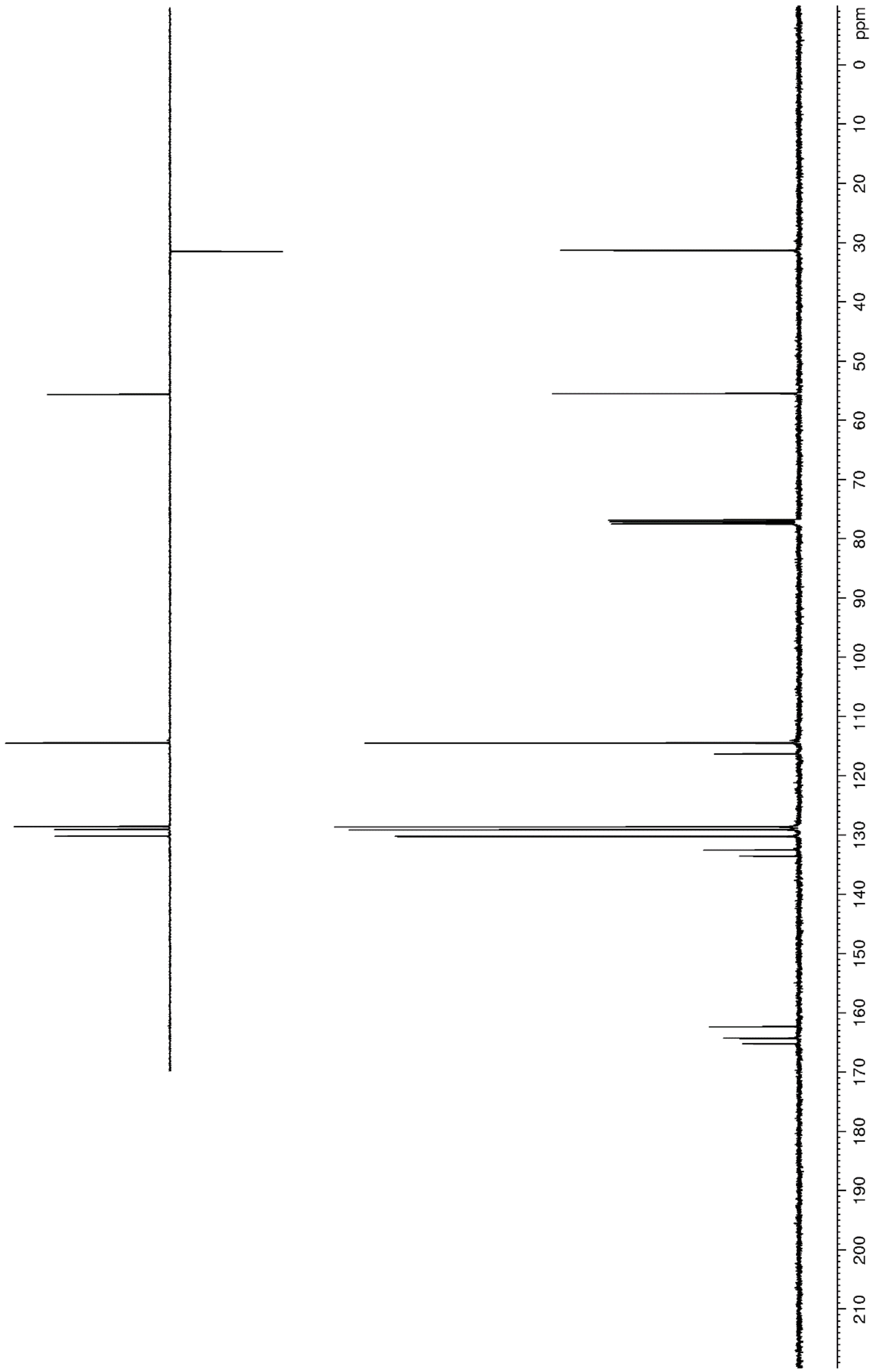

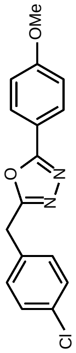

Figure 19. ^{1}H NMR (400 MHz, $CDCl_3$) of **4g**

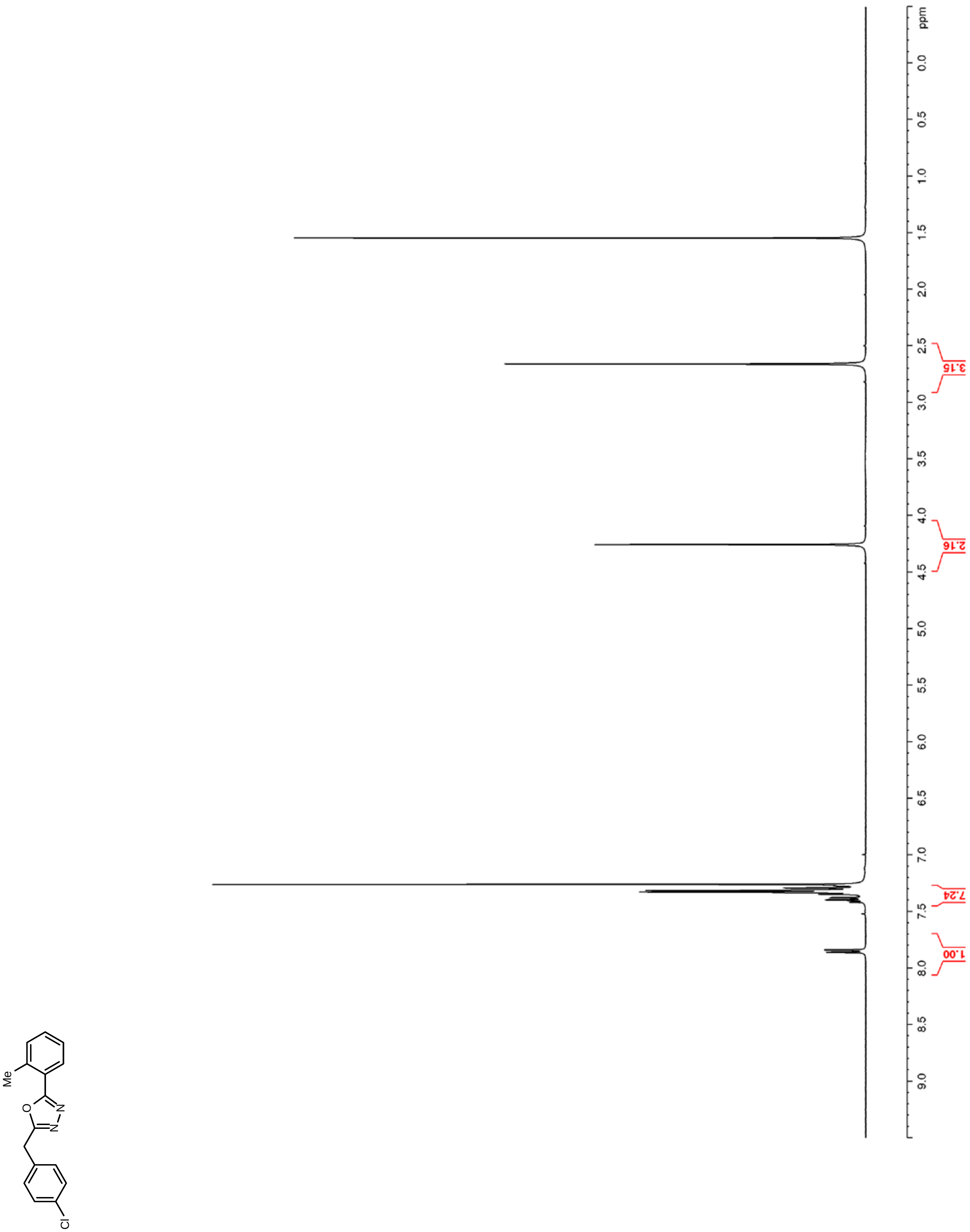

Figure 20. ^{13}C NMR (100 MHz, $CDCl_3$) of **4g**

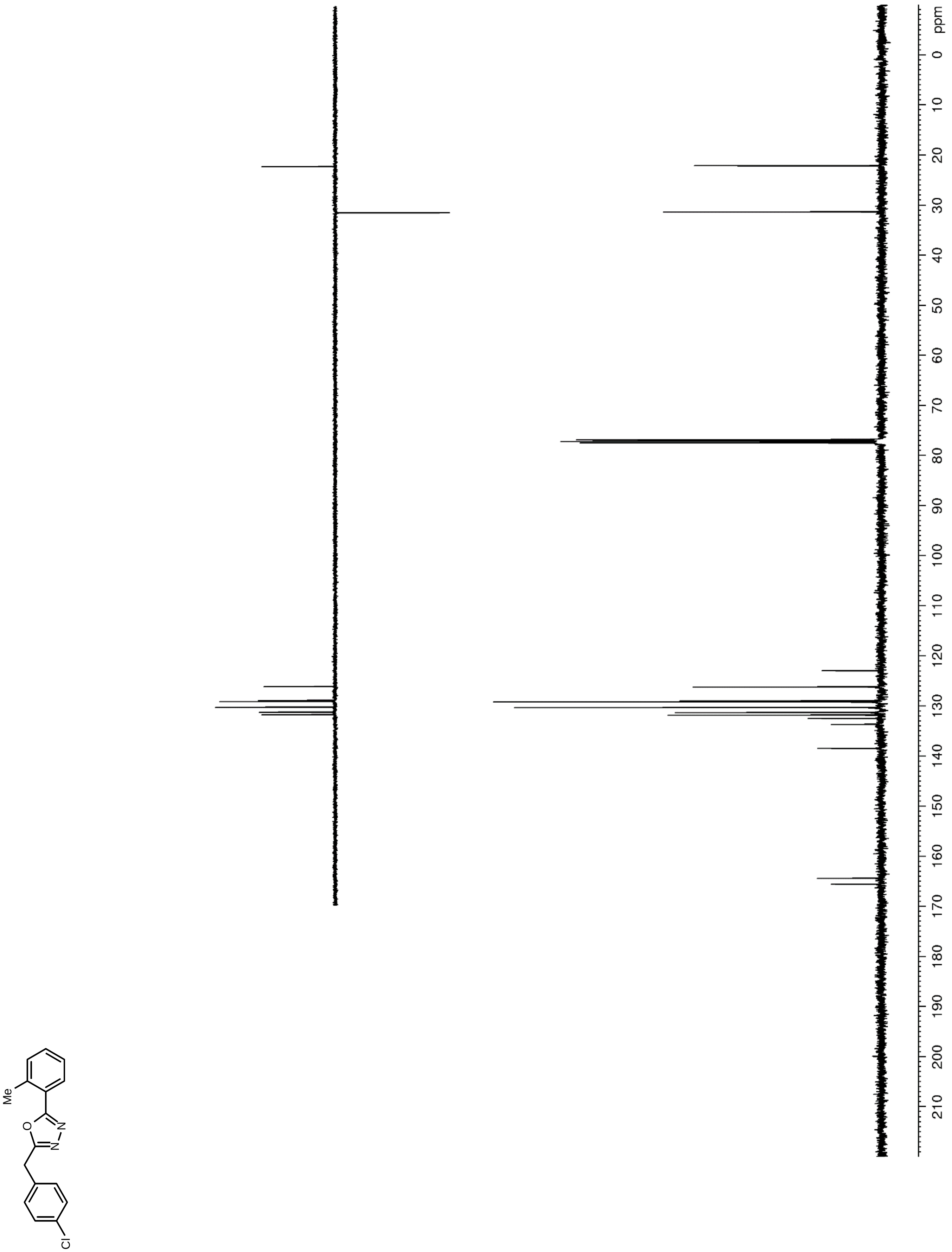

Figure 21. ^{1}H NMR (400 MHz, $CDCl_3$) of **4h**

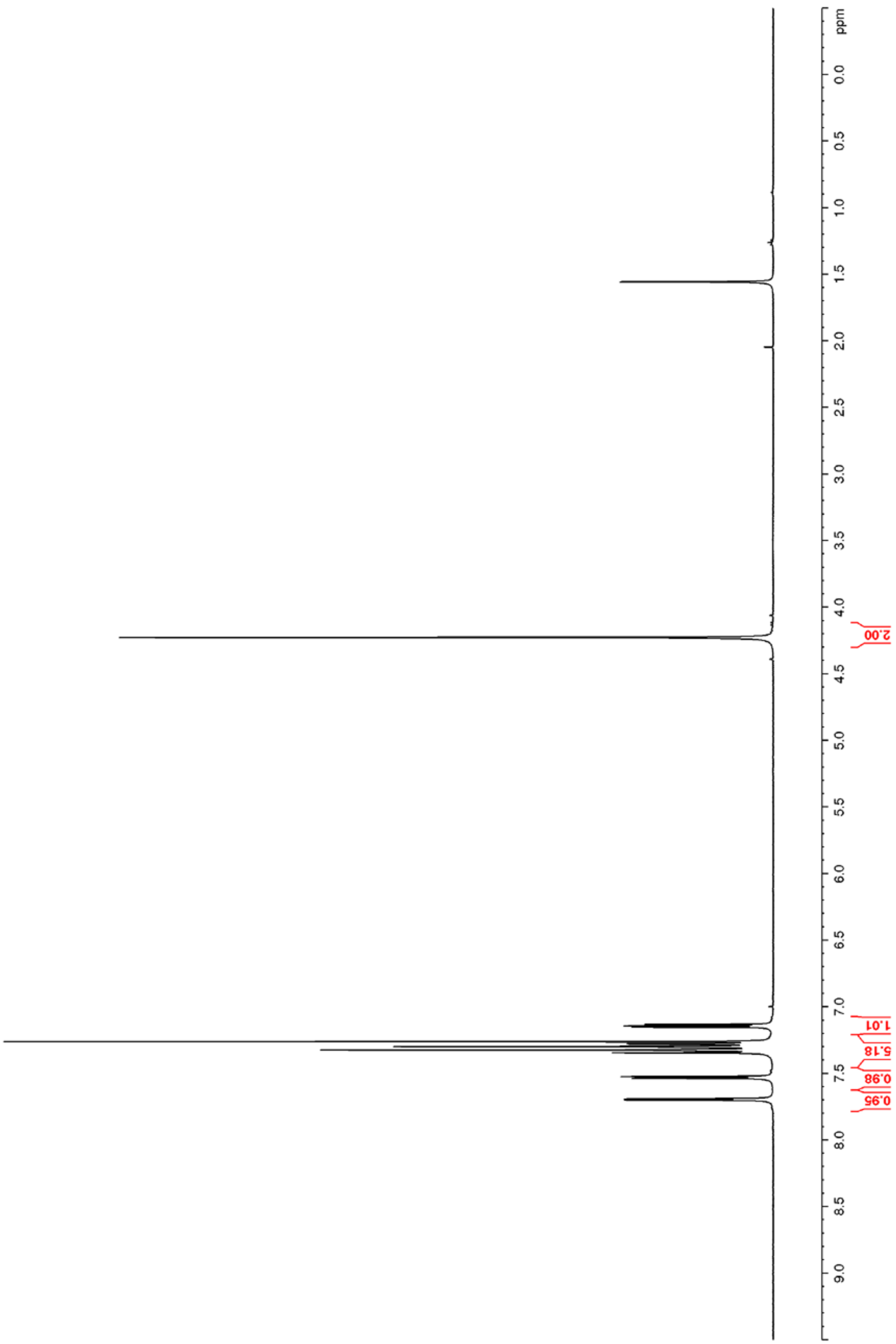

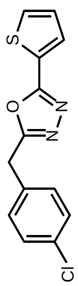

Figure 22. ^{13}C NMR (100 MHz, $CDCl_3$) of **4h**

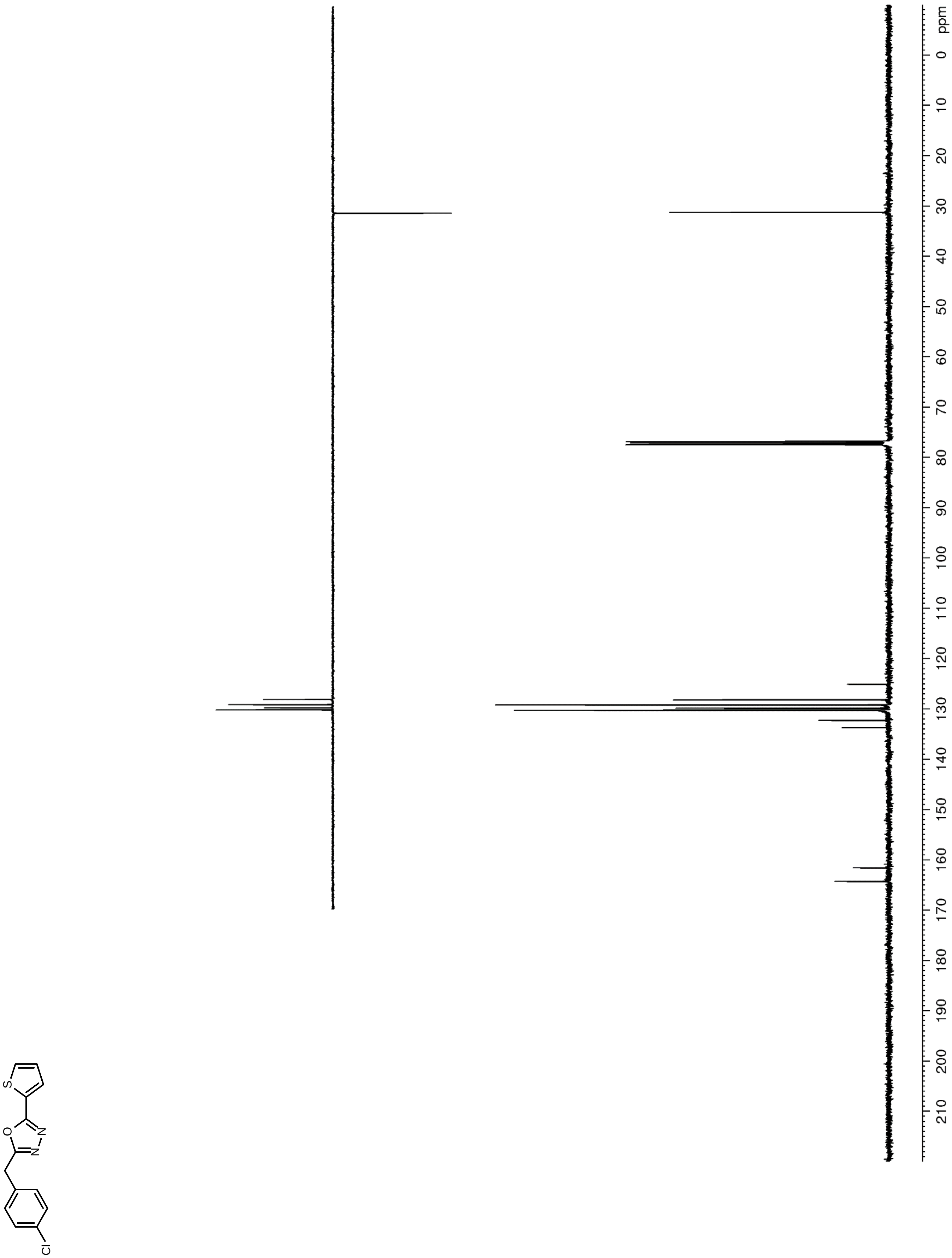

Figure 23. ^{1}H NMR (400 MHz, $CDCl_3$) of **4i**

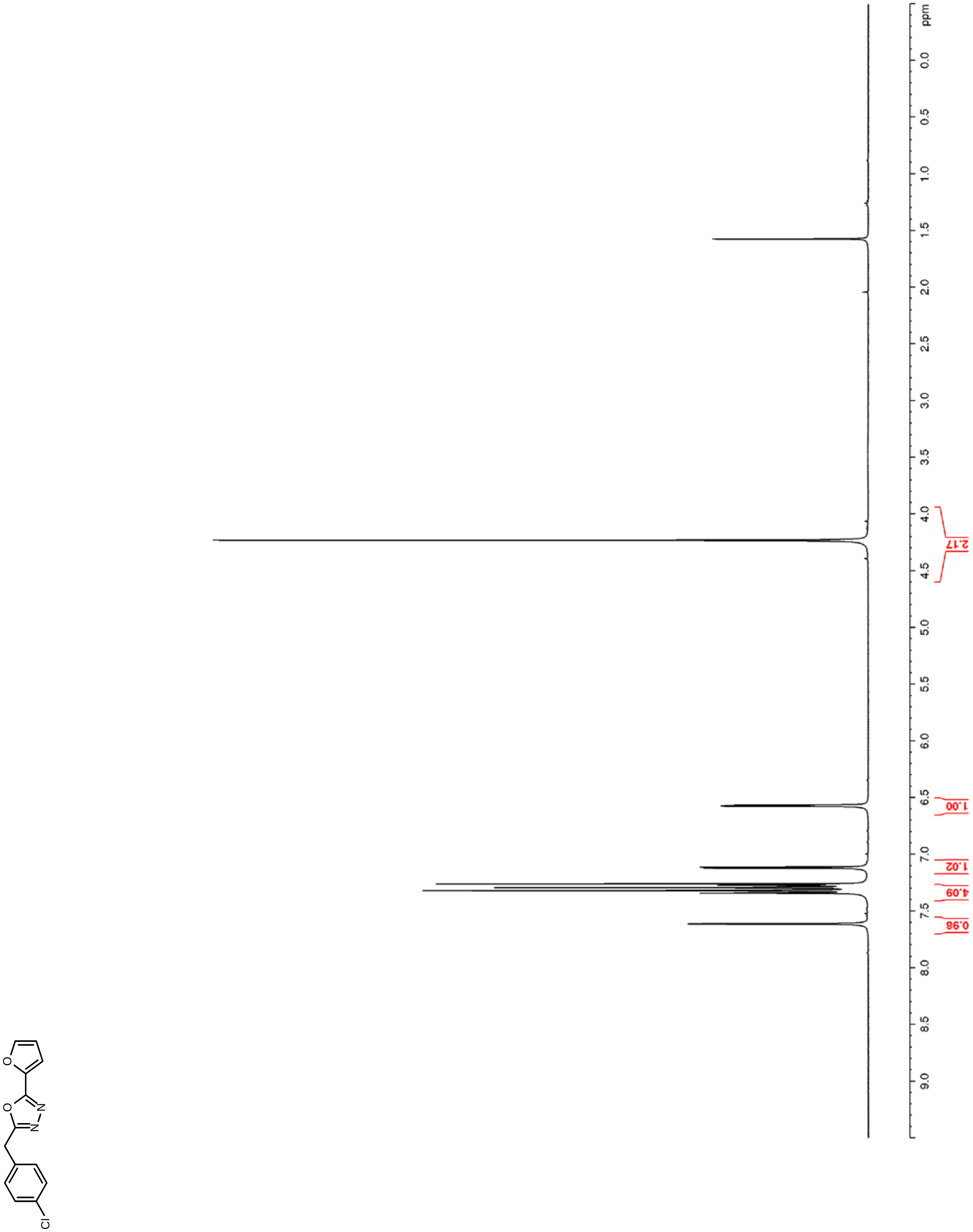

Figure 24. ^{13}C NMR (100 MHz, $CDCl_3$) of **4i**

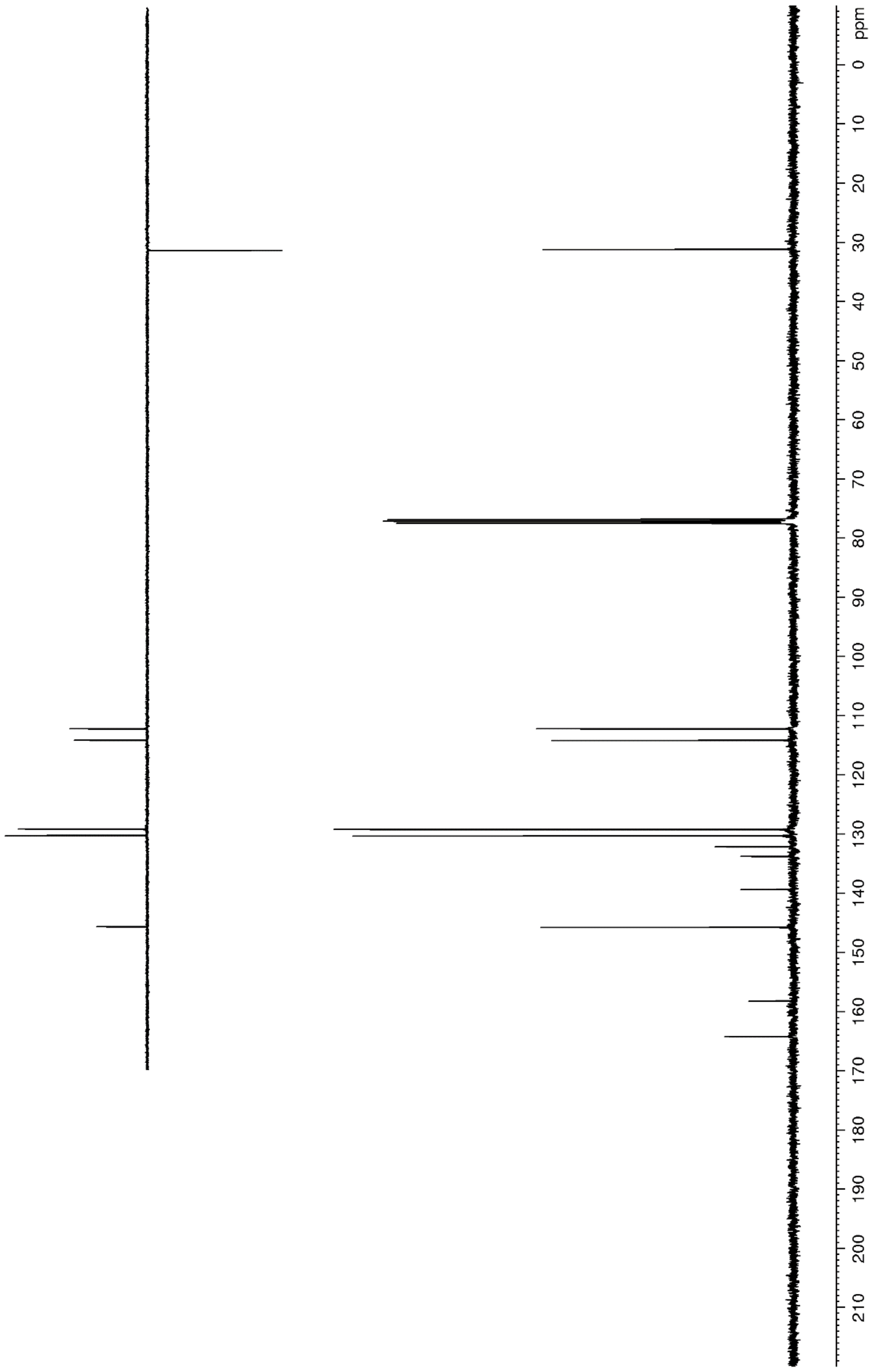

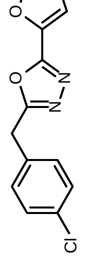

Figure 25. ^{1}H NMR (400 MHz, $CDCl_3$) of **4j**

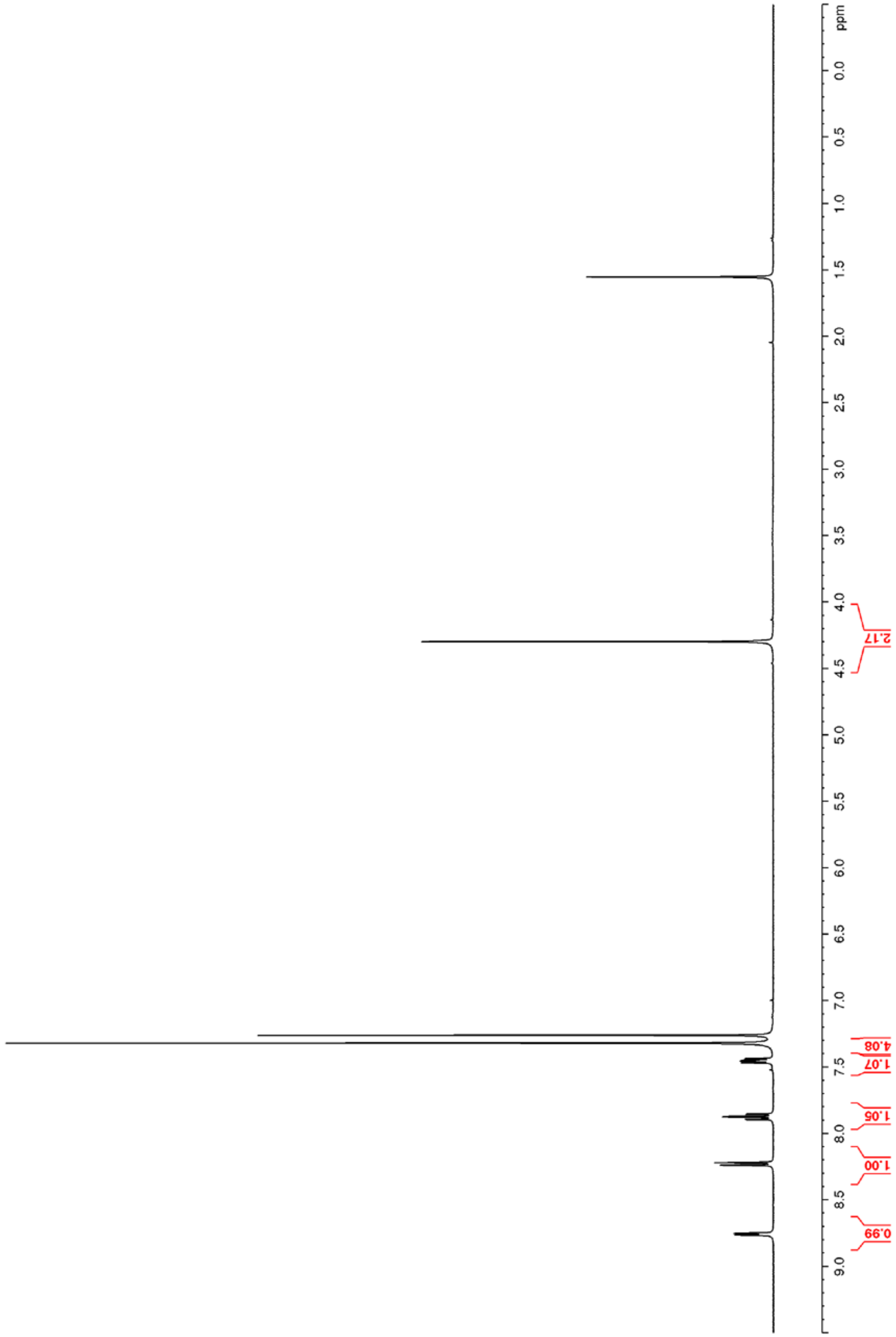

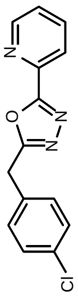

Figure 26. ^{13}C NMR (100 MHz, $CDCl_3$) of **4j**

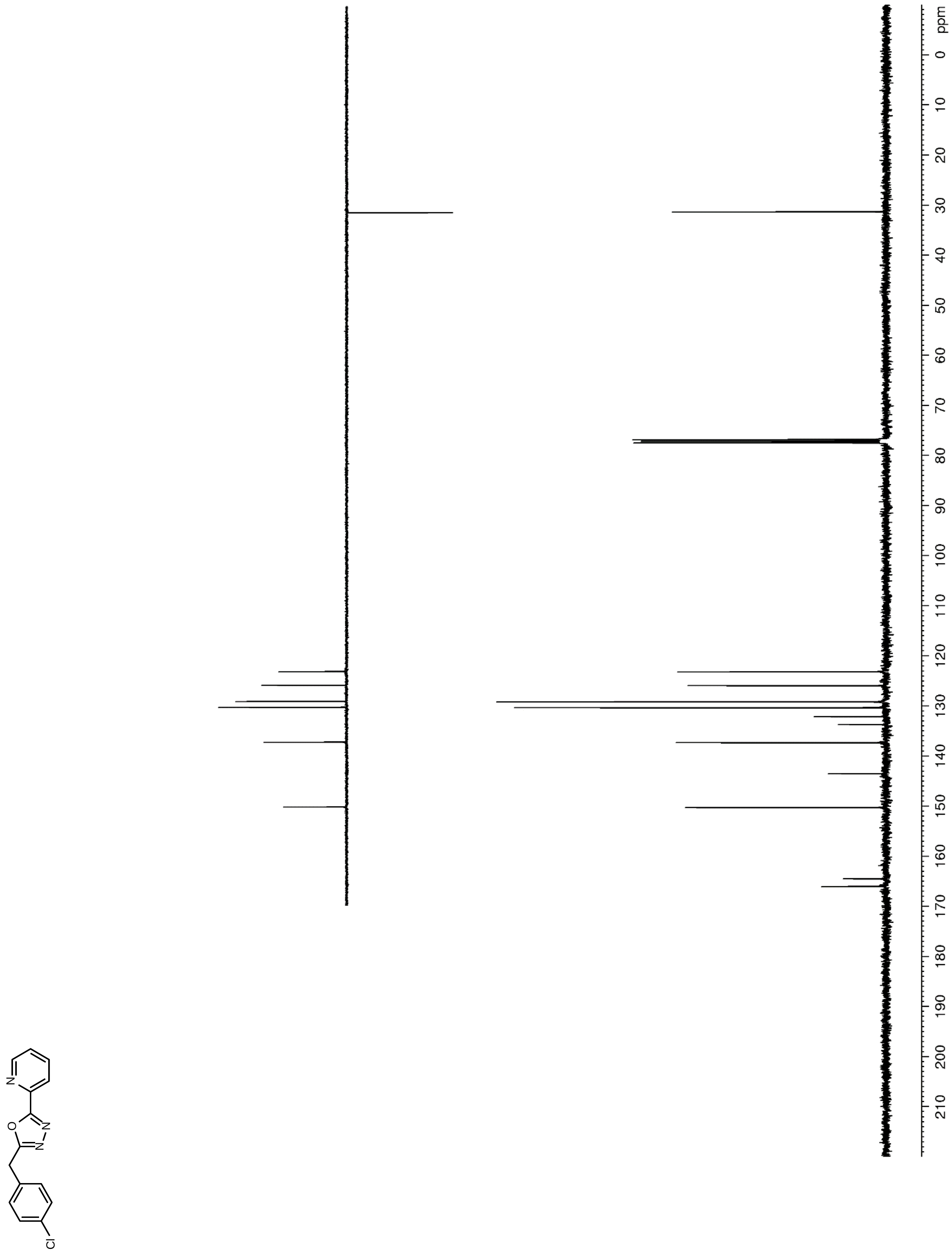

Figure 27. ^{1}H NMR (400 MHz, $CDCl_3$) of **4k**

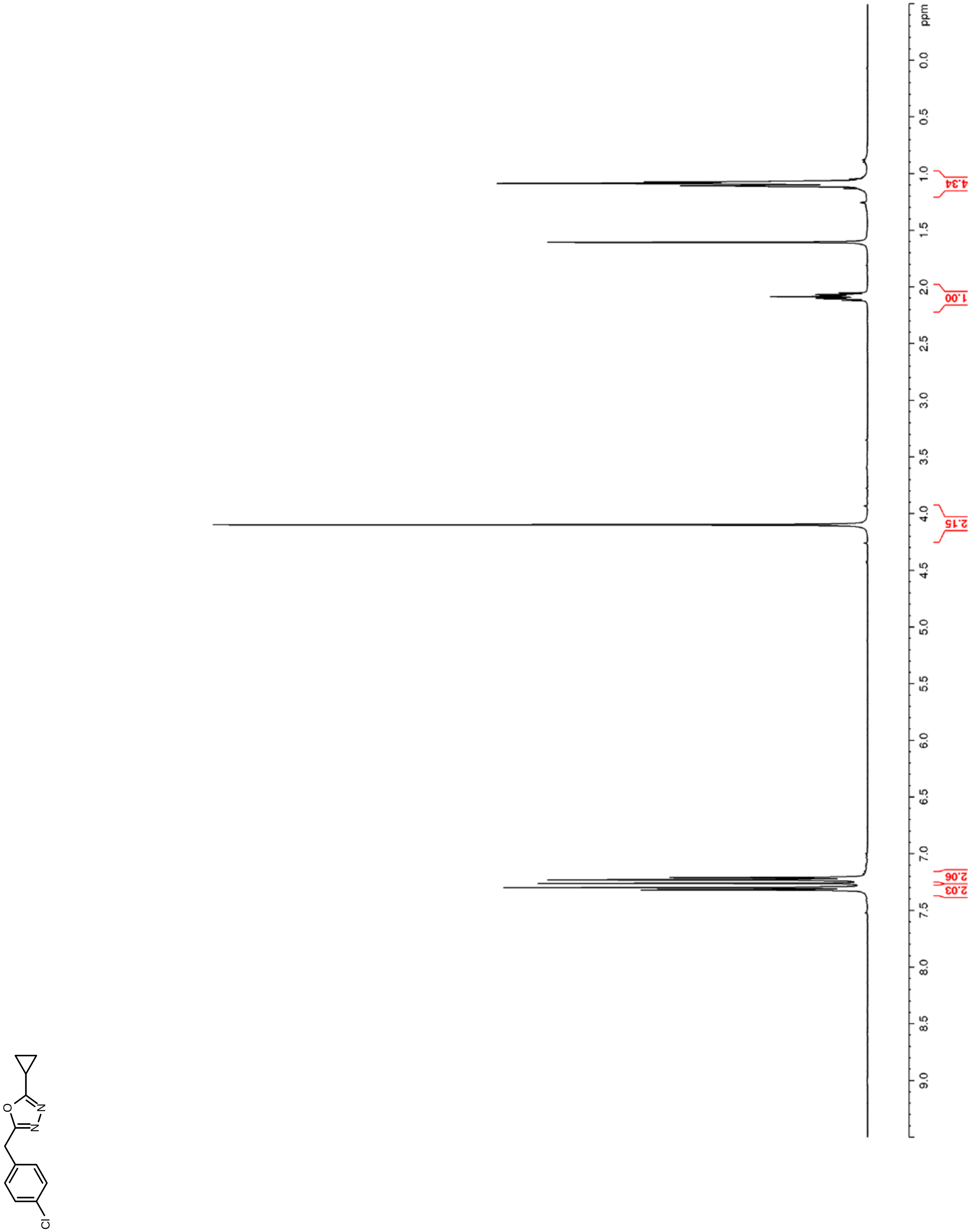

Figure 28. ^{13}C NMR (100 MHz, $CDCl_3$) of **4k**

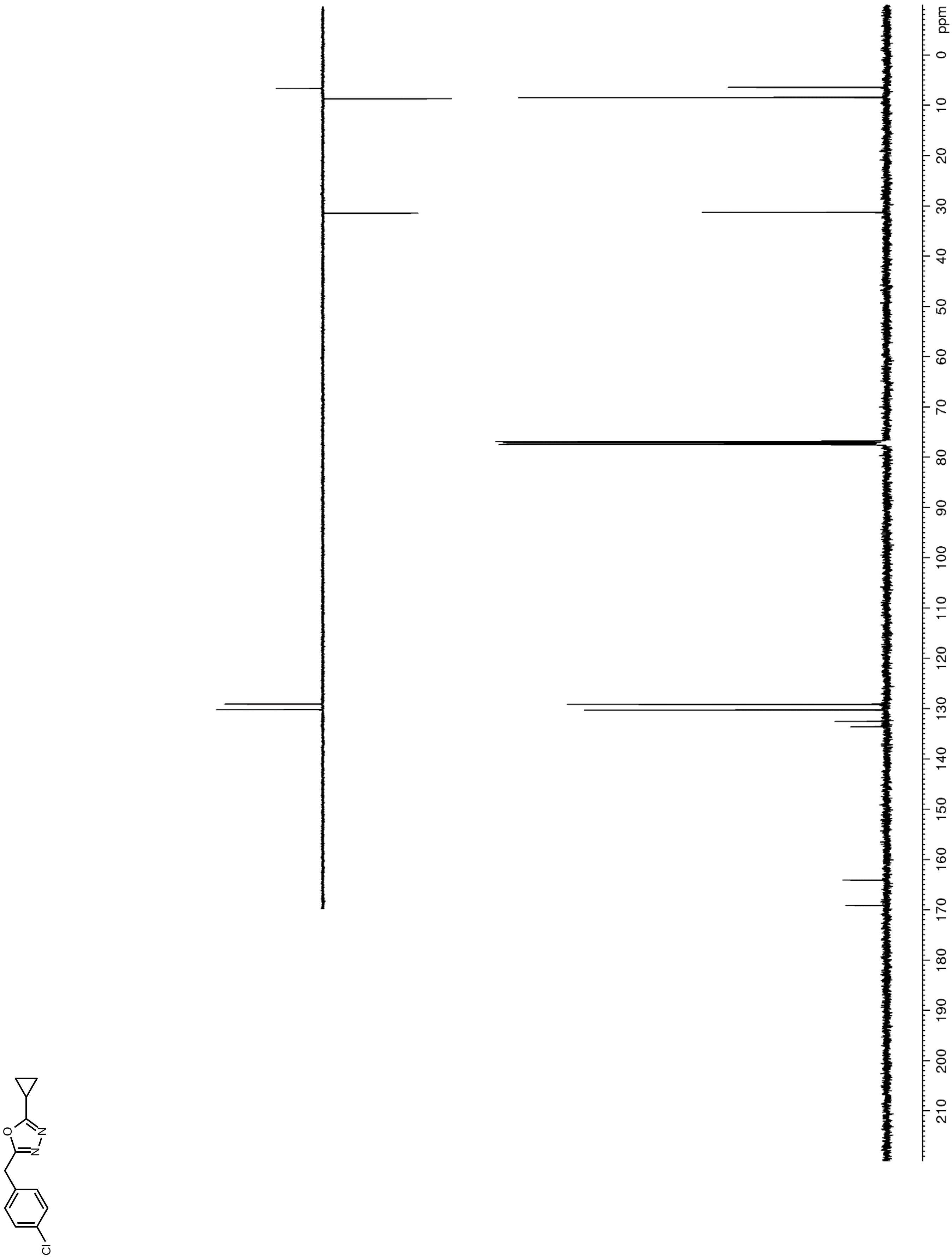

Figure 29. ^{1}H NMR (400 MHz, $CDCl_3$) of **4l**

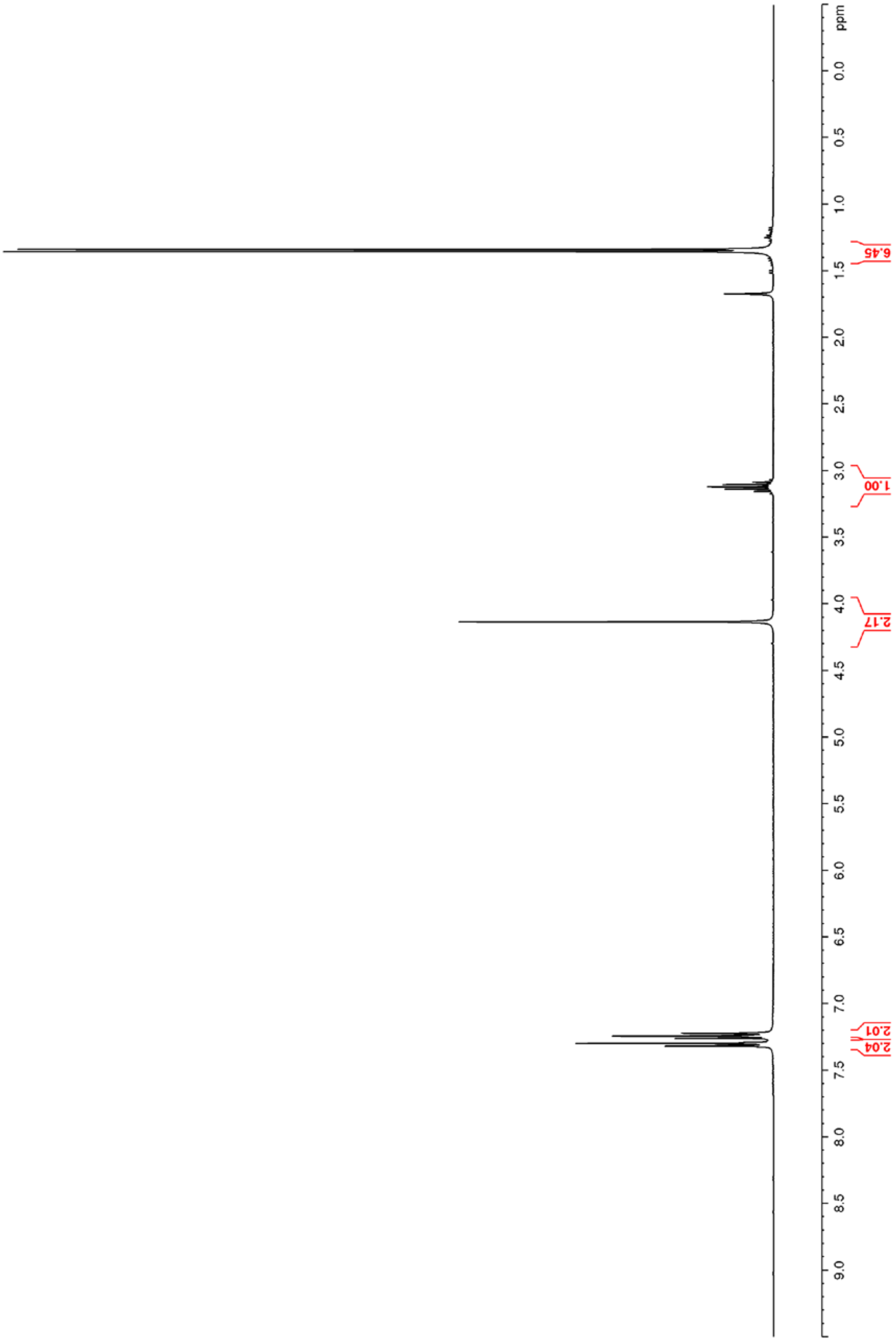

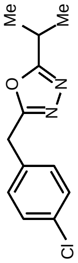

Figure 30. ^{13}C NMR (100 MHz, $CDCl_3$) of **4l**

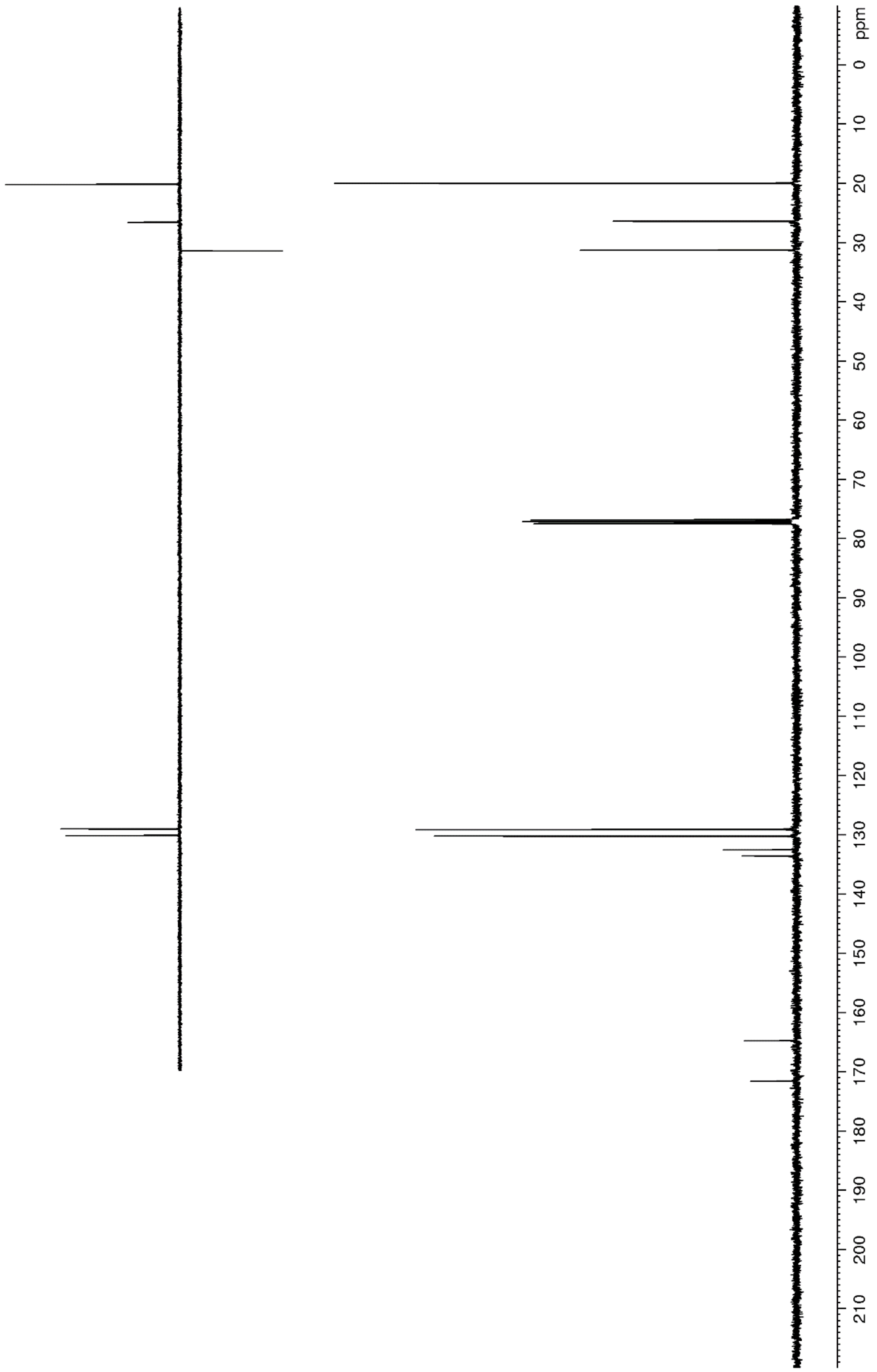

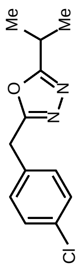

Figure 31. ^{1}H NMR (400 MHz, $CDCl_3$) of **4m**

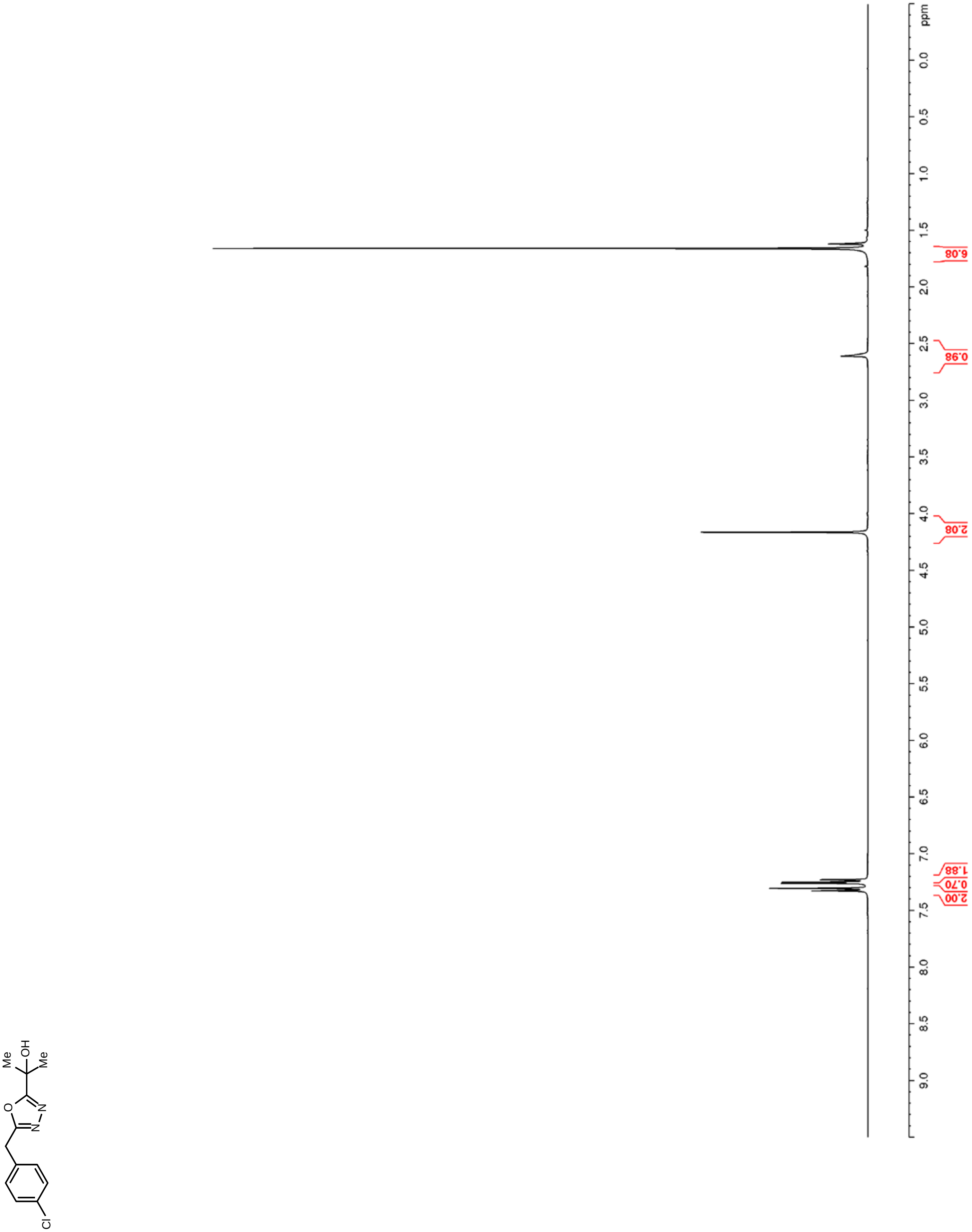

Figure 32. ^{13}C NMR (100 MHz, $CDCl_3$) of **4m**

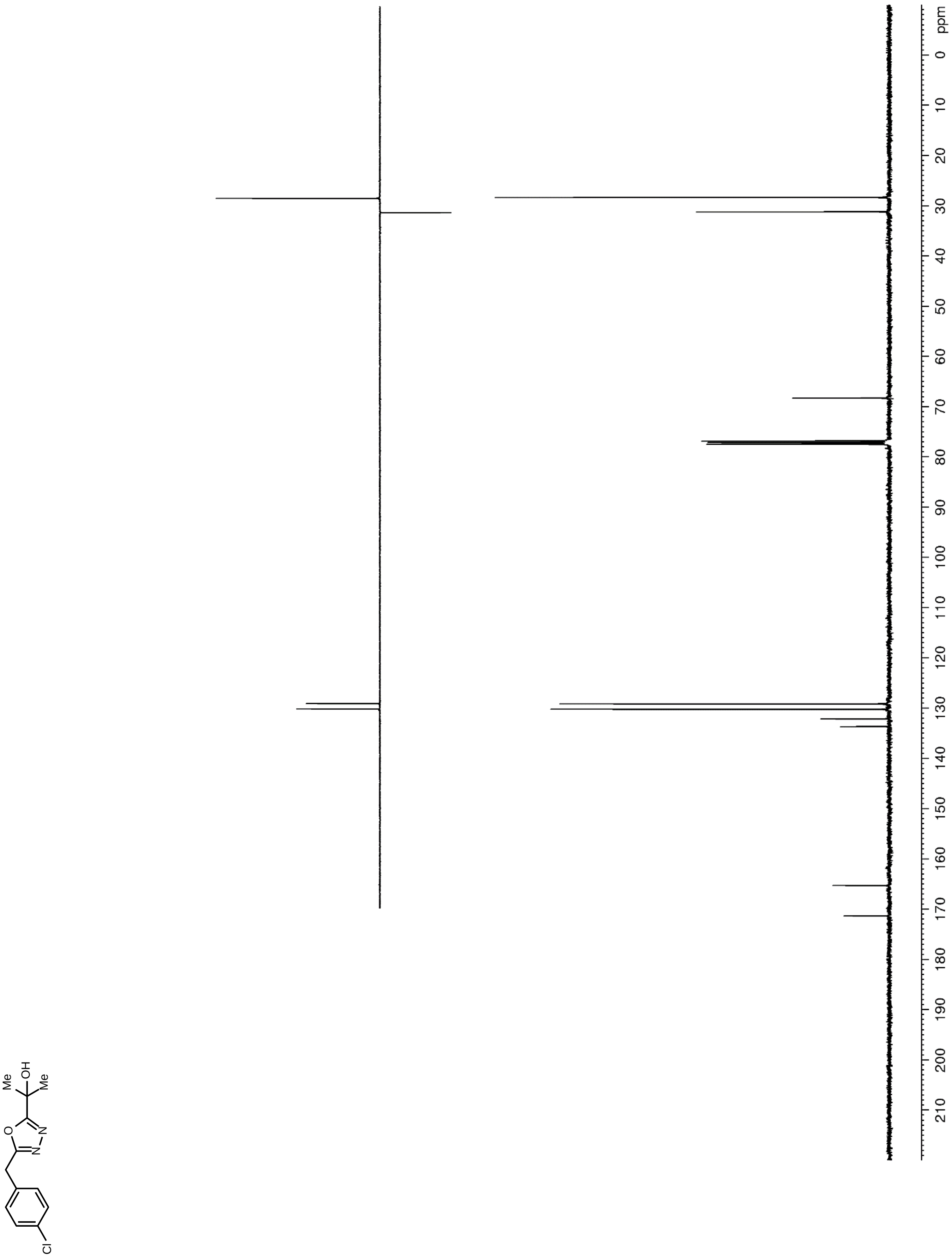

Figure 33. ¹H NMR (400 MHz, $CDCl_3$) of **9**

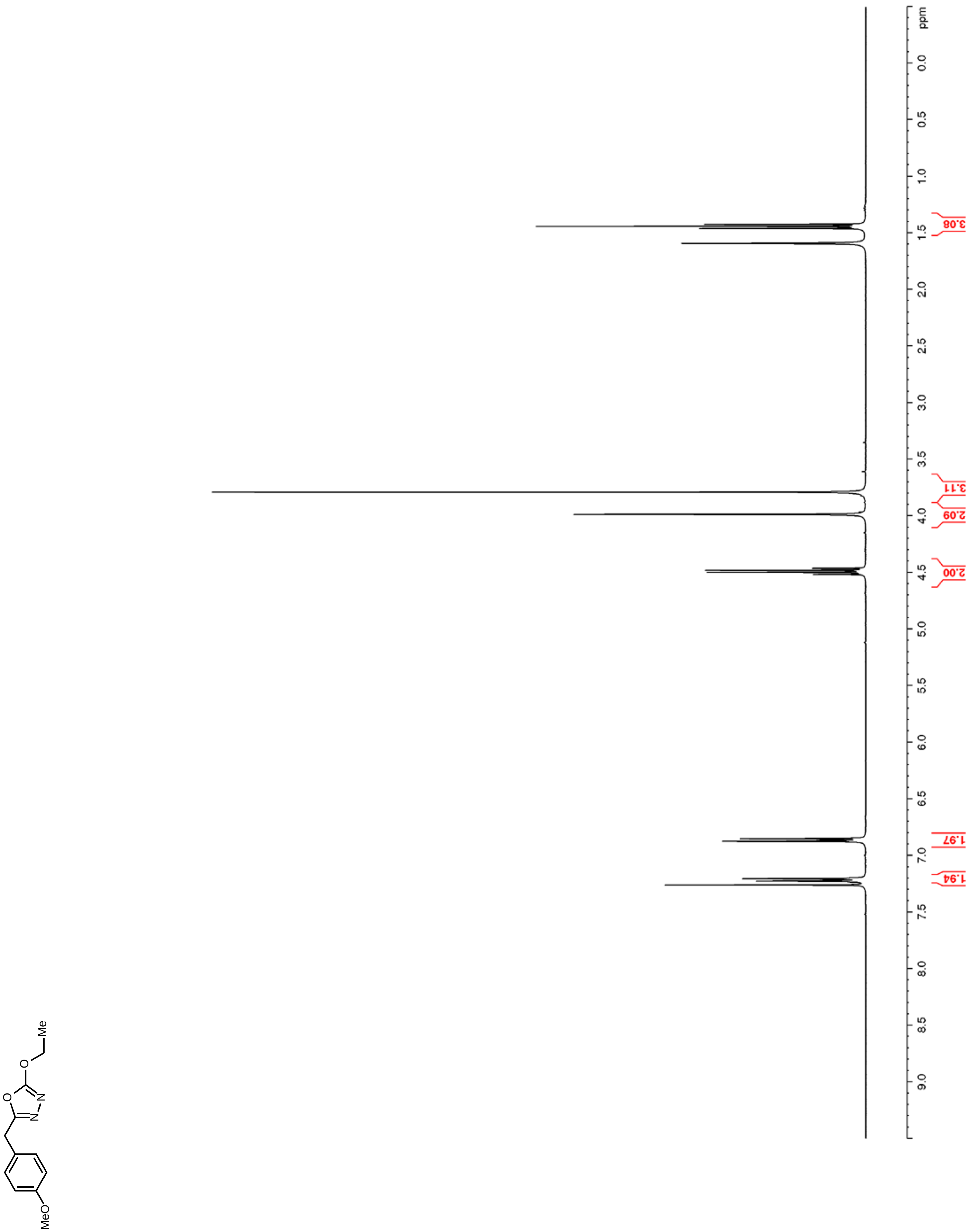

Figure 34. ^{13}C NMR (100 MHz, $CDCl_3$) of **9**

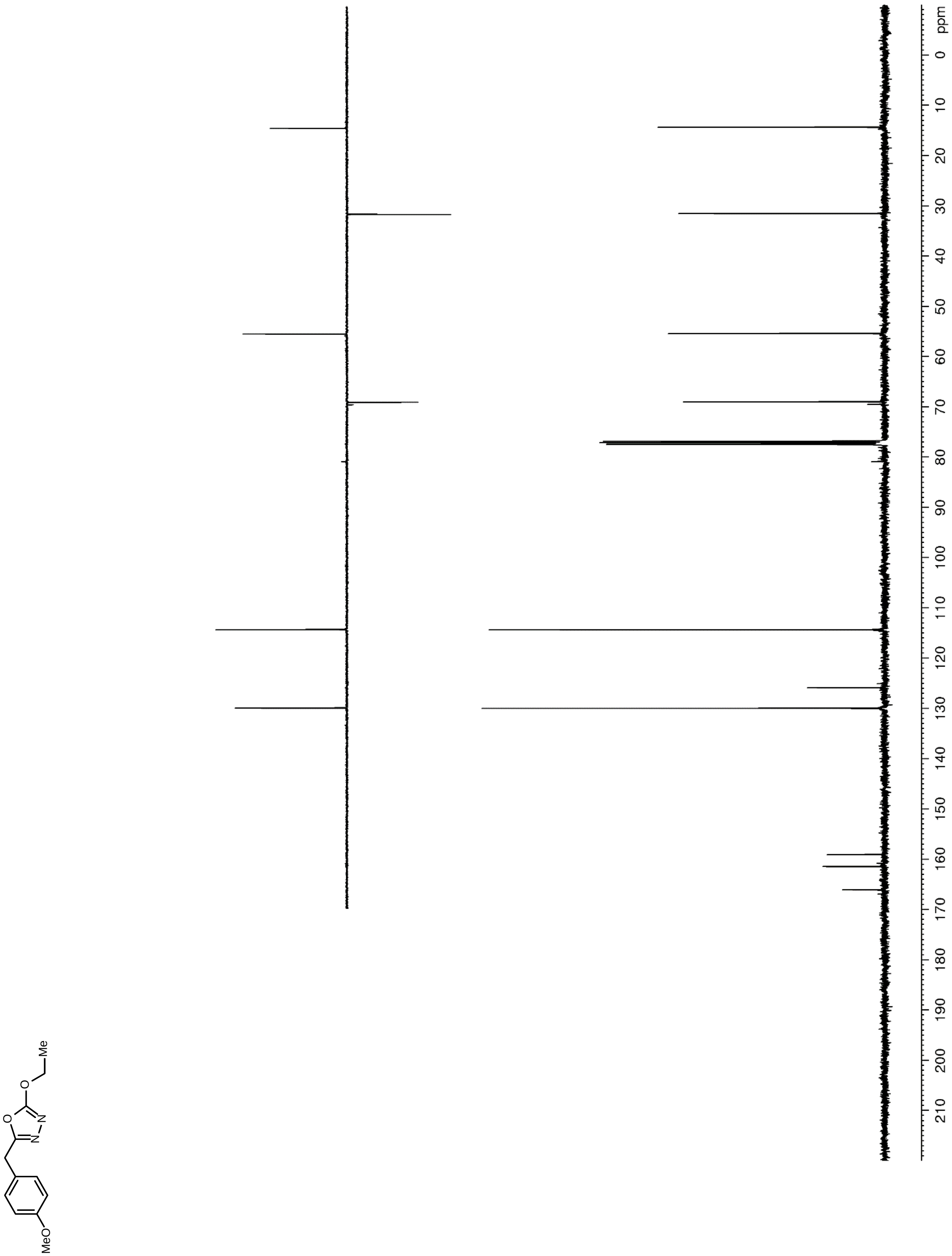

Figure 35. ^{1}H NMR (400 MHz, $CDCl_3$) of **10**

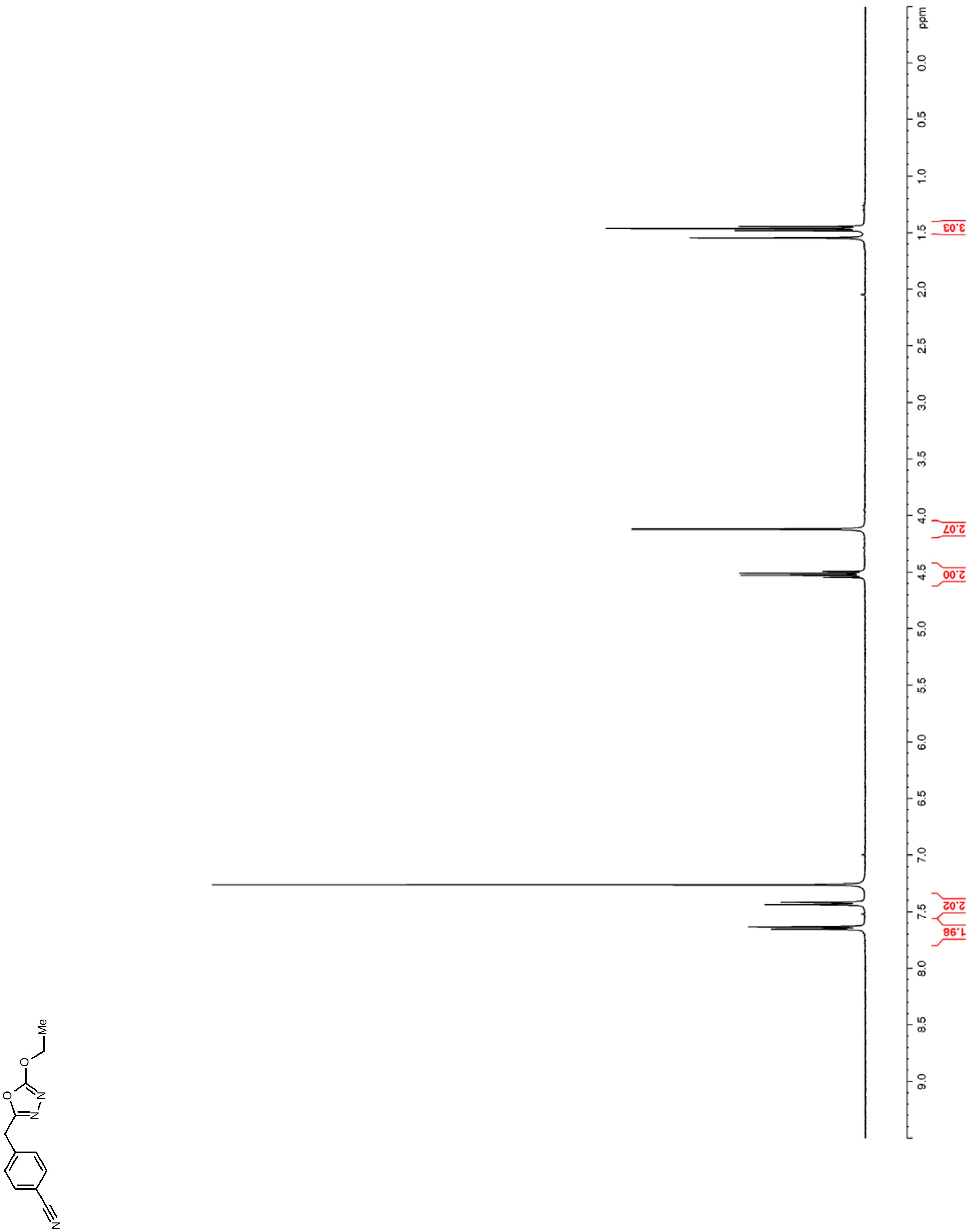

Figure 36. ^{13}C NMR (100 MHz, $CDCl_3$) of **10**

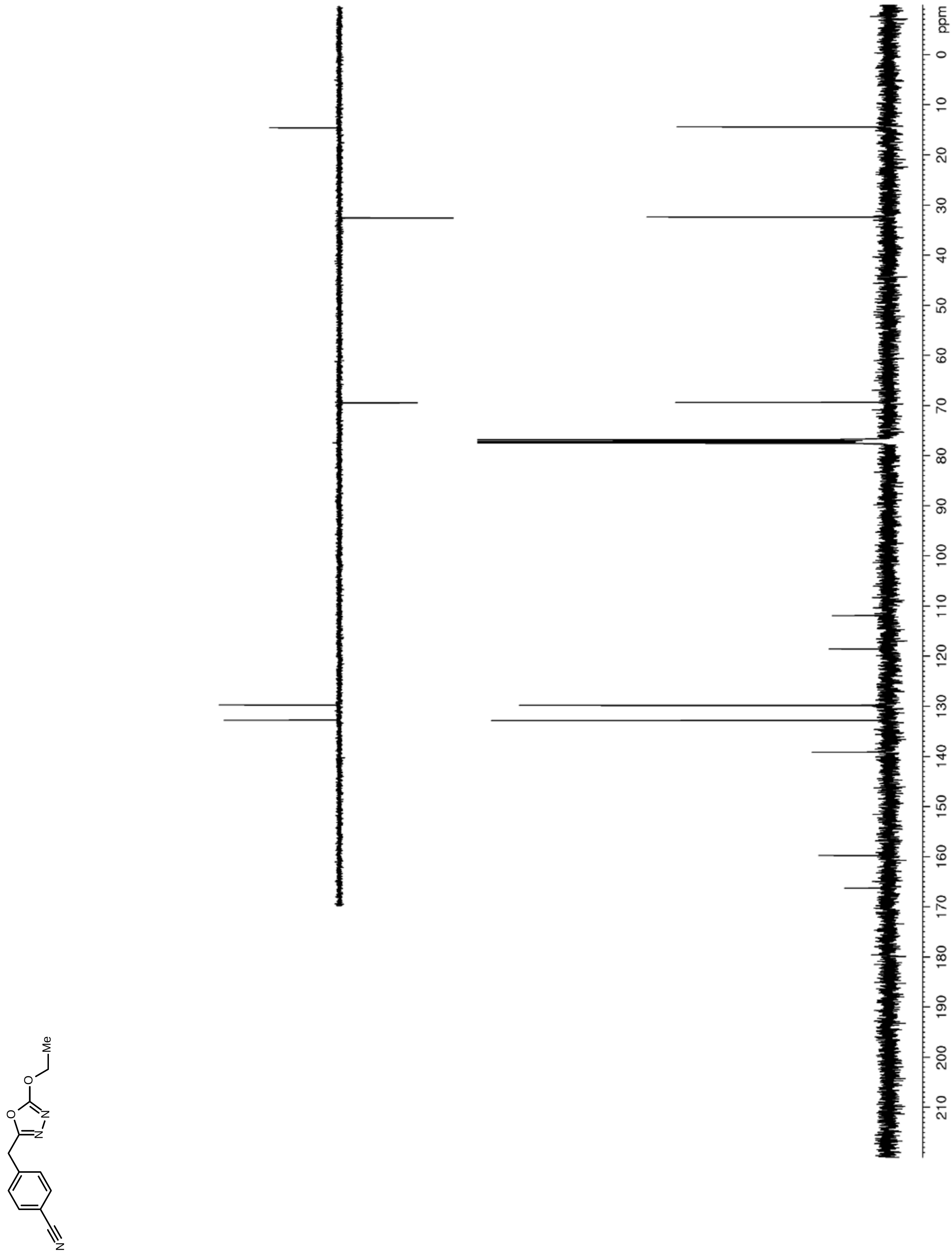

Figure 37. ^{1}H NMR (400 MHz, $CDCl_3$) of **11**

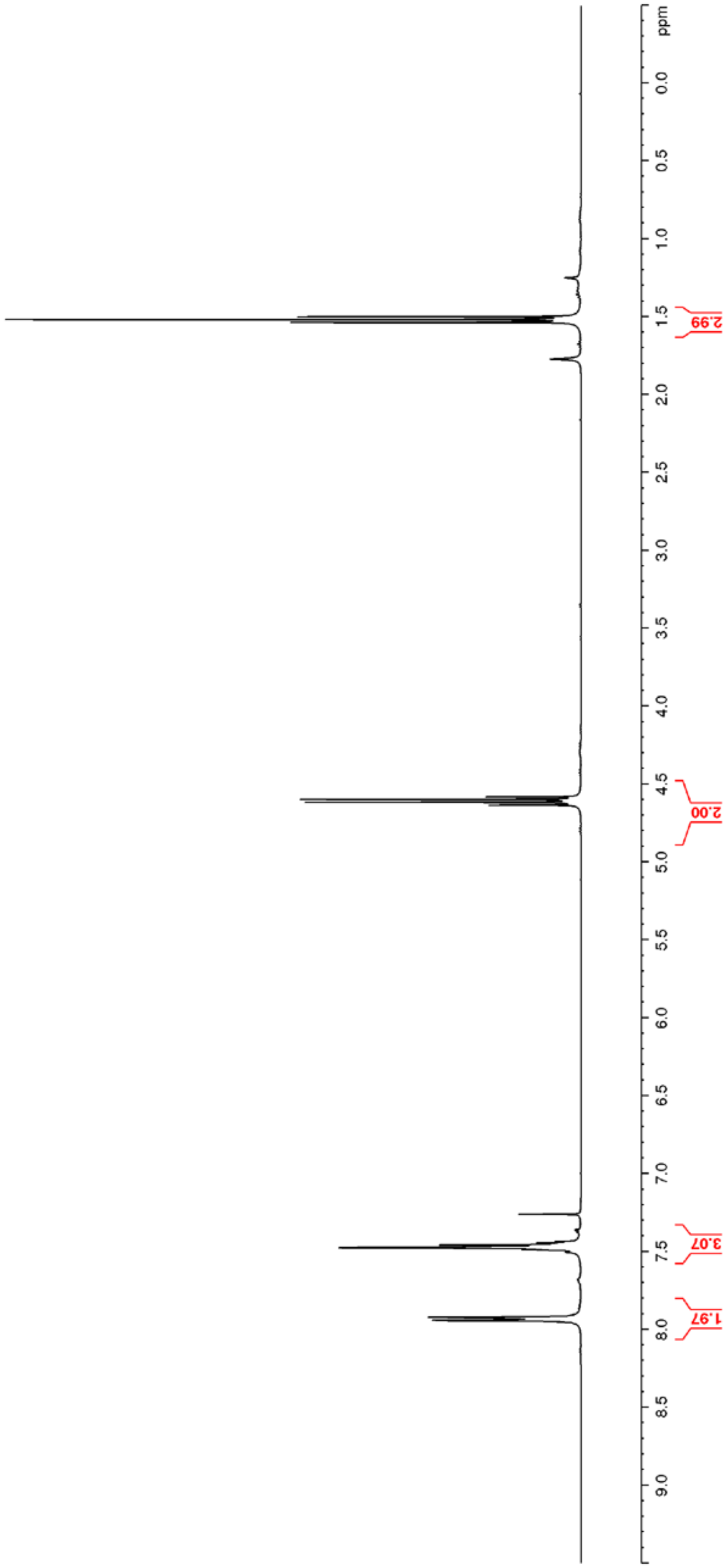

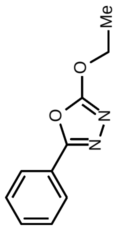

Figure 38. ^{13}C NMR (100 MHz, $CDCl_3$) of **11**

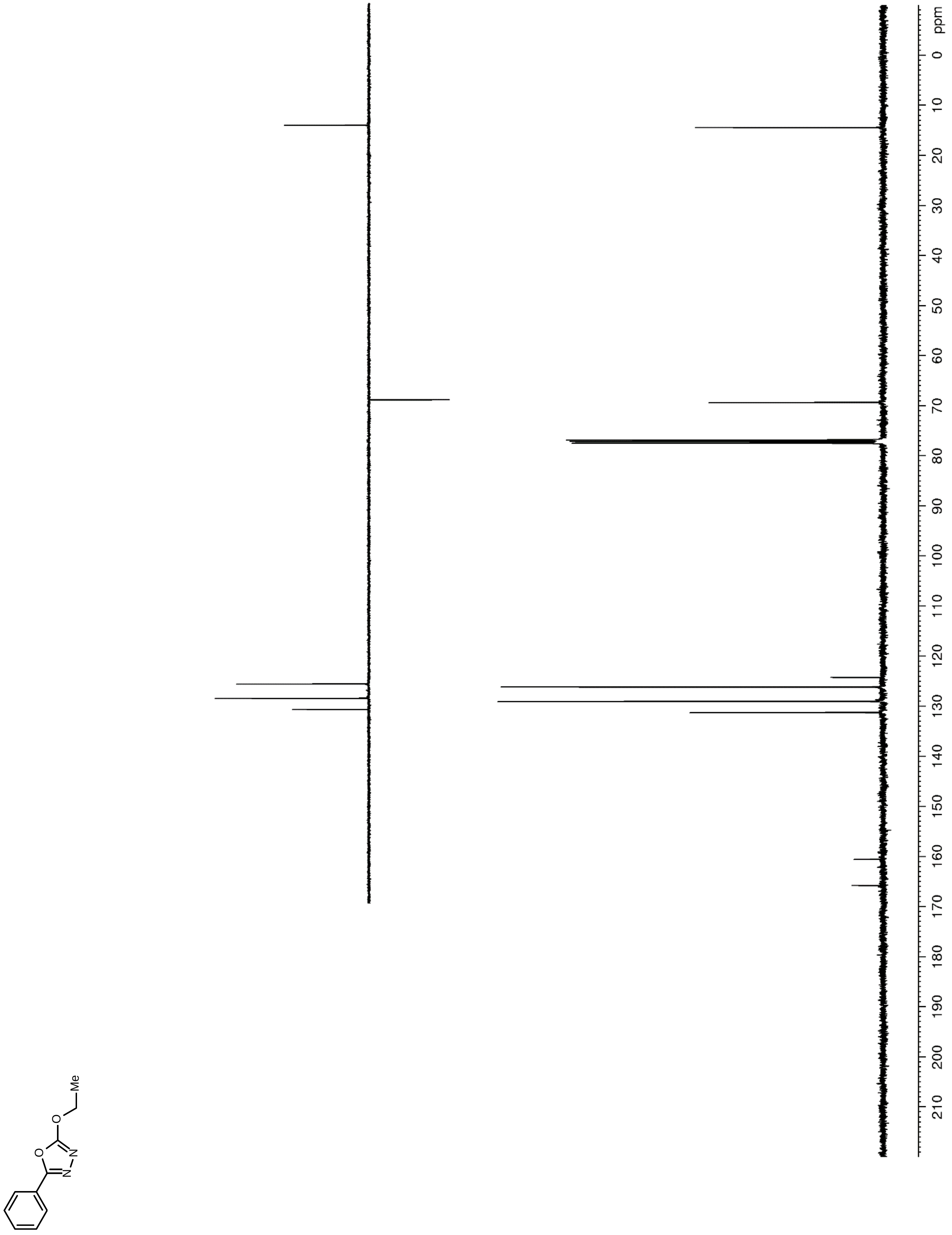

Figure 39. ^{1}H NMR (400 MHz, $CDCl_3$) of **12**

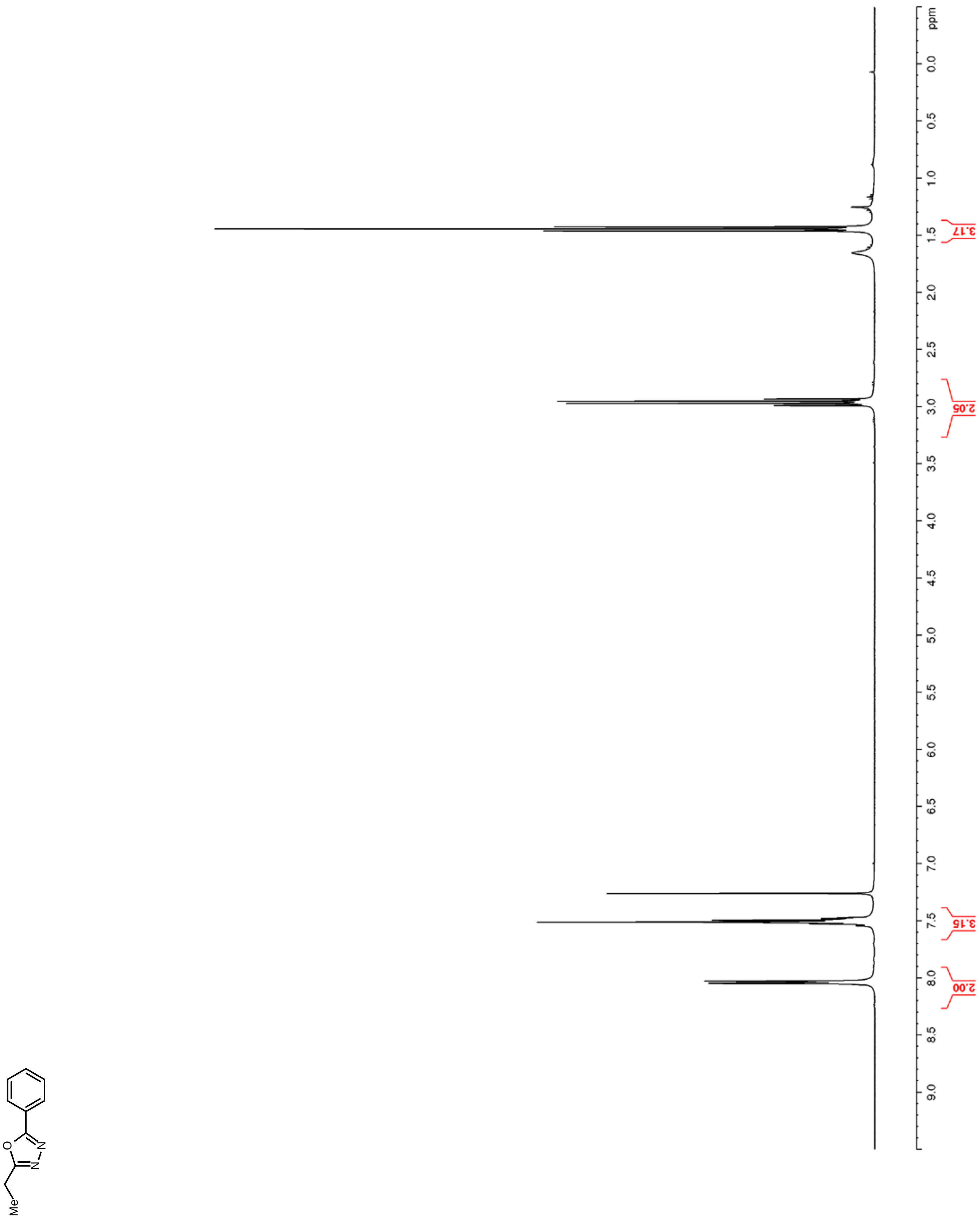

Figure 40. ^{13}C NMR (100 MHz, $CDCl_3$) of **12**

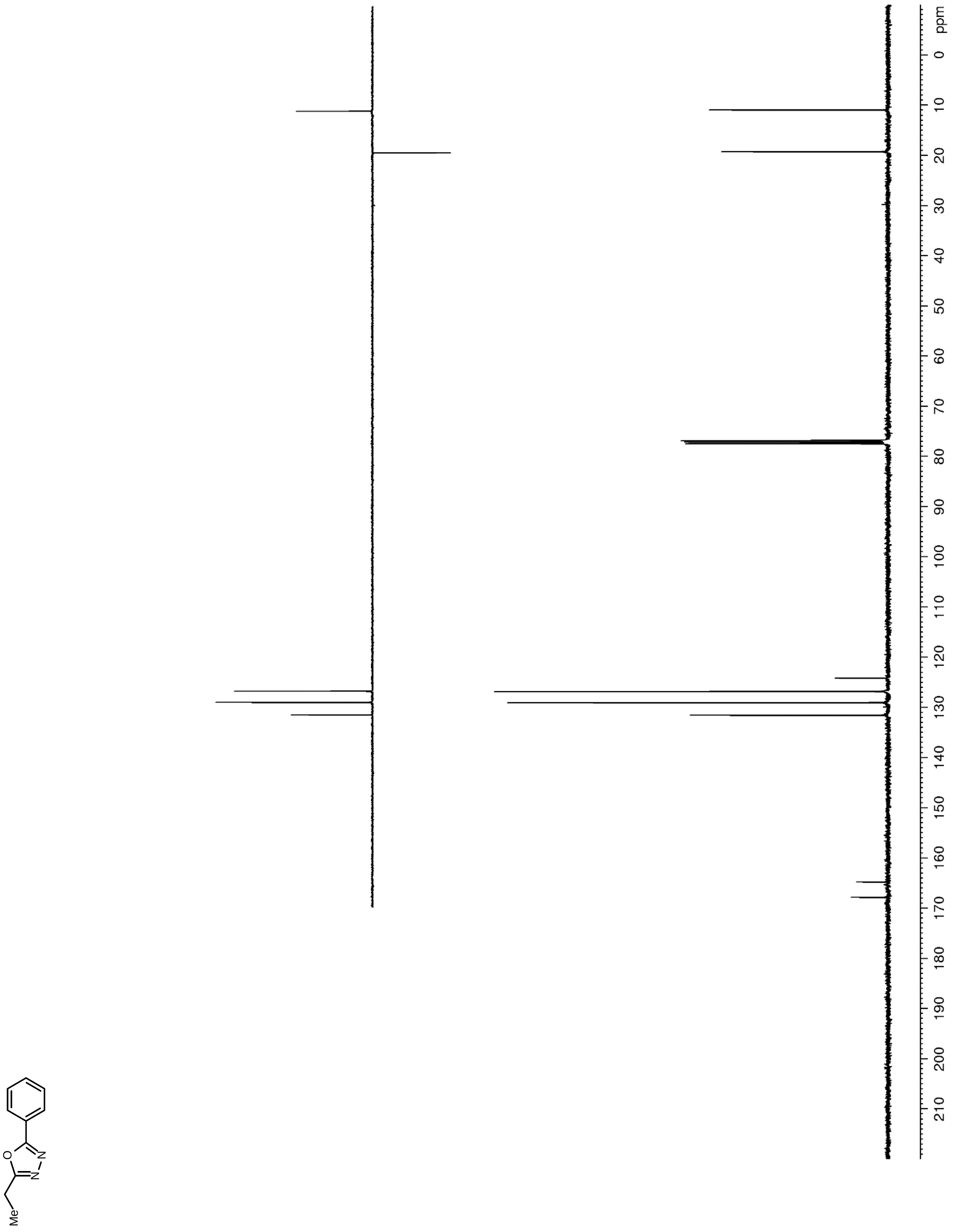

Figure 41. ^{1}H NMR (400 MHz, $CDCl_3$) of **13**

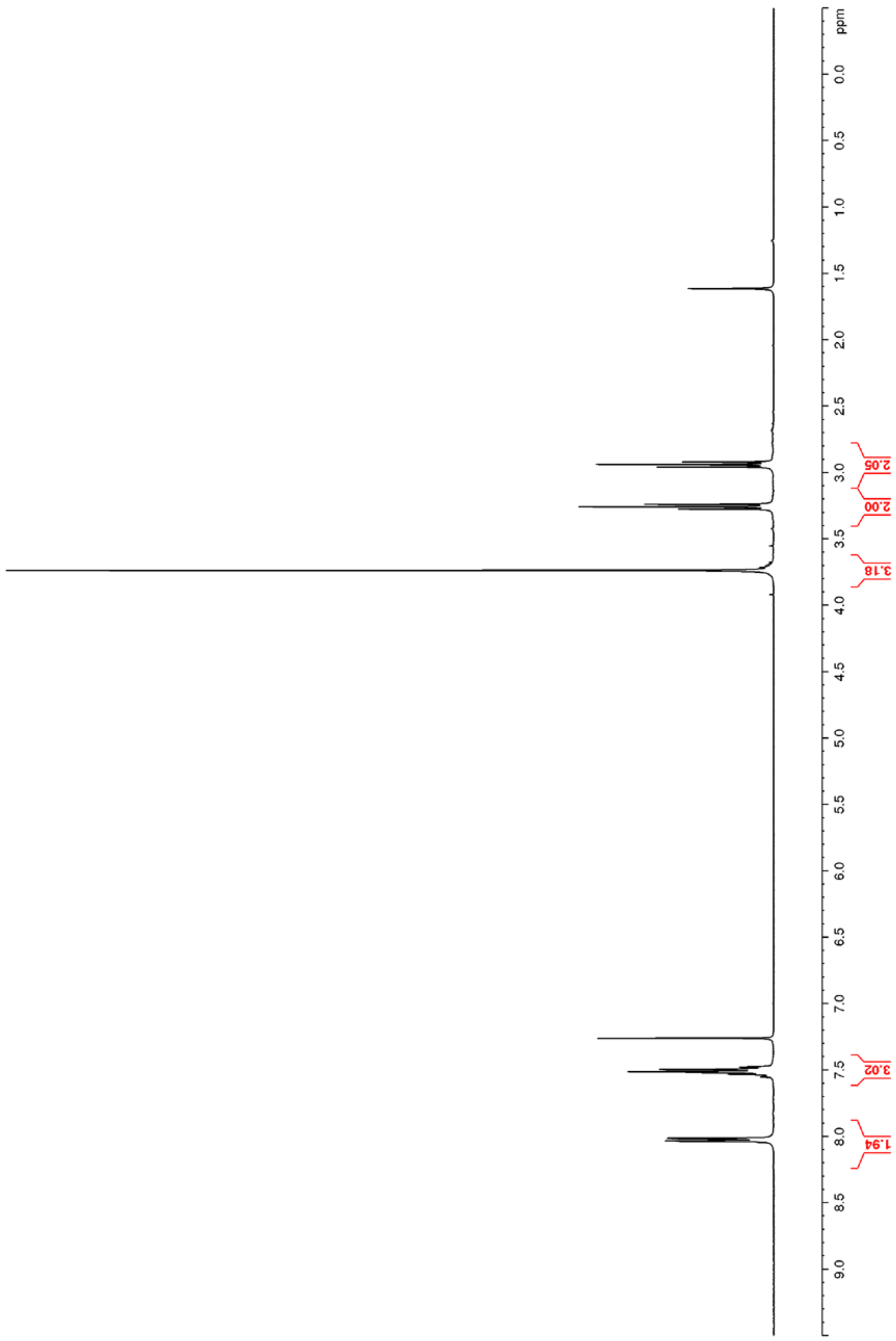

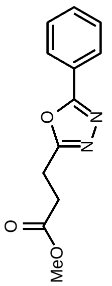

Figure 42. ^{13}C NMR (100 MHz, $CDCl_3$) of **13**

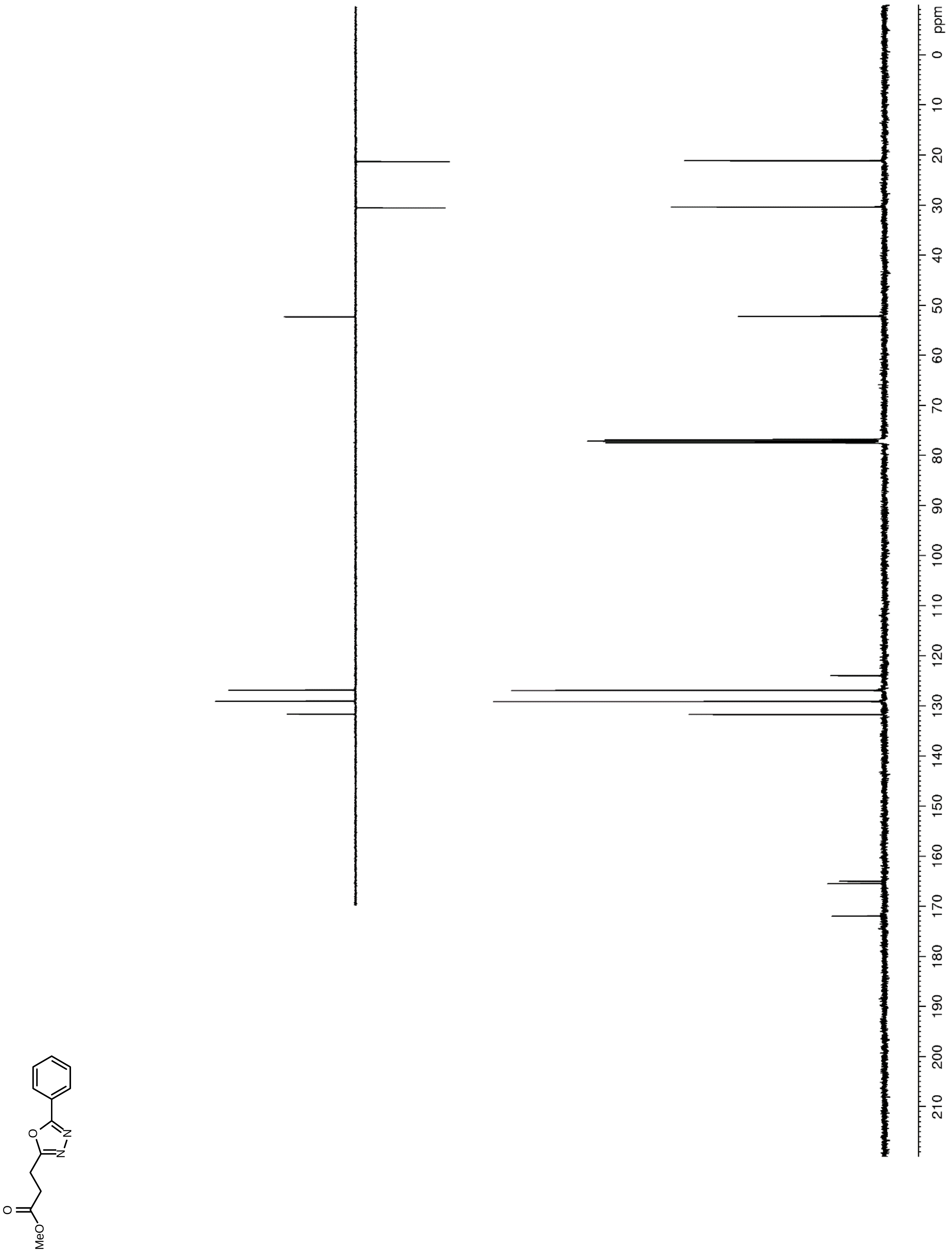

Figure 43. ^{1}H NMR (400 MHz, $CDCl_3$) of **14**

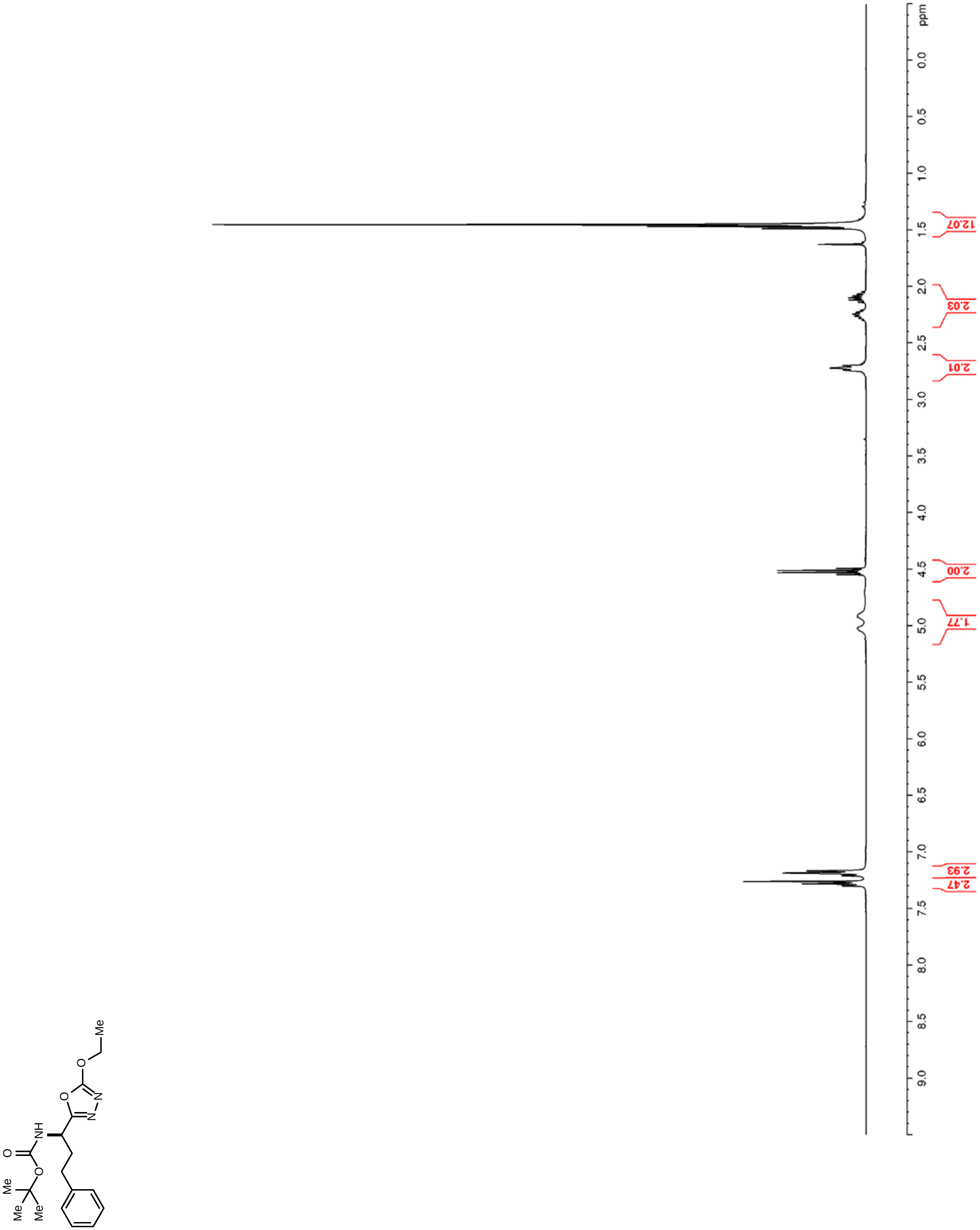

Figure 44. ^{13}C NMR (100 MHz, $CDCl_3$) of **14**

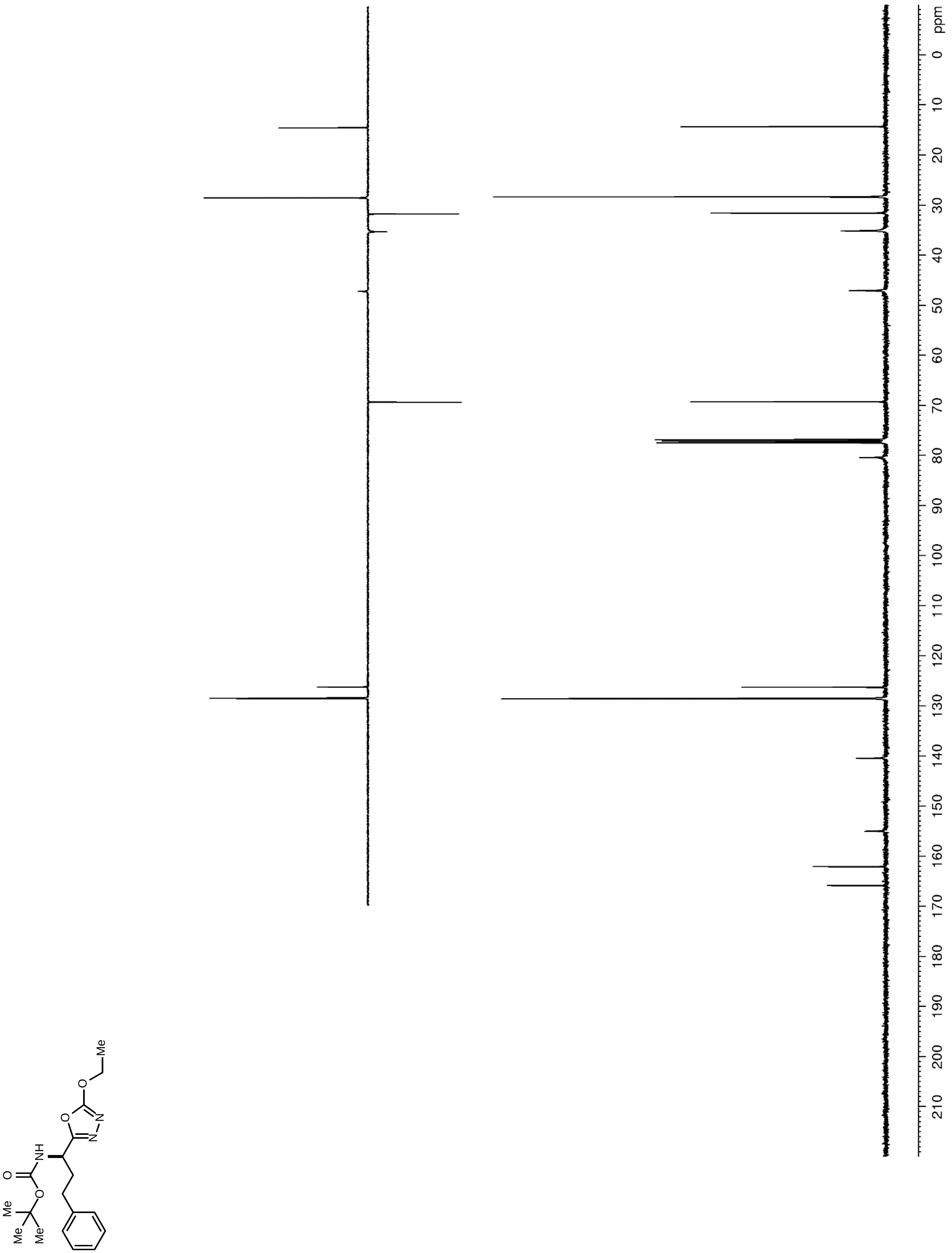

Figure 45. ^{1}H NMR (400 MHz, $CDCl_3$) of **15**

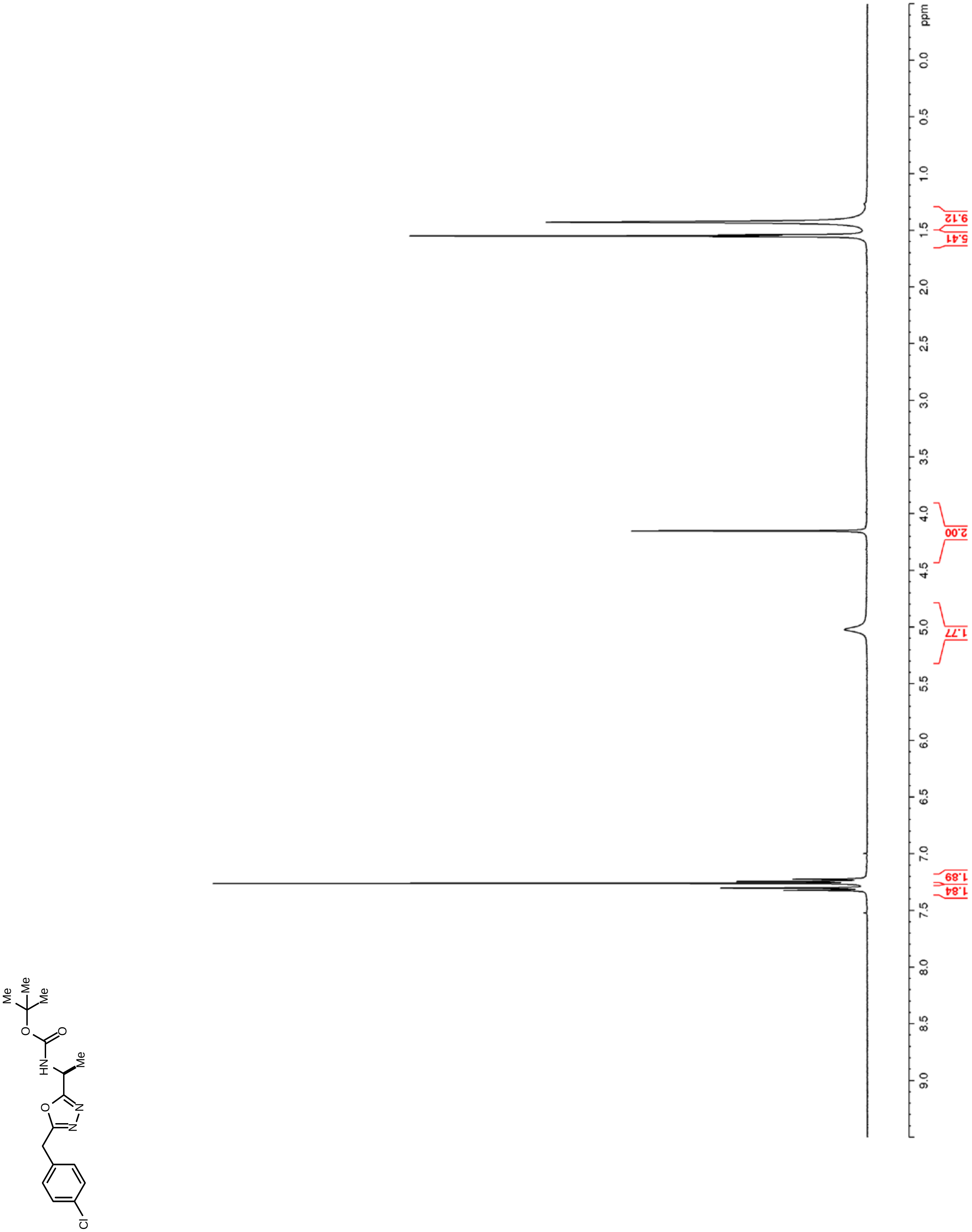

Figure 46. ^{13}C NMR (100 MHz, $CDCl_3$) of **15**

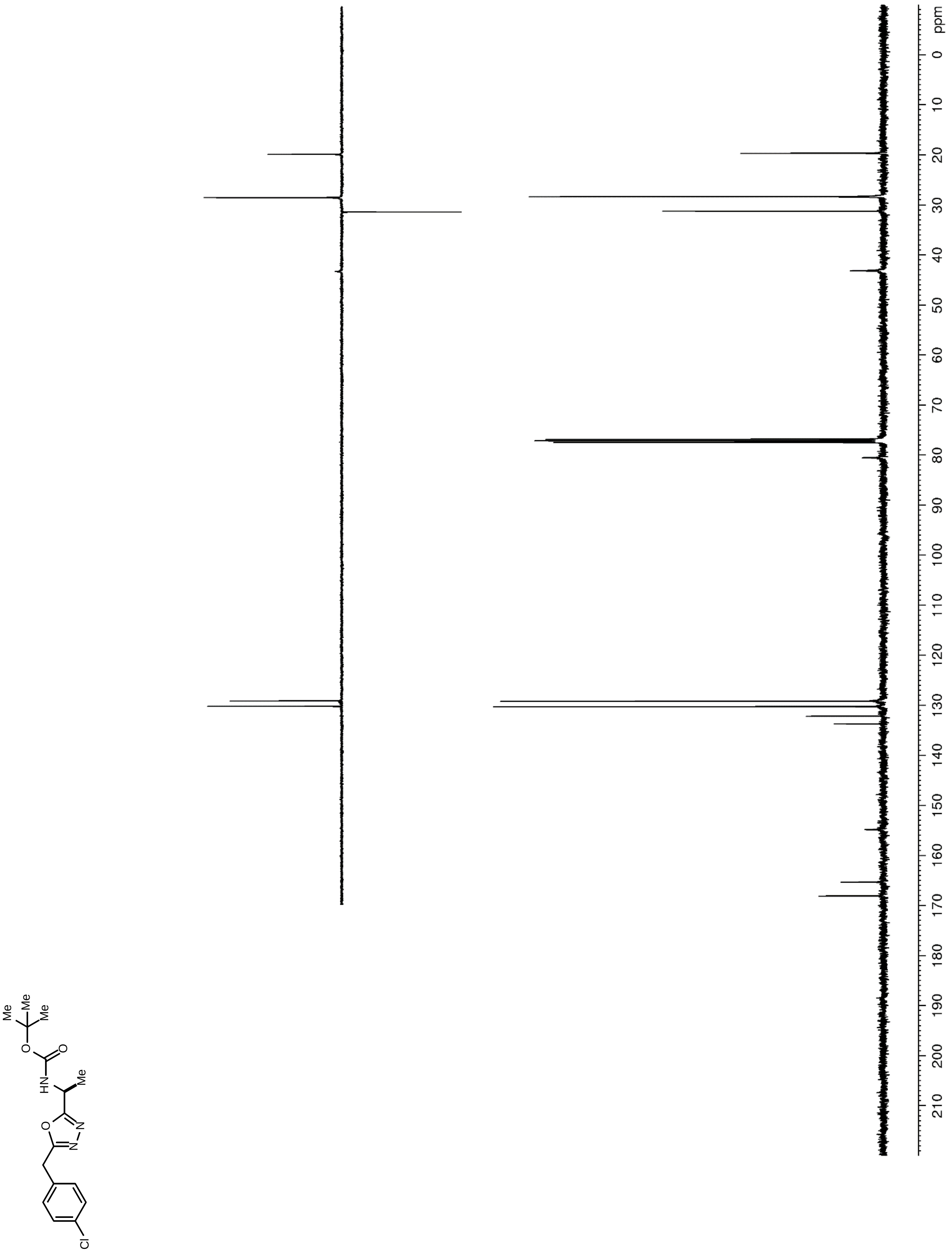

Figure 47. HPLC trace of **14**

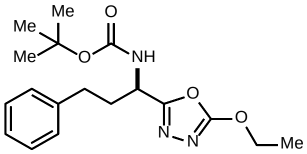

Racemate

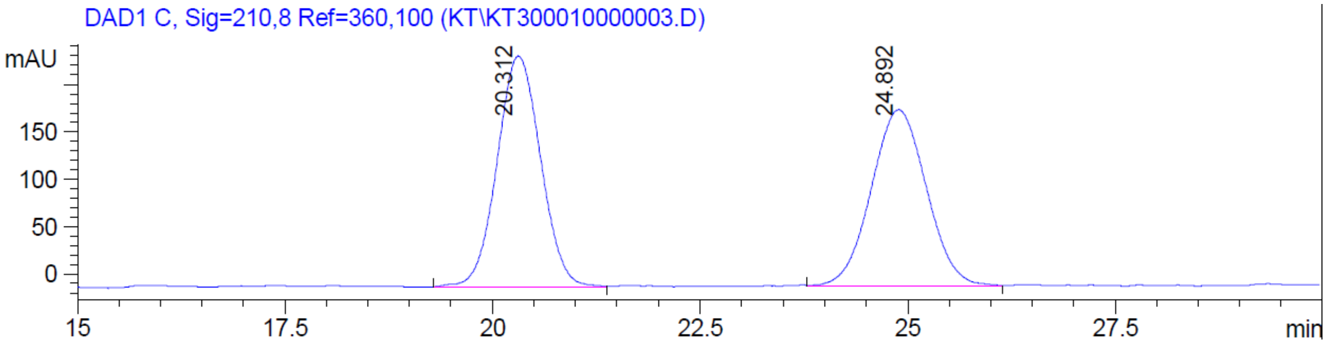

Peak #	RT [min]	Width [min]	Area	Area %
1	20.312	0.556	8884.089	50.73
2	24.892	0.633	8628.897	49.27

(*R*)- (>99% ee)

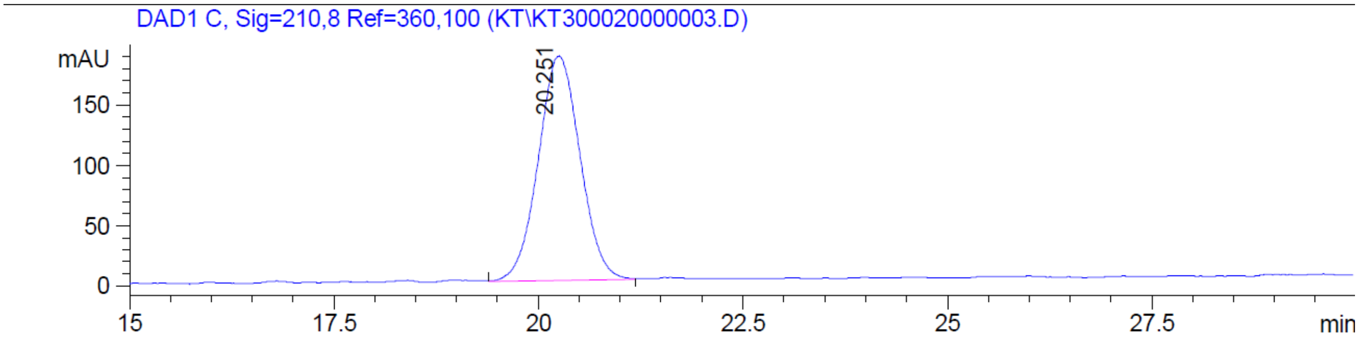

Peak #	RT [min]	Width [min]	Area	Area %
1	20.251	0.515	6601.235	100.00

Figure 48. HPLC trace of **15**

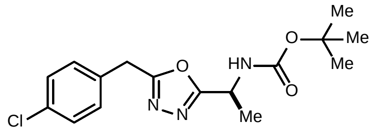

Racemate

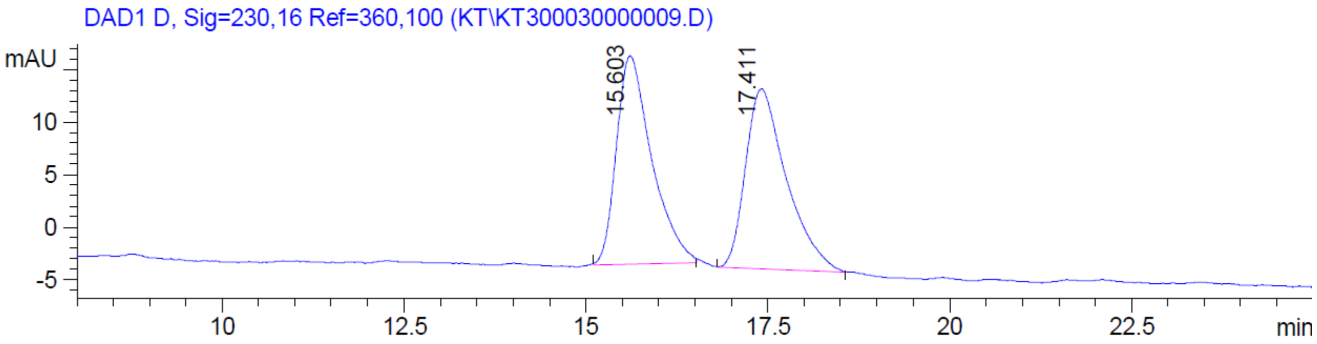

Peak #	RT [min]	Width [min]	Area	Area %
1	15.603	0.486	673.923	50.08
2	17.411	0.552	671.880	49.92

(*S*)- (>99% ee)

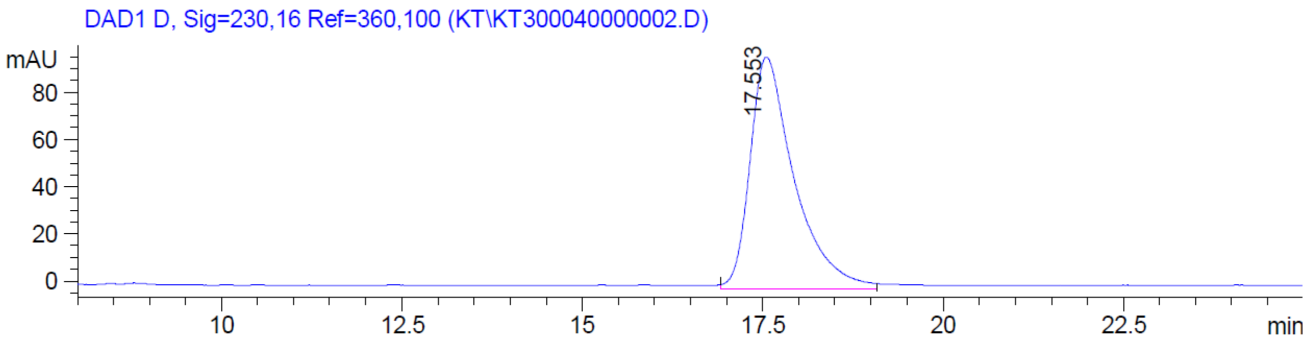

Peak #	RT [min]	Width [min]	Area	Area %
1	17.553	0.715	4216.073	100.00
